# Gold-Catalyzed Reactions between 2‑Alkenyl-1-arylalkynes and Nitrones via 1,2-C,N-Difunctionalizations Together with the C=C Double Bond Cleavage

**DOI:** 10.1021/acs.joc.5c02769

**Published:** 2025-12-12

**Authors:** Vikas Ashokrao Sadaphal, Hsin-Ru Wu, Rai-Shung Liu

**Affiliations:** † Department of Chemistry, 34881 National Tsing Hua University, Hsinchu 30013, Taiwan, ROC; ‡ Department of Chemistry and College of Semiconductor Research, National Tsing Hua University, Hsinchu 30013, Taiwan, ROC

## Abstract

This work reports gold-catalyzed novel 1,2-C,N-difunctionalizations of 2-alkenyl-1-arylalkynes with nitrones, along with the C=C double bond cleavage of substrates. Our mechanistic analysis suggests that gold directs an initial attack of *N*-hydroxyaniline on such enynes, followed by a cascade reaction with one nitrone molecule before the C=C double bond cleavage.

## Graphical abstract

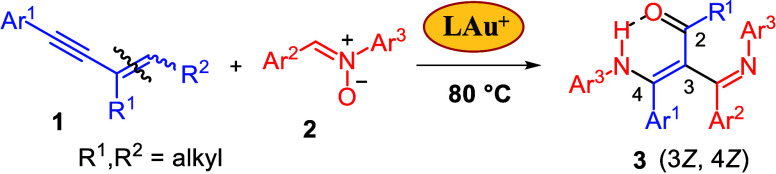

## Introduction

A significant advancement in gold catalysis is the oxidative transformation of alkynes using pyridine-based oxides or nitrones as external oxidants,^1^ enabling the generation of reactive α-oxo gold carbenes^2^ (**Int-2**). These carbene intermediates enable efficient 1,2-oxidative functionalizations,^3^ providing access to a wide variety of cyclic and acyclic molecules (**I** and **ll**) depending on the presence of external or internal nucleophiles (Scheme 1, eqs 1 and 2). In the context of nitrones as the oxidants, a unique oxoarylation,^4^ occurs with a 3,3-sigmatropic shift of key alkenylgold intermediates^5^ (**Int-1**), thereby highlighting the reaction diversity (Scheme 1, eq 3). To seek a novel reaction route using nitrones, we report here an unprecedented gold-catalyzed 1,2-C,N-difunctionalizations of 2-alkenyl-1-arylalkyne substrates (**1**), along with the C=C double bond cleavage,^6^ leading to the formation of highly substituted 4-amino-3-(iminomethyl)­but-3-en-2-one derivatives **3** in a specific (3*Z*,4*Z*)-configuration (Scheme 1, eq 4). Control experiments provide strong evidence that the C=C double bond cleavage of enynes (**1**) occurs in the last few steps. Our mechanistic studies support a pathway involving an initial attack of *N*-hydroxyaniline at the gold-π-enynes, yielding a nucleophilic alkenylgold intermediate that further reacts with nitrones to furnish the observed products, and the C=C double bond is cleaved during this transformation.

**Scheme 1:**
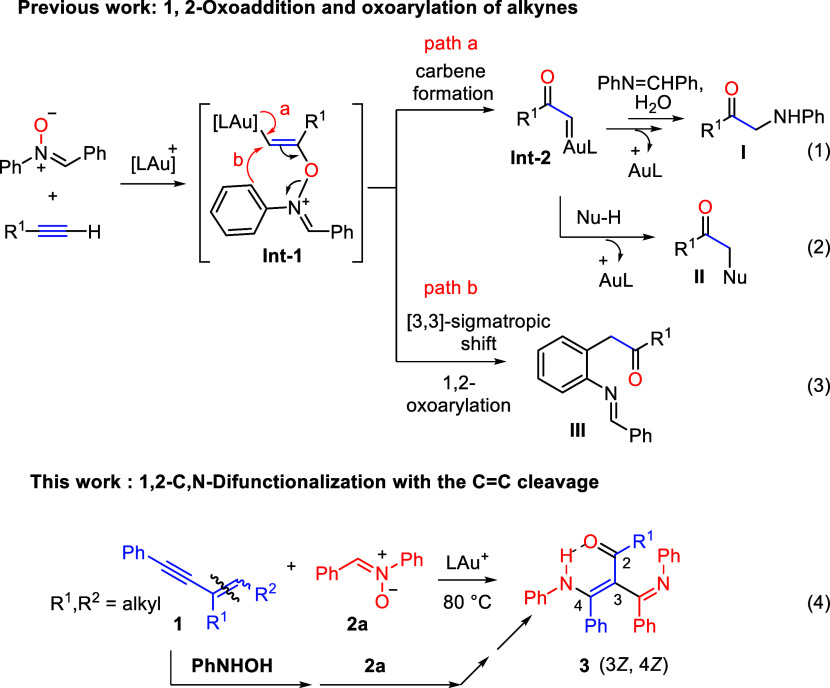
General Reactivity of Alkynes with Nitrones

## Results and Discussion

Table 1 shows the optimized conditions between (3-methylbut-3-en-1-yn-1-yl)­benzene **1a** (1.0 equiv) with nitrone **2a** (3.0 equiv) using various gold catalysts; initial results were obtained with JohnPhosAuCl/AgSbF_6_ in wet 1,2-dichloroethane (1,2-DCE) at 80 °C, affording the desired product **3a** in 64% yield with (3*Z*,4*Z*) stereoselectivity (entry 1); diazene oxide **2a**′ was obtained in 0.28 equiv. This wet DCE solution was used with freshly distilled DCE containing H_2_O (2.0 equiv). Using other phosphine ligands as in LAuCl/AgSbF_6_, such as L = XPhos (2-dicyclohexylphosphino-2′,4′,6′-triisopropylbiphenyl), PPh_3_, and (PhO)_3_P, resulted in the formation of the desired product **3a** in relatively low yields, 37–52% (entries 2–4). For other gold catalysts, LAuCl/AgSbF_6_ (L = IPr and P­(2,4-*t*-Bu_2_PhO)_3_), compound **3a** was obtained in 41 and 48% yields, respectively (entries 5 and 6). Furthermore, silver-free JohnPhosAuCl/NaBARF (BARF = [B­{3,5-(CF_3_)_2_C_6_H_3_}_4_]) was prepared, which failed to yield target compound **3a** (entry 7). Variations of the silver salts for JohnPhosAuCl (AgX, X = NTf_2_ and OTf) led to the formation of compound **3a** in 58 and 50% yield, respectively (entries 8 and 9). JohnPhosAuCl/AgSbF_6_ in wet toluene delivered product **3a** in 45% yield (entry 10). AgSbF_6_ alone in wet 1,2-DCE was found to be catalytically inactive (entry 11). We also performed the reaction with dry DCE over molecular sieves; the yield of target **3a** was decreased by 21% (entry 12). The molecular structure of compound **3a** was inferred from the X-ray diffraction of its relative **4e**, which shows a (3*Z*,4*Z*)-configuration.^7^

**Table 1:**
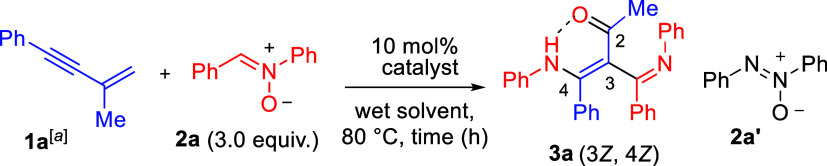
Optimization of Reaction Conditions^a^

entry	catalyst	solvent	time (h)	yield^b^ **3a** ^b^
1	L_1_AuCl/AgSbF_6_ ^c^	DCE	17	64^d^
2	XphosAuCl/AgSbF_6_ ^e^	DCE	19	52
3	PPh_3_AuCl/AgSbF_6_	DCE	22	44
4	(PhO)_3_PAuCl/AgSbF_6_	DCE	24	37
5	IPrAuCl/AgSbF_6_	DCE	18	41
6	L_2_AuCl/AgSbF_6_ ^f^	DCE	23	48
7	L_1_AuCl/NaBARF	DCE	24	
8	L_1_AuCl/AgNTf_2_	DCE	18	58
9	L_1_AuCl/AgOTf	DCE	22	50
10	L_1_AuCl/AgSbF_6_	toluene	24	45
11	AgSbF_6_	DCE	20	
12	L_1_AuCl/AgSbF_6_ ^g^	DCE	18	43

a [**1a**] = 0.14 M.

b Product yields are obtained after purification from a silica column.

c L_1_ = P­(^*t*^Bu)_2_(*o*-biphenyl).

d Species **2a**′ was isolated in 0.28 equiv in entry 1.

e Xphos = (2-dicyclohexylphosphino-2′,4′,6′-triisopropylbiphenyl).

f L_2_ = P­(2,4-*t*-Bu_2_PhO)_3_.

g Reaction in the presence of dry DCE over 4 Å MS. DCE = 1,2-dichloroethane.

Under the optimized condition in Table 1 (entry 1), the substrate scope was explored using various substituted 2-alkenyl-1-arylalkynes (**1**) and nitrone (**2a**) in the presence of 10 mol % JohnPhosAuCl/AgSbF_6_; the results are summarized in Scheme 2. Two tautomers are detectable for those products when two aryl groups, C_6_H_3_XY and Ph, are not equivalent. This structural assignment was inferred from two distinct proton NMR peaks in the NH–O=C regions. Tautomers such as keto–enol forms have the same anion geometry, which typically shows identical chemoselectivity. Initially, we prepared substrates **1b**–**1e** bearing various 4-*para*-phenyl substituents (X = Me, OMe, Cl, and Br), their standard operations yielded the desired products **3b**–**3e** in 61–70% yields with a tautomeric ratio (tr) (tr *≥* 1.1:1). Electron-rich groups such as methyl and methoxy provided slightly higher yields compared to electron-withdrawing substituents. These new reactions were also applicable to substrates **1f** and **1g** containing 3-*meta*-phenyl substituents (Y = Me and Cl), which delivered the desired products **3f** and **3g** in 65 and 57% yields, with tr = 1.25:1 and 2:1, respectively. A similar trend was observed for the *meta*-substituted analogues, wherein the electron-rich methyl substituent afforded a comparatively higher yield. We further synthesized a 2-naphthalene-substituted enyne substrate **1h**, affording the corresponding product **3h** in 65% yield with a tautomeric ratio of 2:1. Additionally, the enyne substrate **1i** bearing an ethyl substituent (R^1^ **=** Et) furnished the desired product **3i** in 69% yield with tr > 25:1. In contrast, the enyne substrate **1j** featuring a longer *n*-pentyl chain (R^1^ = *n*-pentyl) failed to yield the desired product under the standard reaction conditions.

**Scheme 2:**
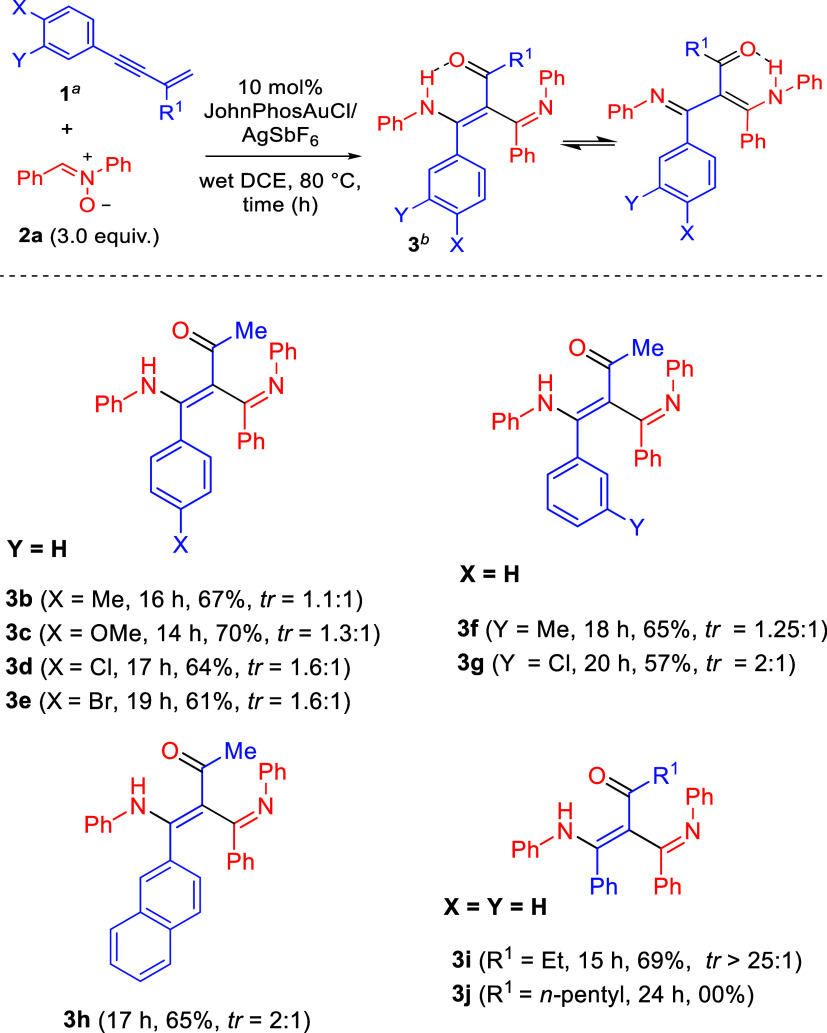
Reactions of Various (3-Methylbut-3-en-1-yn-1-yl)­benzene

a [**1**] = 0.14 M.
b Product yields are obtained after purification from a silica column.

The substrate scope was further examined by using various nitrones **2** with enyne **1a**; the results are provided in Scheme 3. We first tested the reactions on nitrones **2b** and **2c**, bearing different *para*-phenyl imines (Ar^1^ = *p*-C_6_H_4_X; X = Cl and Me), which delivered the desired products **4a** and **4b** in 64 and 58% yields, with tr = 1.6:1 and 1.1:1, respectively. Notably, the electron-deficient chloro substituent provided a slightly higher yield compared to that of the electron-donating methyl group. We also synthesized *meta*-phenyl imine-containing nitrones **2d** and **2e**, (Ar^1^ = *m*-C_6_H_4_X; X = Cl and Me), further affording desired products **4c** and **4d** in 62 and 55% yields, with a moderate tr ratio of 2:1 and 1.1:1, respectively. Similar to the *para*-substituted analogues, the electron-withdrawing chloro substituent resulted in a higher yield than that of the electron-donating methyl group. These new reactions were also applicable to nitrones bearing *para*-substituted anilines **2f**–**2h** (Ar^2^ = *p*-C_6_H_4_X; X = Cl, Br, and Me), delivering the expected products **4e**–**4g** in 57–64% yields, yielding only one tautomer with an electron-rich methyl group being more efficient. Nitrones **2i**–**2k** bearing *meta*-substituted anilines (Ar^2^ = *m*-C_6_H_4_X; X = Cl, Br, and Me) were also synthesized, yielding products **4h**–**4j** in 61–67% yields. In the case of the *meta*-substituted analogues, a mixed trend in the reactivity was observed. The molecular structure of compound **4e** was determined by an X-ray diffraction study.^7^ As shown in this ORTEP image, this C–C bond is substituted with four bulky substituents, including MeC=O, =CPhNH*p*Ar (Ar = 4-ClC_6_H_4_), =NAr, and Ph; a long C–C bond distance (1.50 Å) is observed, showing an easy C–C bond rotation.

**Scheme 3:**
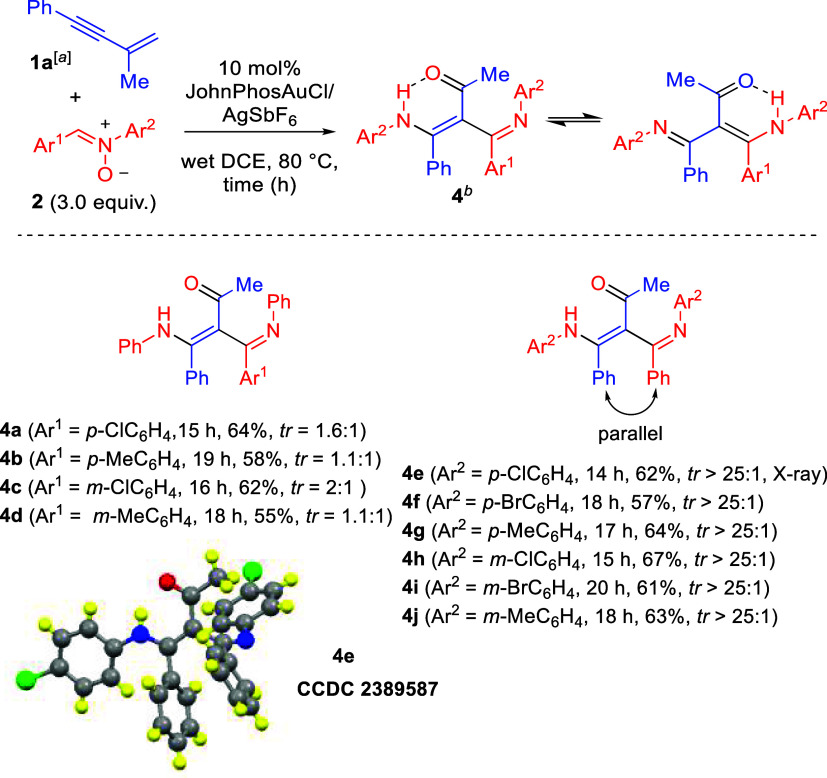
Catalytic Reactions with Various Nitrones

[a] [**1a**] = 0.14 M.
b Product yields are obtained after purification from a silica column.

The control experiments were carried out to gain insight into the mechanism underlying the formation of product **3** (Scheme 4). Initially, we carried out the reaction of 4-phenylbut-3-yn-2-one **1k** and standard nitrone **2a** (3.0 equiv) with JohnPhosAuCl/AgSbF_6_ (10 mol %), in wet 1,2-DCE at 80 °C, affording highly substituted pyrrole derivative product **5a** in 69% yield (Scheme 4, eq 5). The molecular structure of compound **5a** was confirmed by an X-ray diffraction study.^7^ The reaction of (3-methylpent-3-en-1-yn-1-yl)­benzene **1l** with nitrone **2a** (3.0 equiv) yielded 4-amino-3-(iminomethyl)­but-3-en-2-one derivative **3a** as only one tautomer with 61% yield (Scheme 4, eq 6). This outcome confirms cleavage of the C=C double bond of substrate **1l**. We also clarify that our primary target **3a** is not convertible to another product **5a** in the absence and presence of nitrone **2a** (0 or 0.5 equiv, Scheme 4, eq 6). Accordingly, the C=C bond cleavage will not take place in the first step; in other words, our system will not involve enone **1k** in the initial step. We prepared C_6_D_5_NHOH and C_6_D_5_NH_2_ to clarify their roles in this catalytic system; as shown in Scheme 4, eqs 7 and 8; only C_6_D_5_NHOH gave deuterated sample ***d***_**5**_ **-3a** together with ***d***_**0**_ **-3a** according to ^1^H NMR and mass analysis (see Supporting Information); the ratio of ***d***_**5**_ **-3a**/***d***_**0**_ **-3a** is 1.0:1.4 much higher than the initial ratio of ***d***_**5**_-**2l**/**2a** = 1:4. In contrast, our reaction will not involve aniline as a reaction partner, because deuterated product ***d***_**5**_ **-3a** could not be identified by high-resolution mass. In the presence of a gold catalyst, mixing a 1:1 molar mixture of C_6_D_5_NHOH with PhCHN­(O)=Ph in hot and wet DCE at 80 °C in 1 h yielded a mixture of PhCHNHOH and C_6_D_5_NHOH in a 1:2 molar ratio, showing an exchange of PhNHOH.

**Scheme 4:**
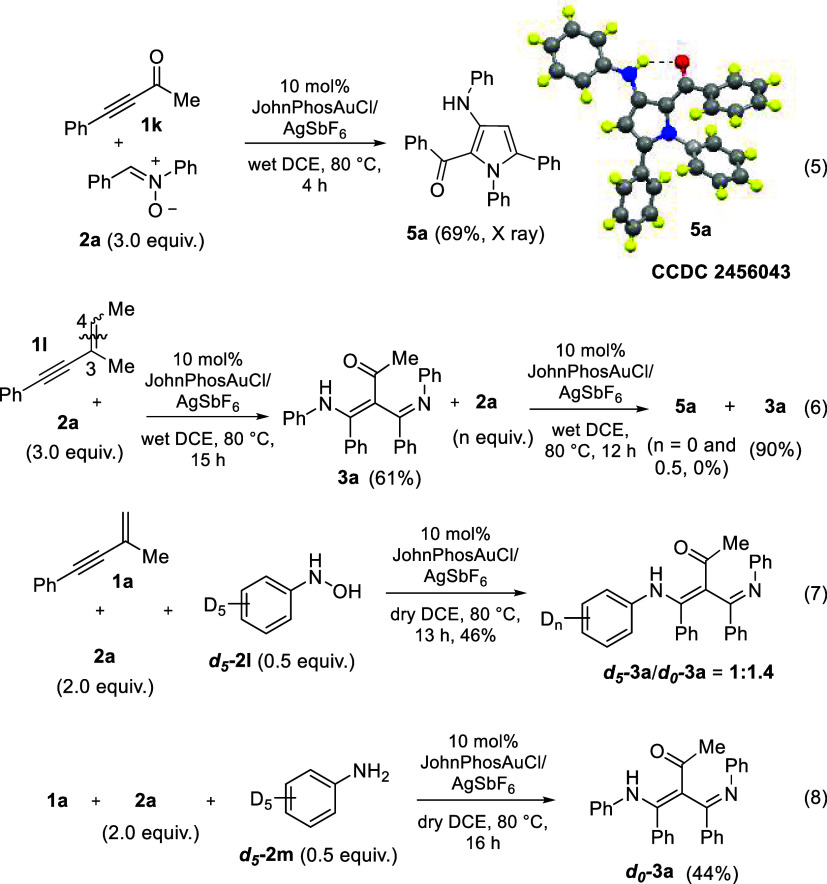
Control Experiments

Scheme 5 illustrates the gram-scale synthesis of compound **3a**. Treatment of (3-methylbut-3-en-1-yn-1-yl)­benzene (**1a**, 1.0 equiv) with nitrone **2a** (3.0 equiv) in the presence of JohnPhosAuCl/AgSbF_6_ (10 mol %) in wet 1,2-DCE at 80 °C afforded the desired product **3a** in 61% yield (1.78 g), with exclusive (3*Z*,4*Z*)-stereoselectivity.

**Scheme 5:**
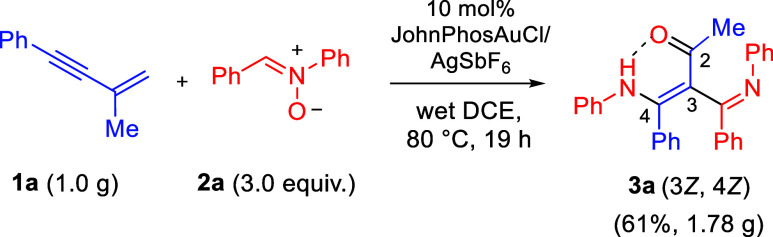
Gram Scale Synthesis

To demonstrate the potential of this new methodology, we carried out chemical functionalizations using a representative compound, **3a**; the results are summarized in Scheme 6. Initially, we treated compound **3a** with H_2_, Pd/C in EtOH, affording hydrogenation product **6a** in 43% yield as a single diastereomer (dr > 25:1) in racemic form, which resulted in C–N bond cleavage. The molecular structure of compound **6a** was determined by an X-ray diffraction study.^7^ Subsequent reduction of compound **3a** with LiAlH_4_ (5.0 equiv) delivered product **6b** in 74% yield. The molecular structure of compound **6b** was elucidated using an X-ray diffraction study.^7^ N-Methylation of compound **3a** furnished its *N*-methyl derivative **6c** with a *Z*/*E* ratio of 2.5:1. We postulate a delocalization between PhNMe and the enone moiety to give a partial C–N double bond.

**Scheme 6:**
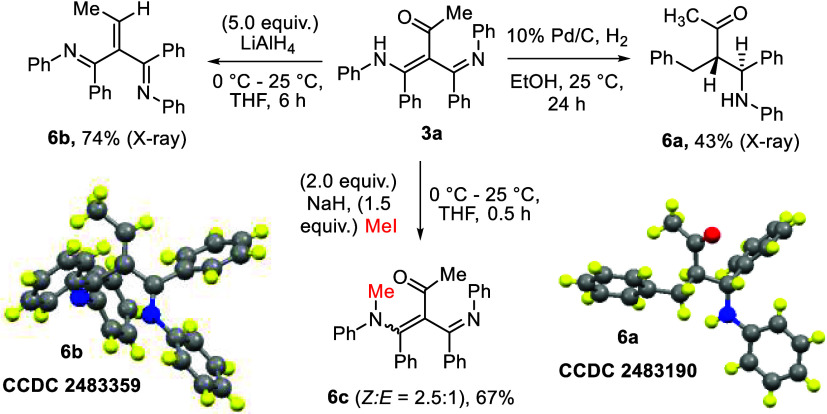
Chemical Functionalization

Finally, gas chromatography-mass spectrometry (GC-MS) was employed to analyze small molecules generated from the C=C bond cleavage; we have identified PhCHO and, importantly, PhCH=CH_2_ according to its exact mass 104.06192 (calcd 104.06205, see Supporting Information). A plausible reaction mechanism was proposed in Scheme 7 based on control experiments and GC-MS analysis. In wet DCE, nitrone will be hydrolyzed to form benzaldehyde and PhNHOH. Initially, a nucleophilic attack of PhNHOH on the gold-activated alkyne of substrate **1a** results in the formation of vinylgold intermediate **A**. This alkenylgold moiety contains an electron-rich *N*-hydroxyamino group, which further reacts with one nitrone molecule to form species **B** in which LAu^+^ subsequently undergoes a σ–π–σ migration to form species **C**. An intramolecular cyclization of species **C** likely forms species **D** which proceeds through a cleavage of the weak N–O bond to yield intermediate **E**. We postulate that species **E** undergoes a retro-carbonyl alkylation to form species **F** and CH_2_Au^–^ anion; the latter was protonated by a weak acid PhNH_2_OH^+^ to form MeAu, which then condenses with PhCHO to yield PhCH=CH_2_. Finally, intermediate **F** is expected to be reduced by PhNH_2_OH to form the observed product **3a**. Our isolation of diazene oxide (**2a**′) is produced from PhNHOH and PhNO.^1f^

**Scheme 7:**
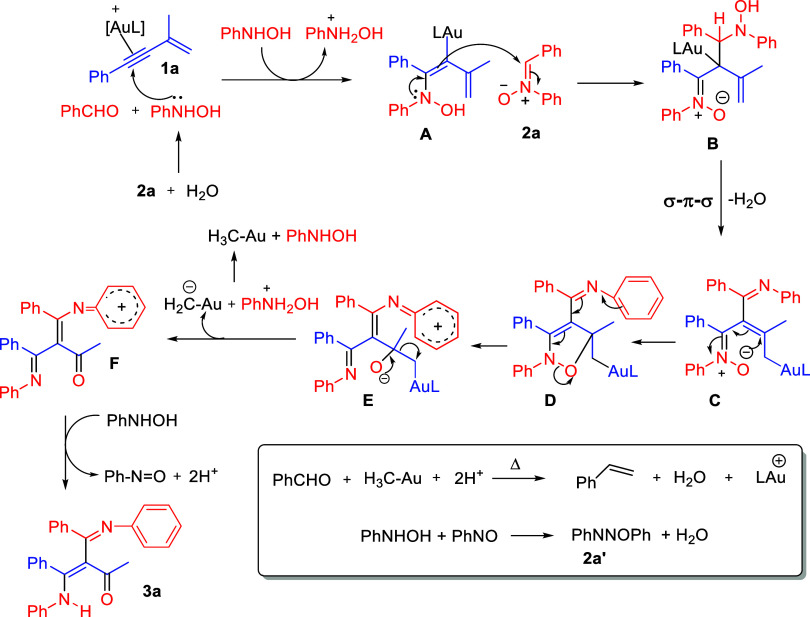
Plausible Reaction Mechanism

## Conclusions

In summary, this study presents a gold-catalyzed transformation of (3-methylbut-3-en-1-yn-1-yl)­benzene **1a** with nitrones **2**, affording highly substituted (*Z*)-4-amino-3-(iminomethyl)­but-3-en-2-one derivatives **3** with excellent tautomeric selectivity (tr > 25:1). Control experiments provide compelling evidence of an oxidative cleavage of the C=C double bond, highlighting the unique reactivity enabled by the gold catalyst.

## Experimental Section

### General Information

Unless otherwise noted, all of the reactions for the preparation of the substrates were performed in oven-dried glassware under a nitrogen atmosphere with freshly distilled solvents. The catalytic reactions were performed under a nitrogen atmosphere. Toluene was distilled from CaH_2_ under nitrogen. Tetrahydrofuran (THF), 1,2-dichloroethane, and dichloromethane (DCM) were distilled from Na metal under nitrogen. Other solvents such as triethylamine, 1,2-dichloroethane, EtOH, ethyl acetate (EA), acetonitrile, and hexane were used from commercial sources without further distillation. All other commercial reagents were used without further purification unless otherwise indicated. ^1^H NMR and ^13^C NMR spectra were recorded on Varian 700 and 500 MHz, Bruker 400 MHz spectrometers using chloroform-*d* (CDCl_3_) as solvent and Me_4_Si as an internal standard, with chemical shifts (δ) and spin–spin coupling constants (*J*). The following abbreviations were used to show the multiplicities: s, singlet; d, doublet; t, triplet; q, quadruplet; dd, doublet of doublet; m, multiplet. All heating reactions were carried out with an oil bath as the heat source. Reactions were magnetically stirred and monitored by thin-layer chromatography (TLC) carried out on a 0.25 mm E. Merck silica gel plate (60f-254) using UV light as the visualizing agent. High-resolution mass spectrometry (HRMS) analysis data were measured on a JMST100LP4G (JEOL) mass spectrometer or a time-of-flight (TOF) mass analyzer equipped with an electrospray ionization (ESI) source, JEOL Model: JMS-T200GC AccuTOF GCx equipped with a field desorption (FD) source. Single-crystal X-ray diffraction intensity data were collected on a Bruker X8 APEX diffractometer equipped with a charge-coupled device (CCD) area detector and Mo Kα radiation (λ = 0.71073 Å) at 100 K; all data calculations were performed by using the PC version of the APEX2 program package. Gas Chromatography-Mass Spectrometry (GC-MS) data were measured on a GC-TOF mass analyzer equipped with an EI source, JEOL Model: JMS-T2000GC, having column: DB-5 ms, injection volume: 500 (μL) air, and split mode/split (1:10).

### Preparation and Characterization of Substrates

All nitrones (**2a**–**2j**) were prepared according to the reported procedure^[s2]^ (see Supporting Information).

### Procedure for the Synthesis of (3-Methylbut-3-en-1-yn-1-yl)­benzene (**1a**):^[s1]^

A two-necked flask was charged with Pd­(PPh_3_)_2_Cl_2_ (137.4 mg, 0.19 mmol, 2 mol %) and CuI (93.2 mg, 0.48 mmol, 5 mol %), evacuated and backfilled with N_2_ (repeated for three times), THF (10 mL), Et_3_N (10 mL) was added at room temperature under a stream of nitrogen. Then, alkyne (1.0 g, 9.79 mmol, 1.0 equiv) and 2-bromopropene (1.4 g, 11.7 mmol, 1.2 equiv) were added, and the reaction was put into a preheated oil bath at 50 °C for 12 h under a nitrogen atmosphere. Then saturated aq. NH_4_Cl (10 mL) was added to the mixture, and the aqueous layer was extracted with EtOAc (3 × 10 mL). The combined organic phase was washed with 10% aq. HCl (20 mL) and brine (20 mL), dried over anhydrous Na_2_SO_4_, filtered, and concentrated under vacuum. The residue was then purified by flash column chromatography eluting with (EA/hexane = 2:98) to give (3-methylbut-3-en-1-yn-1-yl)­benzene **1a** (0.91 g, 6.45 mmol, 66%) as a pale yellow liquid.

All other enynes (**1b**–**1i**, **1l**) were prepared according to the above procedure. Substrate **1j** was prepared according to the procedure reported in the literature.^[s1‑d]^ Substrate **1k** was prepared according to the procedure reported in the literature (see Supporting Information).^[s1‑e]^

#### (3-Methylbut-3-en-1-yn-1-yl)­benzene (**1a**)

Purified by silica gel column, ethyl acetate/hexane (2:98) as eluent; pale yellow liquid (0.91 g, 6.45 mmol, 66%); ^1^H NMR (500 MHz, CDCl_3_): δ 7.47–7.45 (m, 2H), 7.33–7.30 (m, 3H), 5.41 (s, 1H), 5.31 (s, 1H), 2.00 (s, 3H); ^13^C­{^1^H} NMR (125 MHz, CDCl_3_): δ 131.5, 128.2, 128.1, 126.8, 123.2, 121.9, 90.5, 88.3, 23.4; HRMS (FD) *m*/*z*: [M]^+^ calcd for C_11_H_10_: 142.0777; found: 142.0774.

#### (3-Methylenepent-1-yn-1-yl)­benzene (**1i**)

Purified by silica gel column, ethyl acetate/hexane (2:98) as eluent; pale yellow liquid (0.98 g, 6.27 mmol, 64%); ^1^H NMR (500 MHz, CDCl_3_): δ 7.44 (t, *J* = 7.8 Hz, 2H), 7.32–7.29 (m, 3H), 5.40 (s, 1H), 5.30 (s, 1H), 2.27 (q, *J* = 14.9 and 7.4 Hz, 2H), 1.16 (t, *J* = 7.5 Hz, 3H); ^13^C­{^1^H} NMR (125 MHz, CDCl_3_): δ 133.2, 131.5, 128.2, 128.0, 123.3, 120.0, 89.8, 89.1, 30.9, 12.9; HRMS (ESI-TOF) *m*/*z*: [M + H] calcd for C_12_H_13_: 157.1017; found: 157.1018.

#### (3-Methylpent-3-en-1-yn-1-yl)­benzene (**1l**)

Purified by silica gel column, ethyl acetate/hexane (2:98) as eluent; pale yellow liquid (0.67 g, 4.29 mmol, 44%); ^1^H NMR (500 MHz, CDCl_3_): δ 7.40 (d, *J* = 7.8 Hz, 2H), 7.29–7.25 (m, 3H), 6.03–5.99 (m, 1H), 1.86 (s, 3H), 1.73 (d, *J* = 7.0 Hz, 3H); ^13^C­{^1^H} NMR (125 MHz, CDCl_3_): δ 132.8, 131.3, 128.1, 127.6, 123.7, 118.4, 92.5, 85.6, 16.8, 14.1; HRMS (FD) *m*/*z*: [M]^+^ calcd for C_12_H_12_: 156.0933; found: 156.0936.

### Procedure for the Synthesis of *N*-(Phenyl-*d*_5_)­hydroxylamine (***d***_**5**_ **-2l**):^[s3]^

Ammonium chloride (2.5 g, 46.8 mmol, 1.2 equiv) was placed in a 500 mL two-neck round-bottom flask with a mechanical stirrer and dissolved in H_2_O (40 mL). After that, a solution of nitrobenzene-*d*_5_ **s1** (5.0 g, 39.0 mmol, 1.0 equiv) in EtOH (10 mL) was added to the aqueous ammonium chloride. The resulting mixture was stirred vigorously at 25 °C, while zinc dust (5.61 g, 85.8 mmol, 2.2 equiv) was added in small portions at 0 °C. Finally, the mixture was stirred at 25 °C for 3 h before zinc oxide was filtered off and washed with EA (30 mL). The filtrate was extracted four times with 50 mL of EA. The combined organic layers were dried over MgSO_4_, and the solvent was evaporated under reduced pressure. The residue was washed with pentane (10 mL 2x) to afford *N*-(phenyl-*d*_5_)­hydroxylamine (***d***_**5**_ **-2l**) in 73% yield (3.2 g, 28.4 mmol) as a white cotton wool-like solid. The obtained product was stored at 0 °C to avoid decomposition.

#### *N*-(Phenyl-*d*_5_)­hydroxylamine (***d***_**5**_ **-2l**)

Purified by pentane wash (10 mL 2×); colorless solid (3.2 g, 28.4 mmol, 73%); ^1^H NMR (500 MHz, CDCl_3_): δ 6.76 (s, 1H), 5.50 (s, 1H); ^13^C­{^1^H} NMR (125 MHz, CDCl_3_): δ 149.2, 128.4 (t), 122.0 (t), 114.4 (t); HRMS (FD) *m*/*z*: [M]^+^ calcd for C_6_H_2_NOD_5_: 114.0808; found: 114.0811.

### Procedure for the Synthesis of Benzen-*d*_5_-amine (***d***_**5**_ **-2m**):^[s4]^

In a round-bottomed flask, the nitrobenzene-*d*_5_ **s1** (1.0 g, 7.80 mmol, 1.0 equiv) and *N*,*N-*diisopropylethylamine (5.0 g, 39.0 mmol, 5.0 equiv) were dissolved in acetonitrile solvent (10 mL) under magnetic stirring and a nitrogen atmosphere. A solution of freshly distilled HSiCl_3_ (3.70 g, 27.3 mmol, 3.5 equiv) in 5 mL of acetonitrile solvent was prepared separately, and it was added dropwise to the first solution over 10 min at 0 °C. After stirring the reaction mixture for 18 h, 15 mL of a saturated solution of NaHCO_3_ was added dropwise, and the biphasic mixture was allowed to stir for 30 min. The crude mixture was extracted with ethyl acetate, dried over Na_2_SO_4_, filtered, and then dried under reduced pressure to afford the crude product. The crude product was then purified by flash column chromatography, eluting with (EA/hexane = 50:50) to give benzen-*d*_5_-amine ***d***_**5**_ **-2m** (0.67 g, 6.83 mmol, 87%) as a yellow liquid.

#### Benzen-*d*_5_-amine (***d***_**5**_ **-2m**)

Purified by silica gel column, ethyl acetate/hexane (50:50) as eluent; pale orange liquid (0.67 g, 6.83 mmol, 87%); ^1^H NMR (700 MHz, CDCl_3_): δ 3.57 (s, 2H); ^13^C­{^1^H} NMR (175 MHz, CDCl_3_): δ 146.1, 128.7 (t), 118.0 (t), 114.6 (t); HRMS (FD) *m*/*z*: [M]^+^ calcd for C_6_H_2_ND_5_: 98.0859; found: 98.0860.

### Standard Catalytic Reaction Procedure for the Synthesis of (*Z*)-4-Phenyl-3-((*E*)-phenyl­(phenylimino)­methyl)-4-(phenylamino)­but-3-en-2-one (**3a**)

A 10 mL flask was charged with JohnPhos gold chloride (19 mg, 0.03 mmol, 0.1 equiv) and AgSbF_6_ (12.0 mg, 0.03 mmol, 0.1 equiv), and to this mixture was added wet 1,2-dichloroethane (0.5 mL). The resulting mixture was stirred at room temperature for 5 min. To this mixture was added dropwise a wet 1,2-dichloroethane (2.0 mL) solution of (3-methylbut-3-en-1-yn-1-yl)­benzene **1a** (50 mg, 0.35 mmol, 1 equiv) and (*Z*)-*N*,1-diphenylmethanimine oxide **2a** (208 mg, 1.05 mmol, 3.0 equiv) at room temperature. After addition, the reaction mixture was stirred at 80 °C in an oil bath for 17 h. The reaction mixture was filtered over a short Celite bed, concentrated under reduced pressure, and purified by silica column eluting with (EA/hexane = 8:92) to afford (*Z*)-4-phenyl-3-((*E*)-phenyl­(phenylimino)­methyl)-4-(phenylamino)­but-3-en-2-one **3a** (94 mg, 0.22 mmol, 64%) as a yellow oil.

The compound **5a** was synthesized using the same catalytic procedure.

### Procedure for the Gram-Scale Synthesis of (*Z*)-4-Phenyl-3-((*E*)-phenyl­(phenylimino)­methyl)-4-(phenylamino)­but-3-en-2-one (**3a**)

A 100 mL flask was charged with JohnPhos gold chloride (373 mg, 0.70 mmol, 0.1 equiv) and AgSbF_6_ (242 mg, 0.70 mmol, 0.1 equiv), and to this mixture was added wet 1,2-dichloroethane (5 mL). The resulting mixture was stirred at room temperature for 5 min. To this mixture was added dropwise a wet 1,2-dichloroethane (20 mL) solution of (3-methylbut-3-en-1-yn-1-yl)­benzene **1a** (1.0 g, 7.03 mmol, 1.0 equiv) and (*Z*)-*N*,1-diphenylmethanimine oxide **2a** (4.16 g, 21.1 mmol, 3.0 equiv) at room temperature. After addition, the reaction mixture was stirred at 80 °C in an oil bath for 19 h. The reaction mixture was filtered over a short Celite bed, concentrated under reduced pressure, and purified by silica column eluting with (EA/hexane = 8:92) to afford (*Z*)-4-phenyl-3-((*E*)-phenyl­(phenylimino)­methyl)-4-(phenylamino)­but-3-en-2-one **3a** (1.78 g, 4.27 mmol, 61%) as a yellow oil.

#### (*Z*)-4-Phenyl-3-((*E*)-phenyl­(phenylimino)­methyl)-4-(phenylamino)­but-3-en-2-one (**3a**)

Purified by silica gel column, ethyl acetate/hexane (8:92) as eluent; yellow oil (94 mg, 0.22 mmol, 64%); ^1^H NMR (700 MHz, CDCl_3_): δ 13.51 (s, 1H), 8.02 (d, *J* = 7.9 Hz, 2H), 7.44–7.41 (m, 3H), 7.17–7.13 (m, 3H), 6.99 (t, *J* = 7.0 Hz, 4H), 6.95 (s, 2H), 6.89 (t, *J* = 7.4 Hz, 1H), 6.70 (s, 1H), 6.56 (d, *J* = 7.9 Hz, 2H), 6.50 (d, *J* = 7.5 Hz, 2H), 2.04 (s, 3H); ^13^C­{^1^H} NMR (175 MHz, CDCl_3_): δ 196.5, 165.3, 159.9, 149.7, 142.1, 138.9, 132.9, 130.3, 129.3, 128.54, 128.53, 128.52, 128.2, 127.9, 124.2, 123.9, 123.5, 121.7, 106.9, 29.5, one carbon merged with other peaks; HRMS (ESI-TOF) *m*/*z*: [M – H] calcd for C_29_H_23_N_2_O: 415.1810; found: 415.1813.

#### (*Z*)-3-((*E*)-Phenyl­(phenylimino)­methyl)-4-(phenylamino)-4-(*p*-tolyl)­but-3-en-2-one (**3b**)

Purified by silica gel column, ethyl acetate/hexane (9:91) as eluent; yellow oil (92 mg, 0.21 mmol, 67%); ^1^H NMR for major isomer (700 MHz, CDCl_3_): δ 13.49 (s, 1H), 7.95 (d, *J* = 8.1 Hz, 2H), 7.26 (d, *J* = 7.5 Hz, 3H), 7.02–6.97 (m, 8H), 6.90 (q, *J* = 15.1 and 7.5 Hz, 2H), 6.56 (d, *J* = 7.9 Hz, 2H), 6.44 (d, *J* = 7.4 Hz, 2H), 2.43 (s, 3H), 2.06 (s, 3H); ^1^H NMR for minor isomer: δ 13.51 (s, 1H), 8.04 (d, *J* = 7.9 Hz, 2H), 7.45 (t, *J* = 5.1 Hz, 3H), 7.18–7.14 (m, 8H), 6.77 (d, *J* = 7.5 Hz, 2H), 6.58 (d, *J* = 7.9 Hz, 2H), 6.50 (d, *J* = 7.4 Hz, 2H), 2.21 (s, 3H), 2.04 (s, 3H), rest of the peaks merged with other peaks; ^13^C­{^1^H} NMR for major isomer (175 MHz, CDCl_3_): δ 196.7, 165.5, 159.7, 149.84, 140.7, 139.45, 139.0, 132.9, 130.3, 129.3, 128.593, 128.57, 128.49, 128.1, 127.8, 124.18, 123.7, 123.4, 121.6, 107.0, 29.5, 21.4; ^13^C­{^1^H} NMR for minor isomer: 196.3, 165.0, 160.2, 149.85, 142.1, 139.47, 139.1, 130.0, 129.2, 128.591, 128.51, 128.50, 128.2, 124.14, 123.8, 123.4, 121.6, 106.9, 29.5, 21.3, rest of the peaks merged with other peaks; HRMS (FD) *m*/*z*: [M]^+^ calcd for C_30_H_26_N_2_O: 430.2039; found: 430.2029.

#### (*Z*)-4-(4-Methoxyphenyl)-3-((*E*)-phenyl­(phenylimino)­methyl)-4-(phenylamino)­but-3-en-2-one (**3c**)

Purified by silica gel column, ethyl acetate/hexane (10:90) as eluent; yellow oil (91 mg, 0.20 mmol, 70%); ^1^H NMR for major isomer (400 MHz, CDCl_3_): δ 13.47 (s, 1H), 7.99 (d, *J* = 8.8 Hz, 2H), 7.17–7.11 (m, 5H), 6.97–6.94 (m, 5H), 6.90–6.85 (m, 2H), 6.53 (d, *J* = 7.9 Hz, 2H), 6.49–6.46 (m, 3H), 3.87 (s, 3H), 2.06 (s, 3H); ^1^H NMR for minor isomer: δ 13.46 (s, 1H), 8.03 (d, *J* = 7.6 Hz, 2H), 7.45–7.42 (m, 4H), 7.03–6.98 (m, 8H), 6.56 (d, *J* = 8.0 Hz, 2H), 6.41 (d, *J* = 8.2 Hz, 3H), 3.68 (s, 3H), 2.01 (s, 3H), rest of the peaks merged with other peaks; ^13^C­{^1^H} NMR for major isomer (175 MHz, CDCl_3_): δ 196.6, 164.4, 161.5, 159.6, 149.9, 142.1, 139.0, 134.9, 130.2, 129.3, 128.55, 128.4, 128.15, 125.2, 124.17, 123.6, 123.45, 121.7, 113.8, 107.0, 55.3, 29.51; ^13^C­{^1^H} NMR for minor isomer: 196.2, 165.6, 160.3, 159.9, 149.8, 139.2, 132.9, 130.3, 128.57, 128.19, 127.8, 124.10, 123.8, 123.46, 121.5, 113.3, 106.9, 55.1, 29.57, rest of the peaks merged with other peaks; HRMS (FD) *m*/*z*: [M]^+^ calcd for C_30_H_26_N_2_O_2_: 446.1988; found: 446.1985.

#### (*Z*)-4-(4-Chlorophenyl)-3-((*E*)-phenyl­(phenylimino)­methyl)-4-(phenylamino)­but-3-en-2-one (**3d**)

Purified by silica gel column, ethyl acetate/hexane (8:92) as eluent; yellow oil (82 mg, 0.18 mmol, 64%); ^1^H NMR for major isomer (700 MHz, CDCl_3_): δ 13.51 (s, 1H), 7.95 (d, *J* = 8.4 Hz, 2H), 7.47–7.44 (m, 2H), 7.39 (d, *J* = 8.4 Hz, 2H), 7.03–6.98 (m, 6H), 6.69 (s, 1H), 6.55 (t, *J* = 6.7 Hz, 4H), 6.52 (d, *J* = 7.9 Hz, 2H), 2.02 (s, 3H); ^1^H NMR for minor isomer: δ 13.41 (s, 1H), 8.03 (d, *J* = 7.7 Hz, 2H), 7.18–7.13 (m, 8H), 6.95–6.88 (m, 9H), 2.04 (s, 3H), rest of the peaks merged with other peaks; ^13^C­{^1^H} NMR for major isomer (175 MHz, CDCl_3_): δ 196.2, 164.0, 160.1, 149.4, 140.6, 138.8, 136.4, 132.8, 130.6, 129.8, 128.72, 128.71, 128.56, 128.32, 127.9, 124.4, 124.2, 123.5, 121.8, 106.4, 29.4; ^13^C­{^1^H} NMR for minor isomer: 196.8, 164.9, 158.5, 149.5, 141.9, 138.6, 135.4, 131.4, 129.4, 128.70, 128.51, 128.33, 128.2, 124.5, 124.1, 123.6, 121.7, 107.0, 29.6, rest of the peaks merged with other peaks; HRMS (FD) *m*/*z*: [M]^+^ calcd for C_29_H_23_N_2_OCl: 450.1493; found: 450.1483.

#### (*Z*)-4-(4-Bromophenyl)-3-((*E*)-phenyl­(phenylimino)­methyl)-4-(phenylamino)­but-3-en-2-one (**3e**)

Purified by silica gel column, ethyl acetate/hexane (8:92) as eluent; yellow oil (69 mg, 0.13 mmol, 61%); ^1^H NMR for major isomer (500 MHz, CDCl_3_): δ 13.51 (s, 1H), 7.88 (d, *J* = 8.3 Hz, 2H), 7.54 (d, *J* = 8.4 Hz, 2H), 7.46 (s, 2H), 7.07 (s, 1H), 7.00 (q, *J* = 15.4 and 7.9 Hz, 6H), 6.55 (t, *J* = 6.1 Hz, 4H), 6.52 (d, *J* = 8.0 Hz, 2H), 2.02 (s, 3H); ^1^H NMR for minor isomer: δ 13.40 (s, 1H), 8.03 (s, 2H), 7.17 (t, *J* = 7.6 Hz, 8H), 6.91 (t, *J* = 5.5 Hz, 7H), 6.48 (s, 2H), 2.03 (s, 3H), rest of the peaks merged with other peaks; ^13^C­{^1^H} NMR for major isomer (125 MHz, CDCl_3_): δ 196.2, 164.1, 160.2, 149.4, 141.1, 138.8, 132.8, 131.7, 130.0, 129.4, 128.74, 128.58, 128.33, 127.9, 124.5, 124.3, 123.7, 123.5, 121.8, 106.3, 29.4; ^13^C­{^1^H} NMR for minor isomer: 196.9, 164.9, 158.5, 149.5, 141.9, 138.6, 131.9, 131.1, 130.6, 128.71, 128.52, 128.34, 125.0, 124.4, 124.1, 123.8, 107.0, 29.6, rest of the peaks merged with other peaks; HRMS (FD) *m*/*z*: [M]^+^ calcd for C_29_H_23_N_2_OBr: 494.0988; found: 494.0978.

#### (*Z*)-3-((*E*)-Phenyl­(phenylimino)­methyl)-4-(phenylamino)-4-(*m*-tolyl)­but-3-en-2-one (**3f**)

Purified by silica gel column, ethyl acetate/hexane (8:92) as eluent; yellow oil (90 mg, 0.20 mmol, 65%); ^1^H NMR for major isomer (400 MHz, CDCl_3_): δ 13.50 (s, 1H), 8.01 (s, 1H), 7.83 (s, 1H), 7.43–7.39 (m, 3H), 7.33 (t, *J* = 7.5 Hz, 1H), 6.94 (d, *J* = 8.0 Hz, 3H), 6.88 (t, *J* = 7.0 Hz, 3H), 6.70 (s, 1H), 6.56 (d, *J* = 7.9 Hz, 4H), 6.46 (d, *J* = 7.9 Hz, 2H), 2.40 (s, 3H), 2.04 (s, 3H); ^1^H NMR for minor isomer: δ 13.52 (s, 1H), 7.99 (s, 1H), 7.80 (s, 1H), 7.25 (t, *J* = 7.4 Hz, 2H), 7.20–7.15 (m, 6H), 7.01–6.93 (m, 7H), 6.51 (d, *J* = 7.7 Hz, 2H), 2.05 (s, 3H), 1.96 (s, 3H), rest of the peaks merged with other peaks; ^13^C­{^1^H} NMR for major isomer (125 MHz, CDCl_3_): δ 196.6, 165.5, 159.8, 149.81, 142.14, 139.0, 138.2, 132.9, 131.2, 129.9, 129.2, 128.8, 128.50, 128.48, 128.2, 127.88, 126.0, 124.1, 123.4, 123.2, 121.6, 107.1, 29.6, 21.1; ^13^C­{^1^H} NMR for minor isomer: 196.3, 165.3, 160.1, 149.83, 142.18, 137.4, 132.8, 130.2, 128.52, 128.45, 128.3, 128.1, 127.80, 124.2, 123.8, 121.7, 106.9, 29.5, 21.1, rest of the peaks merged with other peaks; HRMS (FD) *m*/*z*: [M]^+^ calcd for C_30_H_26_N_2_O: 430.2039; found: 430.2035.

#### (*Z*)-4-(3-Chlorophenyl)-3-((*E*)-phenyl­(phenylimino)­methyl)-4-(phenylamino)­but-3-en-2-one (**3g**)

Purified by silica gel column, ethyl acetate/hexane (8:92) as eluent; yellow oil (73 mg, 0.16 mmol, 57%); ^1^H NMR for major isomer (500 MHz, CDCl_3_): δ 13.55 (s, 1H), 8.04 (s, 1H), 7.99 (s, 1H), 7.48 (s, 1H), 7.36 (t, *J* = 7.7 Hz, 1H), 7.26–7.19 (m, 4H), 7.06–7.01 (m, 6H), 6.59 (d, *J* = 7.7 Hz, 5H), 2.04 (s, 3H); ^1^H NMR for minor isomer: δ 13.42 (s, 1H), 7.88 (s, 1H), 7.86 (s, 1H), 7.48 (s, 1H), 7.40 (s, 2H), 7.17 (d, *J* = 7.5 Hz, 3H), 6.98 (s, 6H), 6.93 (d, *J* = 7.9 Hz, 2H), 6.52 (s, 3H), 2.08 (s, 3H), rest of the peaks merged with other peaks; ^13^C­{^1^H} NMR for major isomer (100 MHz, CDCl_3_): δ 196.1, 163.9, 160.3, 149.3, 144.1, 138.7, 134.7, 132.8, 130.2, 129.6, 129.4, 128.7, 128.6, 128.5, 128.3, 127.9, 126.7, 124.4, 123.5, 121.8, 106.4, 29.4, one carbon merged with other peaks; ^13^C­{^1^H} NMR for minor isomer: 196.9, 164.7, 158.0, 149.5, 141.8, 138.5, 133.7, 130.5, 129.2, 128.4, 128.2, 124.6, 124.1, 121.6, 107.1, 29.5, rest of the peaks merged with other peaks; HRMS (FD) *m*/*z*: [M]^+^ calcd for C_29_H_23_N_2_OCl: 450.1493; found: 450.1484.

#### (*Z*)-4-(Naphthalen-2-yl)-3-((*Z*)-phenyl­(phenylimino)­methyl)-4-(phenylamino)­but-3-en-2-one (**3h**)

Purified by silica gel column, ethyl acetate/hexane (9:91) as eluent; yellow oil (79 mg, 0.16 mmol, 65%); ^1^H NMR for major isomer (700 MHz, CDCl_3_): δ 13.57 (s, 1H), 8.48 (s, 1H), 8.04 (s, 1H), 7.89 (d, *J* = 8.4 Hz, 2H), 7.56–7.52 (m, 2H), 7.44–7.41 (m, 3H), 7.18 (d, *J* = 7.7 Hz, 2H), 7.01 (d, *J* = 7.4 Hz, 2H), 6.94–6.91 (m, 3H), 6.58 (d, *J* = 7.7 Hz, 4H), 6.50 (d, *J* = 7.4 Hz, 2H), 2.09 (s, 3H); ^1^H NMR for minor isomer: δ 13.57 (s, 1H), 8.22 (s, 1H), 8.21 (s, 1H), 7.97 (d, *J* = 7.6 Hz, 2H), 7.67 (d, *J* = 8.1 Hz, 1H), 7.34 (t, *J* = 7.0 Hz, 3H), 7.12 (q, *J* = 14.4 and 7.3 Hz, 4H), 6.99 (s, 2H), 6.89 (d, *J* = 7.3 Hz, 4H), 6.83 (t, *J* = 7.3 Hz, 2H), 6.34 (d, *J* = 7.6 Hz, 2H), 2.09 (s, 3H), rest of the peaks merged with other peaks; ^13^C­{^1^H} NMR for major isomer (175 MHz, CDCl_3_): δ 196.7, 165.1, 160.0, 149.7, 139.7, 138.99, 134.5, 133.13, 132.9, 130.43, 129.3, 129.1, 128.54, 128.51, 128.3, 128.2, 127.8, 127.2, 127.0, 126.3, 125.2, 124.28, 124.0, 123.48, 121.7, 106.8, 29.6; ^13^C­{^1^H} NMR for minor isomer: 196.6, 165.3, 159.8, 149.8, 142.0, 138.96, 133.17, 132.2, 130.46, 129.4, 128.6, 128.56, 128.1, 127.7, 127.5, 126.1, 124.25, 123.9, 123.41, 121.5, 107.2, 29.5, rest of the peaks merged with other peaks; HRMS (FD) *m*/*z*: [M]^+^ calcd for C_33_H_26_N_2_O: 466.2039; found: 466.2035.

#### (*Z*)-1-Phenyl-2-((*E*)-phenyl­(phenylimino)­methyl)-1-(phenylamino)­pent-1-en-3-one (**3i**)

Purified by silica gel column, ethyl acetate/hexane (7:93) as eluent; yellow oil (95 mg, 0.22 mmol, 69%); ^1^H NMR (400 MHz, CDCl_3_): δ 13.53 (s, 1H), 8.02–8.00 (m, 2H), 7.43–7.39 (m, 3H), 7.17–7.11 (m, 3H), 7.00–6.92 (m, 5H), 6.87 (t, *J* = 7.3 Hz, 1H), 6.69 (s, 2H), 6.54 (d, *J* = 7.6 Hz, 2H), 6.47 (d, *J* = 7.4 Hz, 2H), 2.40–2.32 (m, 1H), 2.31–2.23 (m, 1H), 0.99 (t, *J* = 7.3 Hz, 3H); ^13^C­{^1^H} NMR (175 MHz, CDCl_3_): δ 199.7, 165.1, 159.4, 149.7, 142.2, 139.1, 133.1, 130.3, 129.2, 128.54, 128.50, 128.2, 127.2, 127.8, 124.09, 124.02, 123.3, 121.8, 106.7, 34.1, 8.8, two carbons are merged with other peaks; HRMS (ESI-TOF) *m*/*z*: [M + H] calcd for C_30_H_27_N_2_O: 431.2123; found: 431.2120.

#### (*Z*)-3-((*E*)-(4-Chlorophenyl)­(phenylimino)­methyl)-4-phenyl-4-(phenylamino)­but-3-en-2-one (**4a**)

Purified by silica gel column, ethyl acetate/hexane (8:92) as eluent; yellow oil (102 mg, 0.22 mmol, 64%); ^1^H NMR for major isomer (400 MHz, CDCl_3_): δ 13.51 (s, 1H), 7.94 (d, *J* = 8.4 Hz, 2H), 7.38 (d, *J* = 8.4 Hz, 2H), 7.15–7.10 (m, 3H), 6.97–6.87 (m, 7H), 6.54–6.48 (m, 5H), 2.02 (s, 3H); ^1^H NMR for minor isomer: δ 13.41 (s, 1H), 8.02 (d, *J* = 7.5 Hz, 2H), 7.45 (d, *J* = 5.3 Hz, 2H), 7.18 (d, *J* = 7.8 Hz, 3H), 7.04–6.99 (m, 7H), 6.57–6.55 (m, 5H), 2.04 (s, 3H), rest of the peaks merged with other peaks; ^13^C­{^1^H} NMR for major isomer (100 MHz, CDCl_3_): δ 196.2, 164.0, 160.1, 149.4, 140.6, 138.8, 136.4, 132.8, 130.6, 129.8, 128.7, 128.56, 128.3, 128.2, 127.9, 124.4, 124.2, 123.5, 121.8, 106.4, 29.4; ^13^C­{^1^H} NMR for minor isomer: 196.8, 164.9, 158.5, 149.5, 141.9, 138.7, 135.4, 131.4, 130.1, 129.4, 128.51, 124.5, 124.1, 123.7, 107.1, 29.6, rest of the peaks merged with other peaks; HRMS (ESI-TOF) *m*/*z*: [M + H] calcd for C_29_H_24_ClN_2_O: 451.1577; found: 451.1571.

#### (*Z*)-4-Phenyl-4-(phenylamino)-3-((*E*)-(phenylimino)­(*p*-tolyl)­methyl)­but-3-en-2-one (**4b**)

Purified by silica gel column, ethyl acetate/hexane (9:91) as eluent; yellow oil (88 mg, 0.20 mmol, 58%); ^1^H NMR for major isomer (700 MHz, CDCl_3_): δ 13.47 (s, 1H), 7.93 (d, *J* = 7.9 Hz, 2H), 7.43 (s, 2H), 7.24 (s, 1H), 7.14 (t, *J* = 6.7 Hz, 3H), 6.98–6.95 (m, 4H), 6.89 (q, *J* = 15.6 and 7.4 Hz, 2H), 6.75 (s, 1H), 6.54 (d, *J* = 8.0 Hz, 2H), 6.43 (d, *J* = 7.9 Hz, 2H), 2.42 (s, 3H), 2.05 (s, 3H); ^1^H NMR for minor isomer: δ 13.49 (s, 1H), 8.02 (d, *J* = 7.8 Hz, 2H), 7.42 (s, 2H), 7.25 (s, 1H), 7.17 (d, *J* = 7.8 Hz, 3H), 7.00 (t, *J* = 7.8 Hz, 4H), 6.76 (s, 1H), 6.57 (d, *J* = 8.0 Hz, 2H), 6.49 (d, *J* = 8.0 Hz, 2H), 2.20 (s, 3H), 2.02 (s, 3H), rest of the peaks merged with other peaks; ^13^C­{^1^H} NMR for major isomer (175 MHz, CDCl_3_): δ 196.3, 165.5, 159.7, 149.86, 142.1, 139.48, 139.0, 132.9, 130.3, 129.32, 128.6, 128.58, 128.1, 127.8, 124.19, 123.9, 123.49, 121.69, 107.0, 29.57, 21.4, one carbon merged with other peaks; ^13^C­{^1^H} NMR for minor isomer: 196.7, 165.1, 160.2, 149.85, 140.7, 139.46, 139.1, 130.0, 129.30, 128.59, 128.51, 128.50, 128.2, 124.15, 123.7, 123.46, 121.66, 106.9, 29.54, 21.3, rest of the peaks merged with other peaks; HRMS (FD) *m*/*z*: [M]^+^ calcd for C_30_H_26_N_2_O: 430.2039; found: 430.2040.

#### (*Z*)-3-((*E*)-(3-Chlorophenyl)­(phenylimino)­methyl)-4-phenyl-4-(phenylamino)­but-3-en-2-one (**4c**)

Purified by silica gel column, ethyl acetate/hexane (8:92) as eluent; yellow oil (99 mg, 0.22 mmol, 62%); ^1^H NMR for major isomer (500 MHz, CDCl_3_): δ 13.57 (s, 1H), 8.06 (s, 1H), 8.01 (s, 1H), 7.43 (d, *J* = 8.0 Hz, 1H), 7.38 (t, *J* = 7.7 Hz, 1H), 7.18 (t, *J* = 7.5 Hz, 3H), 7.08–7.03 (m, 5H), 6.95 (d, *J* = 7.7 Hz, 2H), 6.61 (d, *J* = 7.3 Hz, 5H), 2.07 (s, 3H); ^1^H NMR for minor isomer: δ 13.44 (s, 1H), 7.90 (s, 1H), 7.88 (s, 1H), 7.50 (s, 3H), 7.28–7.22 (m, 5H), 7.00 (s, 6H), 6.54 (s, 3H), 2.10 (s, 3H), rest of the peaks merged with other peaks; ^13^C­{^1^H} NMR for major isomer (100 MHz, CDCl_3_): δ 196.1, 164.0, 160.3, 149.3, 144.1, 138.7, 134.7, 132.8, 130.2, 129.6, 129.4, 128.7, 128.6, 128.5, 128.3, 128.0, 126.7, 124.4, 123.5, 121.8, 106.4, 29.4, one carbon merged with other peaks; ^13^C­{^1^H} NMR for minor isomer: 196.9, 164.8, 158.0, 149.5, 141.8, 138.5, 133.7, 130.5, 129.2, 128.4, 128.2, 124.6, 124.1, 121.6, 107.1, 29.5, rest of the peak merged with other peaks; HRMS (FD) *m*/*z*: [M]^+^ calcd for C_29_H_23_ClN_2_O: 450.1486; found: 450.1493.

#### (*Z*)-4-Phenyl-4-(phenylamino)-3-((*E*)-(phenylimino)­(*m*-tolyl)­methyl)­but-3-en-2-one (**4d**)

Purified by silica gel column, ethyl acetate/hexane (8:92) as eluent; yellow oil (83 mg, 0.19 mmol, 55%); ^1^H NMR for major isomer (400 MHz, CDCl_3_): δ 13.49 (s, 1H), 8.00 (s, 1H), 7.82 (s, 1H), 7.43 (d, *J* = 5.3 Hz, 2H), 7.32 (t, *J* = 7.5 Hz, 1H), 7.17 (t, *J* = 7.8 Hz, 2H), 6.99 (t, *J* = 7.6 Hz, 5H), 6.65 (s, 1H), 6.55 (d, *J* = 8.0 Hz, 4H), 6.46 (d, *J* = 7.2 Hz, 2H), 2.40 (s, 3H), 2.03 (s, 3H); ^1^H NMR for minor isomer: δ 13.51 (s, 1H), 7.98 (s, 1H), 7.79 (s, 1H), 7.41 (d, *J* = 4.3 Hz, 1H), 7.14 (t, *J* = 7.5 Hz, 4H), 6.94 (d, *J* = 7.9 Hz, 6H), 6.88 (t, *J* = 7.3 Hz, 3H), 6.51 (d, *J* = 7.1 Hz, 2H), 2.04 (s, 3H), 1.95 (s, 3H), rest of the peaks merged with other peaks; ^13^C­{^1^H} NMR for major isomer (125 MHz, CDCl_3_): δ 196.7, 165.5, 159.8, 149.8, 142.1, 139.0, 137.4, 133.0, 131.2, 129.9, 129.3, 128.51, 128.49, 128.2, 127.9, 126.0, 124.2, 123.8, 123.5, 121.71, 107.1, 29.6, 21.5, one carbon merged with other peaks; ^13^C­{^1^H} NMR for minor isomer: 196.4, 165.3, 160.1, 142.2, 138.2, 132.8, 130.2, 128.8, 128.54, 128.46, 128.3, 128.1, 127.8, 124.1, 123.3, 121.70, 106.9, 30.9, 21.1, rest of the peaks merged with other peaks; HRMS (ESI-TOF) *m*/*z*: [M + H]^+^ calcd for C_30_H_27_N_2_O: 431.2123; found: 431.2126.

#### (*Z*)-4-((4-Chlorophenyl)­amino)-3-((*E*)-((4-chlorophenyl)­imino)­(phenyl)­methyl)-4-phenylbut-3-en-2-one (**4e**)

Purified by silica gel column, ethyl acetate/hexane (9:91) as eluent; pale yellow solid (106 mg, 0.21 mmol, 62%); ^1^H NMR (500 MHz, CDCl_3_): δ 13.46 (s, 1H), 8.01 (d, *J* = 6.7 Hz, 2H), 7.45 (d, *J =* 7.5 Hz, 3H), 7.17 (d, *J* = 7.4 Hz, 1H), 7.11 (d, *J* = 8.5 Hz, 2H), 6.98 (d, *J* = 5.8 Hz, 5H), 6.95 (s, 1H), 6.48 (d, *J* = 8.7 Hz, 2H), 6.34 (d, *J* = 8.5 Hz, 2H), 2.02 (s, 3H); ^13^C­{^1^H} NMR (100 MHz, CDCl_3_): δ 196.8, 165.7, 159.5, 148.2, 141.7, 137.5, 132.5, 130.7, 129.8, 129.6, 129.2, 128.7, 128.5, 128.3, 128.2, 124.6, 122.9, 107.0, 29.6, two carbons merged with other peaks; HRMS (ESI-TOF) *m*/*z*: [M + Na]^+^ calcd for C_29_H_22_Cl_2_N_2_ONa: 507.1006; found: 507.1003.

#### (*Z*)-4-((4-Bromophenyl)­amino)-3-((*E*)-((4-bromophenyl)­imino)­(phenyl)­methyl)-4-phenylbut-3-en-2-one (**4f**)

Purified by silica gel column, ethyl acetate/hexane (8:92) as eluent; yellow oil (115 mg, 0.20 mmol, 57%); ^1^H NMR (500 MHz, CDCl_3_): δ 13.49 (s, 1H), 8.05 (d, *J* = 7.4 Hz, 2H), 7.50 (d, *J =* 7.5 Hz, 3H), 7.31 (s, 1H), 7.29 (d, *J* = 3.8 Hz, 3H), 7.23 (t, *J* = 7.0 Hz, 1H), 7.16 (d, *J* = 8.2 Hz, 2H), 7.04 (s, 3H), 6.47 (d, *J* = 8.4 Hz, 2H), 6.32 (d, *J* = 8.0 Hz, 2H), 2.06 (s, 3H); ^13^C­{^1^H} NMR (125 MHz, CDCl_3_): δ 196.8, 165.6, 159.3, 148.7, 141.6, 138.0, 132.5, 131.6, 131.2, 130.7, 129.7, 128.7, 128.5, 128.2, 124.9, 123.2, 117.5, 117.0, 107.1, 29.6, one carbon merged with other peaks; HRMS (FD) *m*/*z*: [M]^+^ calcd for C_29_H_22_N_2_OBr_2_: 572.0093; found: 572.0089.

#### (*Z*)-4-Phenyl-3-((*E*)-phenyl­(*p*-tolylimino)­methyl)-4-(*p*-tolylamino)­but-3-en-2-one (**4g**)

Purified by silica gel column, ethyl acetate/hexane (9:91) as eluent; yellow oil (109 mg, 0.22 mmol, 64%); ^1^H NMR (500 MHz, CDCl_3_): δ 13.50 (s, 1H), 7.94 (d, *J* = 7.6 Hz, 2H), 7.38 (s, 4H), 7.11 (s, 1H), 6.99–6.91 (m, 5H), 6.79 (d, *J* = 8.1 Hz, 2H), 6.50 (d, *J* = 8.0 Hz, 2H), 6.46 (d, *J* = 8.1 Hz, 2H), 2.28 (s, 3H), 2.14 (s, 3H), 2.00 (s, 3H); ^13^C­{^1^H} NMR (100 MHz, CDCl_3_): δ 196.2, 164.9, 160.1, 147.3, 142.4, 136.4, 133.9, 133.6, 133.1, 130.0, 129.1, 128.9, 128.6, 128.4, 128.3, 127.8, 123.3, 121.9, 106.9, 29.3, 20.9, 20.6, one carbon merged with other peaks; HRMS (FD) *m*/*z*: [M]^+^ calcd for C_31_H_28_N_2_O: 444.2196; found: 444.2188.

#### (*Z*)-4-((3-Chlorophenyl)­amino)-3-((*E*)-((3-chlorophenyl)­imino)­(phenyl)­methyl)-4-phenylbut-3-en-2-one (**4h**)

Purified by silica gel column, ethyl acetate/hexane (9:91) as eluent; yellow solid (115 mg, 0.23 mmol, 67%); ^1^H NMR (500 MHz, CDCl_3_): δ 13.45 (s, 1H), 8.07 (d, *J* = 7.0 Hz, 2H), 7.50 (d, *J =* 7.5 Hz, 3H), 7.26 (t, *J* = 5.6 Hz, 1H), 7.14–7.06 (m, 4H), 6.98–6.88 (m, 4H), 6.58 (s, 1H), 6.43 (d, *J* = 7.8 Hz, 1H), 6.38 (d, *J* = 7.9 Hz, 1H), 6.11 (s, 1H), 2.07 (s, 3H); ^13^C­{^1^H} NMR (125 MHz, CDCl_3_): δ 196.9, 166.1, 159.3, 151.0, 141.5, 140.2, 134.1, 133.8, 132.4, 130.9, 129.8, 129.4, 129.1, 128.7, 128.6, 128.2, 124.3, 123.7, 123.4, 122.4, 121.6, 118.0, 107.3, 29.7, one carbon merged with other peaks; HRMS (FD) *m*/*z*: [M]^+^ calcd for C_29_H_22_N_2_OCl_2_: 484.1103; found: 484.1102.

#### (*Z*)-4-((3-Bromophenyl)­amino)-3-((*E*)-((3-bromophenyl)­imino)­(phenyl)­methyl)-4-phenylbut-3-en-2-one (**4i**)

Purified by silica gel column, ethyl acetate/hexane (8:92) as eluent; yellow oil (104 mg, 0.21 mmol, 61%); ^1^H NMR (400 MHz, CDCl_3_): δ 13.42 (s, 1H), 8.04 (d, *J* = 7.6 Hz, 2H), 7.50–7.45 (m, 3H), 7.26–7.22 (m, 2H), 7.12–7.00 (m, 5H), 6.84 (t, *J =* 8.1 Hz, 1H), 6.72 (s, 1H), 6.45–6.39 (m, 3H), 6.23 (s, 1H), 2.04 (s, 3H); ^13^C­{^1^H} NMR (100 MHz, CDCl_3_): δ 196.9, 166.1, 159.3, 151.2, 141.5, 140.4, 132.4, 130.9, 129.9, 129.7, 129.4, 128.79. 128.72, 128.3, 127.2, 126.6, 126.3, 125.3, 122.1, 122.0, 121.9, 118.4, 107.3, 29.7, one carbon merged with other peaks; HRMS (FD) *m*/*z*: [M]^+^ calcd for C_29_H_22_N_2_OBr_2_: 572.0093; found: 572.0091.

#### (*Z*)-4-Phenyl-3-((*E*)-phenyl­(*m*-tolylimino)­methyl)-4-(*m*-tolylamino)­but-3-en-2-one (**4j**)

Purified by silica gel column, ethyl acetate/hexane (8:92) as eluent; yellow oil (108 mg, 0.22 mmol, 63%); ^1^H NMR (500 MHz, CDCl_3_): δ 13.47 (s, 1H), 8.01 (d, *J* = 7.8 Hz, 2H), 7.43 (d, *J =* 7.0 Hz, 3H), 7.16–7.05 (m, 2H), 6.95 (s, 2H), 6.85–6.78 (m, 3H), 6.69 (d, *J =* 7.4 Hz, 2H), 6.42 (s, 1H), 6.38 (d, *J* = 7.8 Hz, 1H), 6.27 (d, *J =* 8.0 Hz, 1H), 6.11 (s, 1H), 2.18 (s, 3H), 2.08 (s, 3H), 2.02 (s, 3H); ^13^C­{^1^H} NMR (125 MHz, CDCl_3_): δ 196.4, 165.2, 159.8, 149.8, 142.2, 138.9, 138.4, 137.7, 133.1, 130.3, 129.1, 128.55, 128.53, 128.2, 127.9, 127.7, 124.9, 124.5, 124.1, 123.2, 120.5, 117.4, 107.0, 29.5, 21.4, 21.1, one carbon merged with other peaks; HRMS (FD) *m*/*z*: [M]^+^ calcd for C_31_H_28_N_2_O: 444.2196; found: 444.2202.

#### (1,5-Diphenyl-3-(phenylamino)-1*H*-pyrrol-2-yl)­(phenyl)­methanone (**5a**)

Purified by silica gel column, ethyl acetate/hexane (10:90) as eluent; yellow solid (99 mg, 0.23 mmol, 69%); ^1^H NMR (500 MHz, CDCl_3_): δ 9.65 (s, 1H), 7.36–7.31 (m, 4H), 7.26 (d, *J =* 7.2 Hz, 2H), 7.23–7.18 (m, 3H), 7.09 (t, *J* = 7.2 Hz, 3H), 7.00 (t, *J* = 7.5 Hz, 3H), 6.93–6.88 (m, 3H), 6.83 (t, *J* = 5.7 Hz, 2H), 6.61 (s, 1H); ^13^C­{^1^H} NMR (175 MHz, CDCl_3_): δ 185.9, 143.6, 142.6, 142.1, 140.1, 138.2, 131.8, 129.5, 129.2, 128.6, 128.08, 128.02, 127.9, 127.2, 126.7, 121.5, 121.4, 118.4, 99.6 (d), two carbons merged with other peaks; HRMS (FD) *m*/*z*: [M]^+^ calcd for C_29_H_22_N_2_O: 414.1726; found: 414.1728.

### Control Experiment Procedures (for Scheme 4, Equations 6–8 from Main Text)

The reactions were carried out under the same conditions as those described in the section on the Standard Catalytic Reaction Procedure.

### Mechanistic Investigation: Investigation for the Role of *N*-(Phenyl-*d*_5_)­hydroxylamine (***d***_**5**_ **-2l**)

#### Procedure for the Synthesis of Compound ***d***_**5**_ **-3a/** ***d***_**0**_ **-3a**

The reaction was carried out under the same conditions as those described in the section on the Standard Catalytic Reaction Procedure. We have attached the HRMS (FD) data for compound ***d***_**5**_ **-3a/** ***d***_**0**_ **-3a** (see Supporting Information).

##### (*Z*)-4-Phenyl-3-((*E*)-phenyl­(phenylimino)­methyl)-4-((phenyl-*d*_5_)­amino)­but-3-en-2-one and (*Z*)-4-Phenyl-3-((*E*)-phenyl­(phenylimino)­methyl)-4-(phenylamino)­but-3-en-2-one (***d***_**5**_ **-3a/*d***_**0**_ **-3a**)

Purified by silica gel column, ethyl acetate/hexane (7:93) as eluent; yellow oil (67 mg, 0.16 mmol, 46%); ^1^H NMR (700 MHz, CDCl_3_): δ 13.51 (s, 1H), 8.01 (d, *J* = 7.1 Hz, 2H), 7.43 (d, *J =* 7.1 Hz, 3H), 7.17–7.12 (m, 3H), 6.99 (t, *J =* 7.6 Hz, 2.9H), 6.94 (s, 2H), 6.88 (t, *J =* 7.2 Hz, 1H), 6.72 (s, 1H), 6.55 (d, *J =* 7.8 Hz, 1.5H), 6.50 (s, 1.2H), 2.03 (s, 3H); ^13^C­{^1^H} NMR (100 MHz, CDCl_3_): δ 196.5, 165.4, 160.0, 149.6, 142.0, 138.9, 132.9, 130.4, 129.3, 128.5, 128.2, 127.9, 124.3, 124.1, 123.5, 121.8, 106.9, 29.5; HRMS (FD) *m*/*z*: [M]^+^ calcd for C_29_H_19_D_5_N_2_O: 421.2169; found: 421.2167.

### Synthetic Procedure for Chemical Functionalization of (**3a**)

#### Procedure for the Synthesis of (3*S*,4*S*)-3-Benzyl-4-phenyl-4-(phenylamino)­butan-2-one (**6a**)

To an ethanol (2.0 mL) solution of (*Z*)-4-phenyl-3-((*E*)-phenyl­(phenylimino)­methyl)-4-(phenylamino)­but-3-en-2-one **3a** (50 mg, 0.12 mmol), was added palladium on carbon (Pd/C, 20 mg, 10 wt %), the mixture was stirred under H_2_ (1 atm) for 24 h at 25 °C. Reaction was monitored by TLC, after completion of the reaction, it was filtered over a short celite bed, concentrated under reduced pressure, and purified by silica gel column chromatography using ethyl acetate/hexane (6:94) as the eluent to give compound **6a** (3*S*,4*S*)-3-benzyl-4-phenyl-4-(phenylamino)­butan-2-one (17 mg, 0.05 mmol, 43%) as white solid.

##### (3*S*,4*S*)-3-Benzyl-4-phenyl-4-(phenylamino)­butan-2-one (**6a**)

Purified by silica gel column, ethyl acetate/hexane (6:94) as eluent; white solid (17 mg, 0.05 mmol, 43%); ^1^H NMR (400 MHz, CDCl_3_): δ 7.36–7.29 (m, 4H), 7.23–7.14 (m, 4H), 7.08–7.04 (m, 2H), 7.01 (d, *J* = 8.2 Hz, 2H), 6.64 (t, *J* = 7.3 Hz, 1H), 6.50 (d, *J* = 7.6 Hz, 2H), 4.65 (d, *J* = 6.3 Hz, 1H), 4.42 (s, 1H), 3.29–3.24 (m, 1H), 3.06 (t, *J* = 13.5 Hz, 1H), 2.86 (d, *J* = 13.6 Hz, 1H), 1.72 (s, 3H); ^13^C­{^1^H} NMR (100 MHz, CDCl_3_): δ 210.4, 146.9, 140.9, 139.5, 129.1, 128.7, 128.5, 127.5, 126.9, 126.3, 117.9, 113.7, 61.3, 59.2, 33.4, 31.8, one carbon merged with other peaks; HRMS (ESI-TOF) *m*/*z*: [M + H] calcd for C_23_H_24_NO: 330.1857; found: 330.1855.

#### Procedure for the Synthesis of (1*E*,3*E*)-2-Ethylidene-*N* ^1^,*N* ^3^,1,3-tetraphenylpropane-1,3-diimine (**6b**)

To a solution of (*Z*)-4-phenyl-3-((*E*)-phenyl­(phenylimino)­methyl)-4-(phenylamino)­but-3-en-2-one **3a** (50 mg, 0.12 mmol), in dry THF (3.0 mL) at 0 °C was added LiAlH_4_ (22.7 mg, 0.60 mmol, 5.0 equiv), and the mixture was stirred for 6 h at 25 °C, confirmed the completion of reaction using TLC. Reaction mixture was quenched with a saturated solution of NH_4_Cl, followed by separation of the organic and aqueous layers. The solvent was evaporated under reduced pressure and eluted through a silica column with ethyl acetate/hexane (7:93) to yield compound **6b** (1*E*,3*E*)-2-ethylidene-*N* ^1^,*N* ^3^,1,3-tetraphenylpropane-1,3-diimine (35.5 mg, 0.08 mmol, 74%) as a brown solid.

##### (1*E*,3*E*)-2-Ethylidene-*N* ^1^,*N* ^3^,1,3-tetraphenylpropane-1,3-diimine (**6b**)

Purified by silica gel column, ethyl acetate/hexane (7:93) as eluent; brown solid (35.5 mg, 0.08 mmol, 74%); ^1^H NMR (500 MHz, CDCl_3_): δ 8.05 (d, *J* = 7.3 Hz, 2H), 7.47 (t, *J* = 2.1 Hz, 3H), 7.34 (t, *J* = 7.7 Hz, 2H), 7.14–7.08 (m, 4H), 6.98 (t, *J* = 7.4 Hz, 4H), 6.78 (t, *J* = 7.3 Hz, 1H), 6.59 (d, *J* = 7.0 Hz, 2H), 6.40 (d, *J* = 7.7 Hz, 2H), 5.91 (q, *J* = 14.2 and 7.1 Hz, 1H), 1.65 (d, *J* = 7.1 Hz, 3H); ^13^C­{^1^H} NMR (175 MHz, CDCl_3_): δ 168.4, 167.2, 152.2, 150.6, 142.0, 139.3, 138.2, 135.1, 130.6, 128.7, 128.4, 128.3, 128.1, 128.06, 128.02, 127.6, 123.5, 122.9, 120.3, 119.2, 16.7; HRMS (ESI-TOF) *m*/*z*: [M + H] calcd for C_29_H_25_N_2_: 401.2017; found: 401.2018.

#### Procedure for the Synthesis of 4-(Methyl­(phenyl)­amino)-4-phenyl-3-((*Z*)-phenyl­(phenylimino)­methyl)­but-3-en-2-one (**6c**)

A 10 mL flask was charged with NaH (5.76 mg, 0.24 mmol) and dry THF (0.5 mL). To this suspension was added a dry THF solution (2.0 mL) of compound **3a** (50 mg, 0.12 mmol) at 0 °C, and the reaction mixture was stirred for 0.5 h before methyl iodide (25.5 mg, 0.18 mmol) was added at 0 °C. The reaction was further stirred for 0.5 h at 25 °C before being filtered through a short silica bed. The crude product was purified by a silica column with ethyl acetate/hexane (9:91) to yield compound **6c** 4-(methyl­(phenyl)­amino)-4-phenyl-3-((*Z*)-phenyl­(phenylimino)­methyl)­but-3-en-2-one as a yellow oil (34.5 mg, 0.08 mmol, 67%).

##### (1*E*,3*E*)-2-Ethylidene-*N* ^1^,*N* ^3^,1,3-tetraphenylpropane-1,3-diimine (**6c**)

Purified by silica gel column, ethyl acetate/hexane (9:91) as eluent; yellow oil (34.5 mg, 0.08 mmol, 67%); ^1^H NMR for major isomer (500 MHz, CDCl_3_): δ 7.89 (d, *J* = 4.7 Hz, 2H), 7.36 (d, *J* = 4.0 Hz, 3H), 7.29 (t, *J* = 7.6 Hz, 2H), 7.15 (d, *J* = 7.7 Hz, 2H), 7.12–7.07 (m, 6H), 6.81 (t, *J* = 7.6 Hz, 2H), 6.72 (t, *J* = 7.2 Hz, 1H), 6.05 (d, *J* = 7.6 Hz, 2H), 3.19 (s, 3H), 1.51 (s, 3H); ^1^H NMR for minor isomer: δ 7.98 (d, *J* = 3.5 Hz, 2H), 7.45 (s, 3H), 7.19 (d, *J* = 5.2 Hz, 3H), 7.04 (t, *J* = 5.5 Hz, 4H), 6.97 (t, *J* = 7.2 Hz, 4H), 6.62 (d, *J* = 7.9 Hz, 2H), 6.36 (d, *J* = 7.6 Hz, 2H), 3.09 (s, 3H), 2.15 (s, 3H), rest of the peaks merged with other peaks; ^13^C­{^1^H} NMR for major isomer (125 MHz, CDCl_3_): δ 201.8, 165.8, 158.5, 151.1, 145.9, 139.6, 137.7, 130.4, 130.2, 129.92, 128.83, 128.41, 128.2, 128.16, 128.14, 125.6, 124.15, 124.13, 120.6, 119.7, 42.7, 30.4; ^13^C­{^1^H} NMR for minor isomer: 199.5, 165.2, 156.5, 150.8, 139.9, 136.5, 130.5, 129.99, 129.5, 128.88, 128.7, 128.6, 128.45, 128.0, 127.8, 123.4, 121.1, 118.9, 42.2, 29.6, rest of the peaks merged with other peaks; HRMS (ESI-TOF) *m*/*z*: [M + H]^+^ calcd for C_30_H_27_N_2_O: 431.2123; found: 431.2127.

## Supplementary Material



## Data Availability

The data underlying this study are available in the published article and its Supporting Information.

## Notes

Scheme for substrate and product preparation, mechanistic investigation, HRMS (FD) data, functionalization of (**3a**), single-crystal X-ray data, and ^1^H NMR and ^13^C NMR spectra for representative compounds (PDF)
