# Supplementary material for: Gold-Catalyzed Reactions between 2‑Alkenyl-1-arylalkynes and Nitrones via 1,2-C,N-Difunctionalizations Together with the C=C Double Bond Cleavage
Source: J Org Chem. 2025 Dec 12;90(51):18314–24. doi: 10.1021/acs.joc.5c02769 (PMC12751010; doi:10.1021/acs.joc.5c02769)

Gold-Catalyzed Reactions Between 2-Alkenyl-1-arylalkynes and Nitrones via 1,2-*C,N*-Difunctionalizations Together with the C=C Double Bond Cleavage

Vikas Ashokrao Sadaphal[*a], Hsin-Ru Wu[b], and Rai-Shung Liu[*a,b]

[a]Department of Chemistry, National Tsing Hua University, Hsinchu 30013, Taiwan, ROC.
E-mail: rsliu@mx.nthu.edu.tw

[b]Department of Chemistry and College of Semiconductor Research, National Tsing-Hua University, Hsinchu 30013, Taiwan, ROC.

Content:

(1) Schematic representation for preparation of substrate: --S2

(2) Schematic representation for catalytic operation: --S3

(3) Schematic representation for mechanistic investigation: ---S3

  3.a. Investigation for role of N-(phenyl-d_5)hydroxylamine (***d***$\boldsymbol{d_5}$**-2l**): ------------------------------S3

  3.b. HRMS (FD) data for compound $\boldsymbol{d_5}$**-3a/**$\boldsymbol{d_0}$**-3a**: ---S4

  3.c. GC-MS data for analysis of small molecules: --S5

(4) Schematic representation for chemical functionalization of **3a**: ----------------------------------S8

(5) References: ---S9

(6) X-ray crystallographic data for **4e**, **5a**, **6a**, and **6b**: ---S10

(7) ^{1}H and ^{13}C spectra of key compounds: ---S49

1. Schematic representation for preparation of substrate:

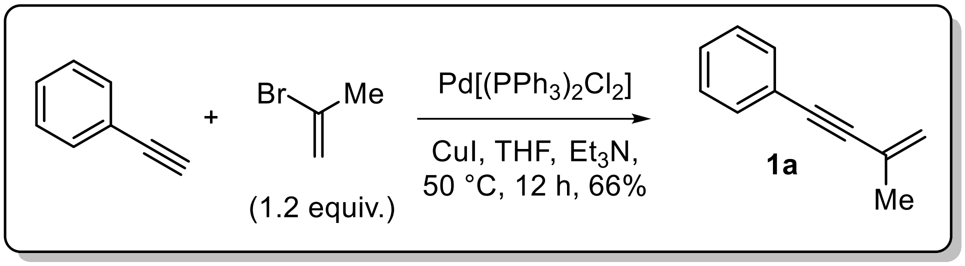

Scheme S1 Synthesis of (3-methylbut-3-en-1-yn-1-yl)benzene **(1a)**[s1]

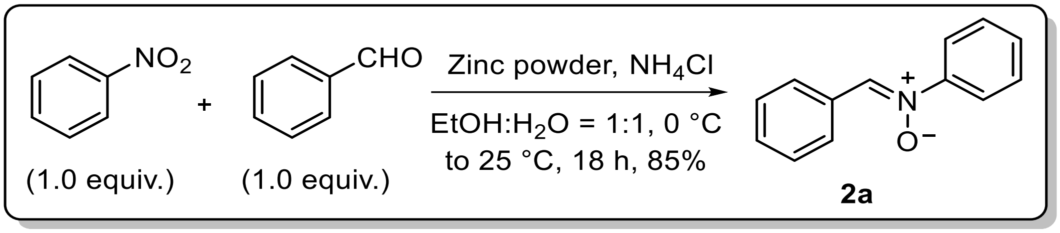

Scheme S2 Synthesis of (*Z*)-N, 1-diphenylmethanimine oxide **(2a)**[s2]

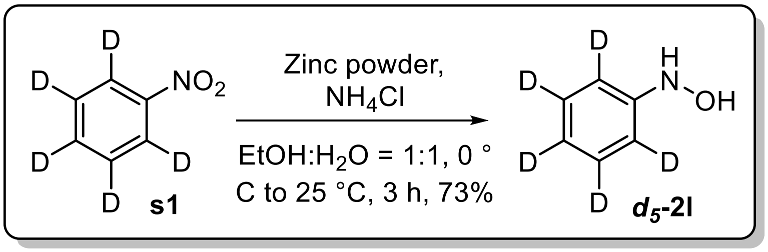

Scheme S3 Synthesis of N-(phenyl-*d*5)hydroxylamine **(*d*5-2l)**[s3]

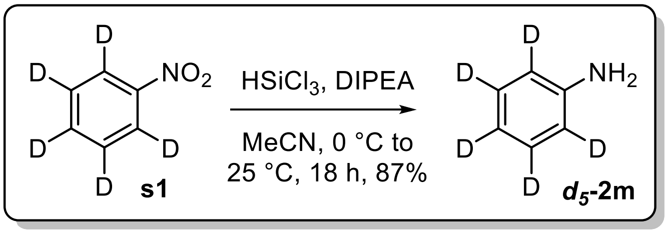

Scheme S4 Synthesis of benzen-*d*5-amine **(*d*5-2m)**[s4]

2. Schematic representation for catalytic operation:

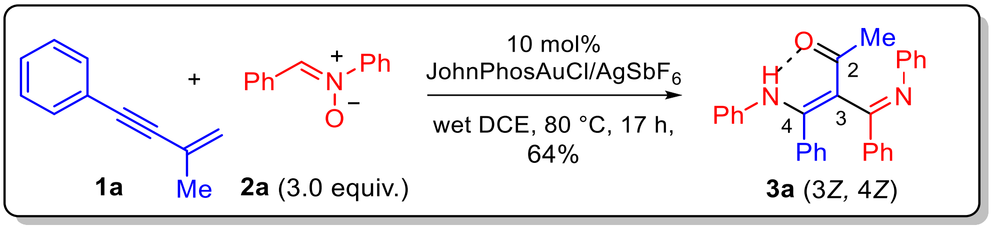

Scheme S5 Synthesis of (*Z*)-4-phenyl-3-((*E*)-phenyl(phenylimino)methyl)-4-(phenylamino)but-3-en-2-one **(3a)**

3) Schematic representation for mechanistic investigation:

3.a. Investigation for role of N-(phenyl-*d*$_5$)hydroxylamine (*d*$_5$-2l)

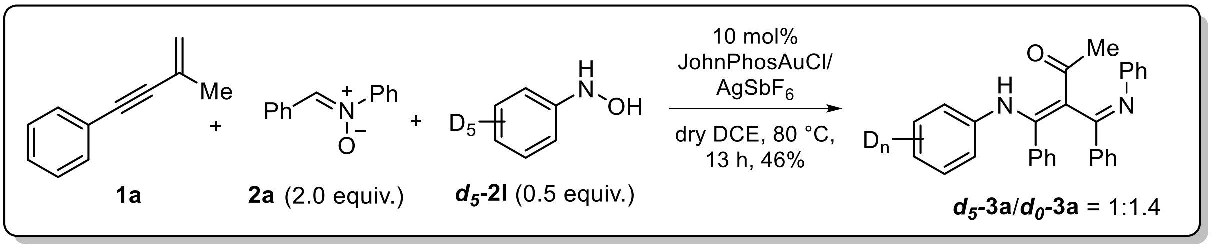

Scheme S6 Synthesis of N-(phenyl-*d*$_5$)hydroxylamine **(*d*$_5$-2l)**

3.b. High Resolution Mass Spectrometry (Field Desorption) data for compound d_5-3a/d_0-3a (1:1.4):

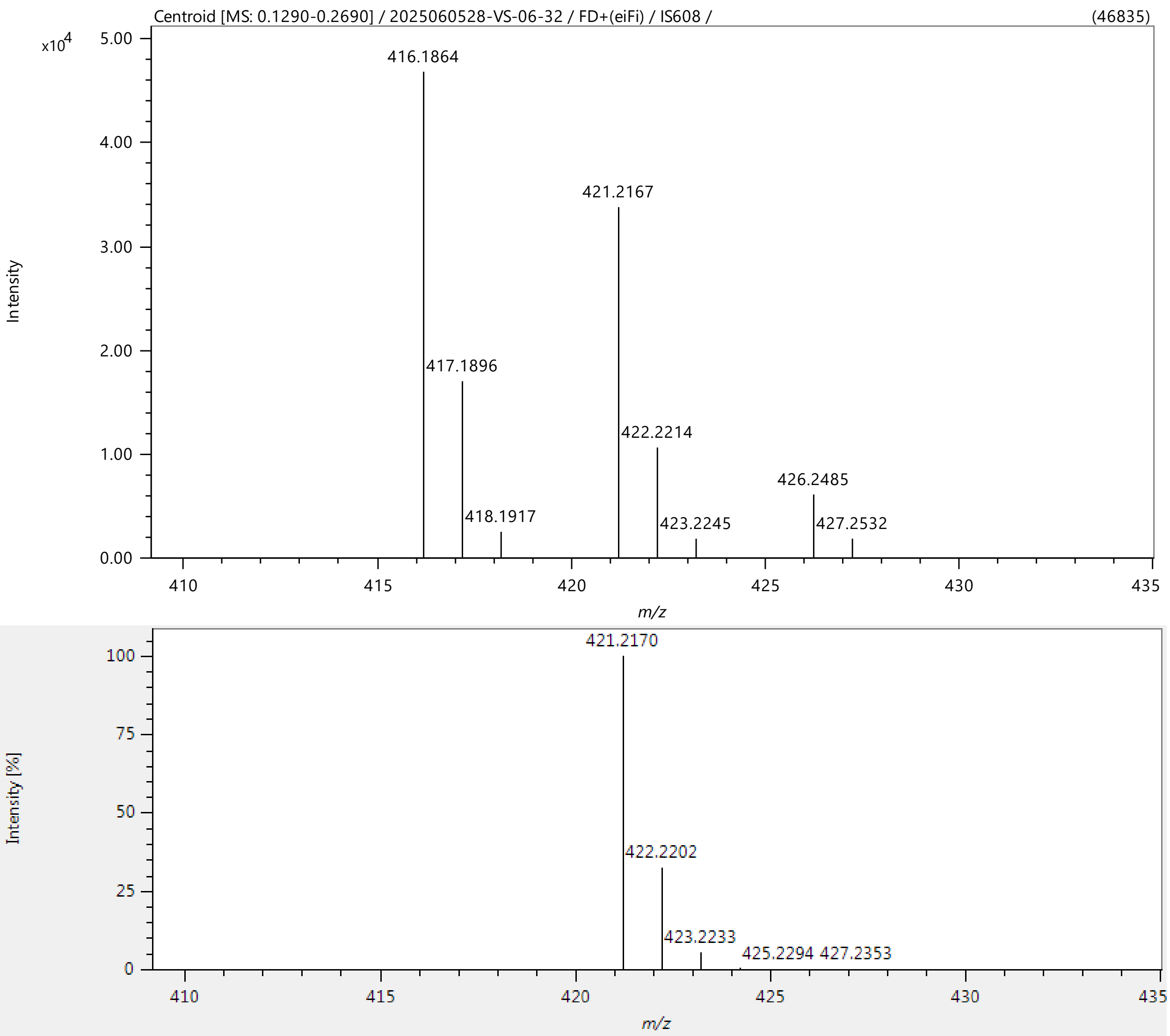

	Mass	Intensity	Formula	Calculated Mass	Mass Difference [mDa]	Mass Difference [ppm]	DBE
▶	421.21674	33754.60	C29 H19 N2 O D5	421.21695	-0.22	-0.51	21.5

Figure S1 High Resolution Mass Spectrometry (Field Desorption) spectrum for compound ***d*$_5$-3a**/***d*$_0$-3a** (1:1.4).

3.c. Gas Chromatography-Mass Spectrometry (GC-MS) data for small molecules.

GC-MS analysis of the low-molecular-weight molecules resulting from the C=C bond cleavage, confirmed the formation of benzaldehyde (PhCHO) and, notably, styrene ($PhCH=CH_2$), which was identified based on its exact mass of 104.06192 (calcd 104.06205).

i) Mass Spectrum for Minor Signals

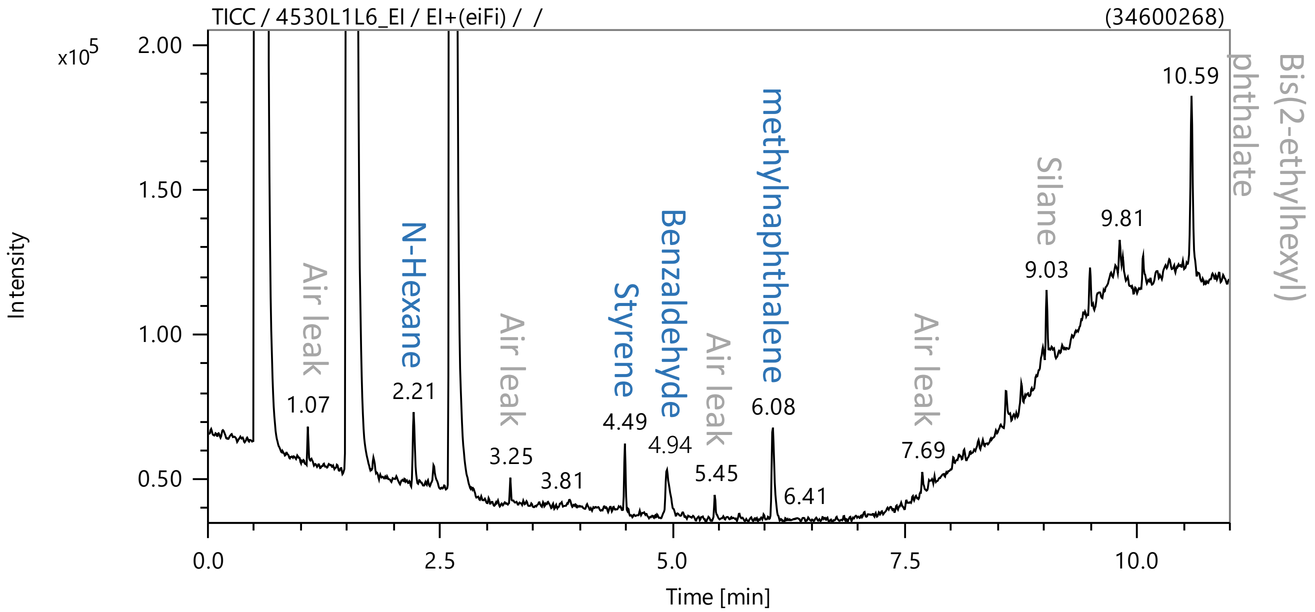

Figure S2 GC-MS data for small molecules.

ii) Mass Spectrum at 4.49 minute for Styrene

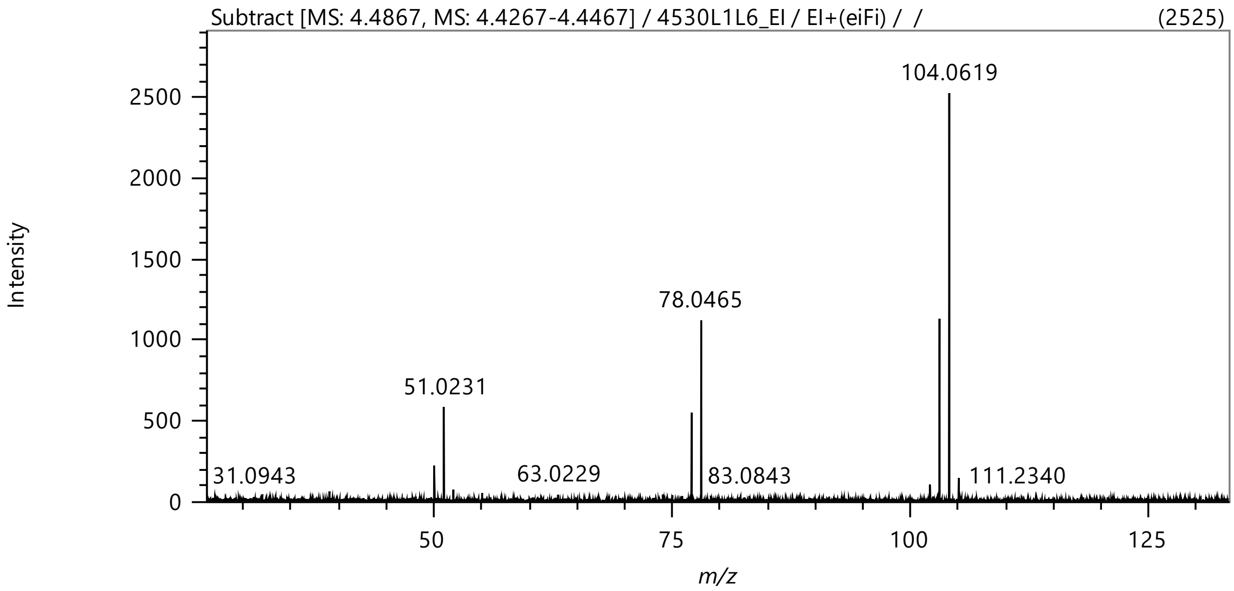

Figure S3 GC-MS data for Styrene

NIST Result

R. Match	Name
933	Styrene

Exact Mass Calculation

Mass	Intensity	Formula	Calculated Mass	Mass Difference (mDa)	DBE
104.06192	2524.58	C8H8	104.06205	-0.13	5.0

iii) Mass Spectrum at 4.94 minute for Benzaldehyde

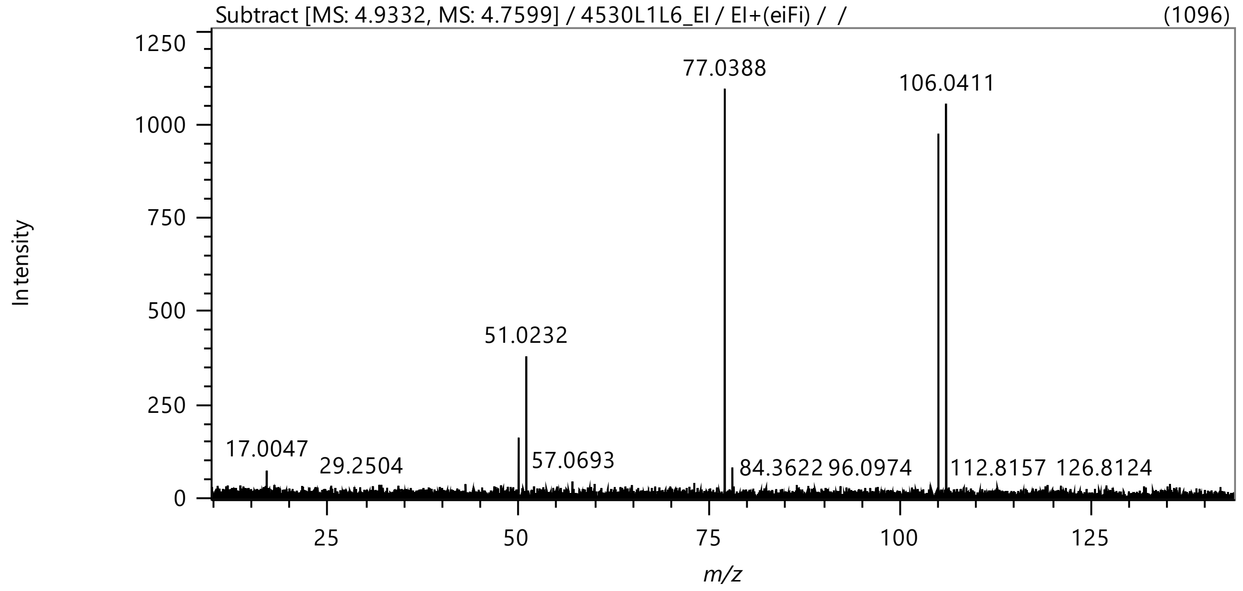

Figure S4 GC-MS data for Benzaldehyde

NIST Result

R. Match	Name
825	Benzaldehyde

Exact Mass Calculation

Mass	Intensity	Formula	Calculated Mass	Mass Difference (mDa)	DBE
106.04111	1056.36	C7H6O	106.04132	-0.21	5.0

4. Schematic representation for chemical functionalization of 3a:

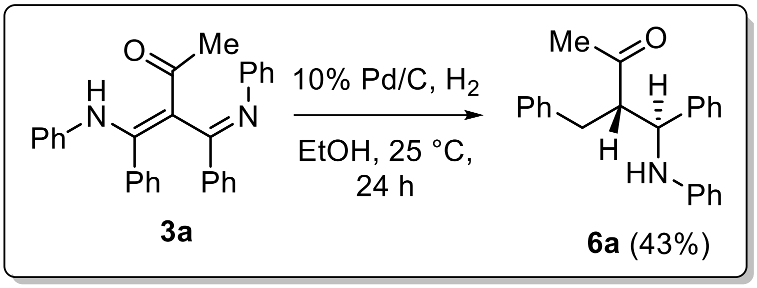

Scheme S7 Synthesis of (3*S*,4*S*)-3-benzyl-4-phenyl-4-(phenylamino)butan-2-one **(6a)**

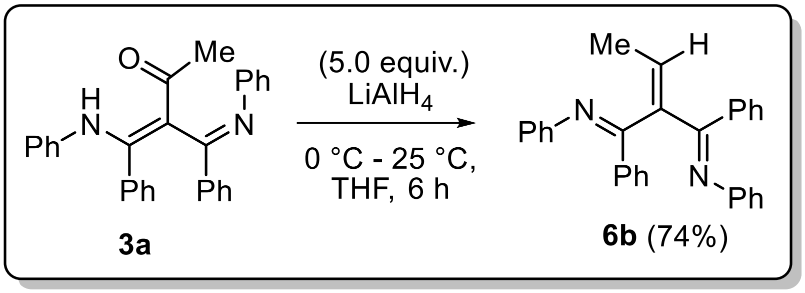

Scheme S8 Synthesis of (1*E*,3*E*)-2-ethylidene-N^1, N^3,1,3-tetraphenylpropane-1,3-diimine **(6b)**

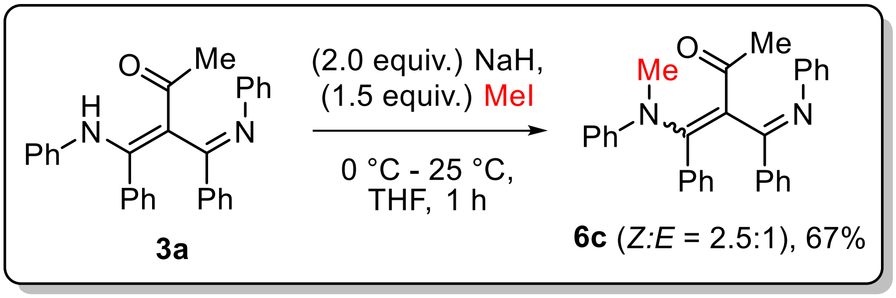

Scheme S9 Synthesis of 4-(methyl(phenyl)amino)-4-phenyl-3-((Z)-phenyl(phenylimino)methyl)but-3-en-2-one **(6c)**

5. References:

[s1] (a) Zhang, K.-F.; Bian, K.-J.; Li, C.; Sheng, J.; Li, Y.; Wang, X.-S. *Angew. Chem., Int. Ed.* **2019**, *58*, 5069–5074. (b) Zhang, Y.; Yu, B.; Gao, B.; Zhang, T.; Huang, H. *Org. Lett.* **2019**, *21*, 535–539. (c) Picher, M.-I.; Plietker, B. *Org. Lett.* **2020**, *22*, 340–344. (d) Haubenreisser, S.; Hensenne, P.; Schröder, S.; Niggemann, M. *Org. Lett.* **2013**, *15*, 2262–2265. (e) Sadamitsu, Y.; Komatsuki, K.; Saito, K.; Yamada, T. *Org. Lett.* **2017**, *19*, 3191–3194.

[s2] (a) Vasu, D.; Liu, R.-S. *Chem. Eur. J.* **2012**, *18*, 13638–13641. (b) Lo, M. M.-C.; Fu, G. C. *J. Am. Chem. Soc.* **2002**, *124*, 4572–4573. (c) Evans, D. A.; Song, H.-J.; Fandrick, K. R. *Org. Lett.* **2006**, *8*, 3351–3354. (d) West, P. R.; Davis, G. C. *J. Org. Chem.* **1989**, *54*, 5176–5179.

[s3] Ghorpade, S.; Liu, R.-S. *Angew. Chem., Int. Ed.* **2014**, *53*, 12885−12888.

[s4] Orlandi, M.; Tosi, F.; Bonsignore, M.; Benaglia, M. *Org. Lett*. **2015**, *17*, 3941-3943.

6. X-ray crystallographic structure and data for compound 4e, 5a, 6a, and 6b:

(a) X-ray crystallographic data of compound (4e)

Ellipsoid contour % probability level = 50%

Sample Preparation for Crystal Growth: The compound **4e** was dissolved in Ethyl acetate/Hexane (1:5) and kept for slow evaporation (7 days). A needle-shaped crystal was formed, and its X-ray analysis was performed.

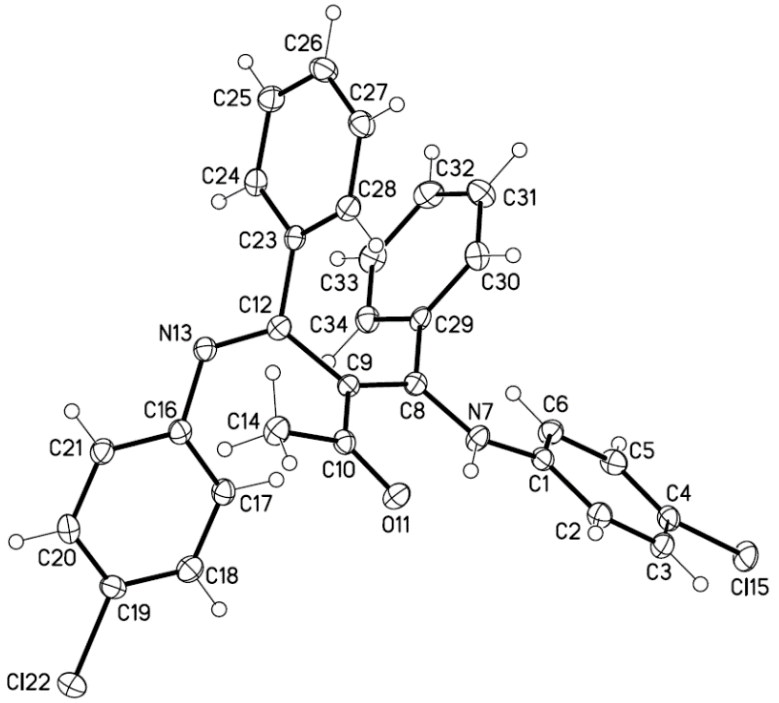

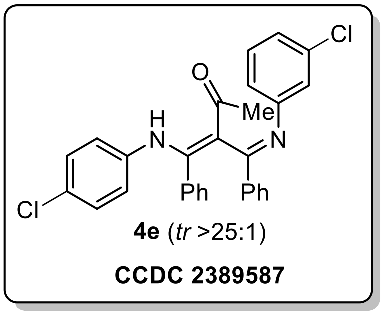

241003LT_auto

Table S1 Crystal data and structure refinement for 241003LT_auto.

Identification code	241003LT_auto
Empirical formula	$C_{29}H_{22}Cl_2N_2O$

Formula weight	485.38
Temperature/K	100.01(10)
Crystal system	monoclinic
Space group	$P2_1/n$
a/Å	10.19776(11)
b/Å	9.79728(12)
c/Å	23.7390(3)
α/°	90
β/°	94.6964(10)
γ/°	90
Volume/Å^3	2363.80(5)
Z	4
ρ_{calc}g/cm^3	1.364
μ/mm^{-1}	2.665
F(000)	1008.0
Crystal size/mm^3	0.09 × 0.07 × 0.06
Radiation	Cu Kα (λ = 1.54184)
2Θ range for data collection/°	7.474 to 146.052
Index ranges	-9 ≤ h ≤ 12, -11 ≤ k ≤ 12, -27 ≤ l ≤ 28
Reflections collected	19152
Independent reflections	4571 [R_{int} = 0.0299, R_{sigma} = 0.0275]
Data/restraints/parameters	4571/0/313
Goodness-of-fit on F^2	1.049
Final R indexes [I>=2σ (I)]	R_1 = 0.0323, wR_2 = 0.0775
Final R indexes [all data]	R_1 = 0.0371, wR_2 = 0.0800
Largest diff. peak/hole / e Å^{-3}	0.36/-0.24

Table S2 Fractional Atomic Coordinates (×10[4]) and Equivalent Isotropic Displacement Parameters (Å[2]×10[3]) for 241003LT_auto. U_{eq} is defined as 1/3 of the trace of the orthogonalised U_{IJ} tensor.

Atom	*x*	*y*	*z*	U(eq)
Cl15	3771.0(4)	1291.3(4)	9857.8(2)	21.28(11)
Cl22	6950.3(4)	9345.2(4)	6687.1(2)	24.39(11)
O11	7291.8(10)	2575.2(12)	6999.8(4)	18.7(2)
N7	5174.2(12)	1931.1(14)	7496.4(5)	16.0(3)
N13	4295.4(12)	4299.7(13)	5794.5(5)	15.4(3)
C1	4740.7(14)	1710.1(16)	8038.3(6)	14.9(3)
C2	5715.6(15)	1335.8(16)	8459.1(6)	16.9(3)
C3	5435.9(15)	1197.5(16)	9017.2(6)	17.8(3)
C4	4157.1(15)	1422.4(16)	9158.1(6)	17.3(3)
C5	3178.8(15)	1777.4(17)	8749.4(7)	17.8(3)
C6	3461.2(15)	1919.6(16)	8190.0(7)	17.5(3)
C8	4501.8(14)	2226.6(15)	6993.8(6)	14.6(3)
C9	5148.0(14)	2685.7(15)	6538.4(6)	14.1(3)
C10	6576.8(14)	2784.2(16)	6560.2(6)	14.9(3)
C12	4338.3(14)	3090.6(16)	6009.9(6)	14.6(3)
C14	7213.9(15)	3061.5(18)	6019.3(7)	19.5(3)
C16	4966.3(14)	5439.5(16)	6047.3(6)	15.4(3)
C17	5038.9(14)	5775.2(16)	6623.8(6)	16.9(3)
C18	5633.1(15)	6980.3(17)	6817.2(7)	18.8(3)
C19	6173.3(14)	7850.2(16)	6438.6(7)	17.4(3)
C20	6112.7(15)	7547.4(16)	5866.8(7)	19.2(3)
C21	5502.5(15)	6348.3(16)	5674.5(6)	17.8(3)
C23	3545.3(14)	2019.2(16)	5692.6(6)	14.7(3)

C24	2310.5(15)	2334.0(17)	5421.5(6)	17.7(3)
C25	1582.2(15)	1329.9(17)	5129.2(7)	20.2(3)
C26	2076.9(15)	13.4(17)	5090.8(7)	21.2(3)
C27	3304.3(15)	-302.3(17)	5352.0(7)	20.2(3)
C28	4025.0(15)	690.9(16)	5658.5(6)	17.7(3)
C29	3048.1(14)	2007.7(16)	6926.4(6)	14.2(3)
C30	2564.2(15)	681.1(17)	6911.0(7)	18.1(3)
C31	1212.3(15)	455.8(18)	6832.1(7)	21.9(4)
C32	353.0(15)	1553.0(18)	6774.2(7)	21.5(4)
C33	838.0(15)	2876.0(18)	6792.5(7)	20.5(3)
C34	2185.5(15)	3108.2(17)	6862.3(6)	17.5(3)

Table S3 Anisotropic Displacement Parameters (Å^2×10^3) for 241003LT_auto. The Anisotropic displacement factor exponent takes the form: -2π^2[h^{2}a*^{2}U$_{11}$+2hka*b*U$_{12}$+…].

Atom	U_{11}	U_{22}	U_{33}	U_{23}	U_{13}	U_{12}
Cl15	28.4(2)	23.6(2)	12.75(18)	1.09(15)	7.04(14)	-0.80(16)
Cl22	29.7(2)	17.3(2)	25.3(2)	-1.57(16)	-2.67(16)	-4.97(16)
O11	15.1(5)	26.1(6)	14.9(5)	0.5(5)	0.9(4)	-0.1(5)
N7	12.6(6)	22.7(7)	12.8(6)	2.4(5)	1.8(5)	0.3(5)
N13	15.9(6)	16.8(7)	13.8(6)	0.8(5)	2.2(5)	-0.8(5)
C1	18.0(7)	13.8(7)	13.1(7)	-0.5(6)	2.9(6)	-1.7(6)
C2	15.4(7)	18.5(8)	17.3(8)	0.9(6)	2.9(6)	-0.2(6)
C3	21.0(8)	18.0(8)	14.2(7)	1.1(6)	0.5(6)	0.2(6)
C4	24.8(8)	15.7(8)	12.0(7)	-0.4(6)	5.0(6)	-3.5(6)

C5	16.9(7)	19.8(8)	17.5(8)	-2.0(6)	5.6(6)	-2.5(6)
C6	17.6(7)	19.5(8)	15.4(7)	0.1(6)	0.9(6)	-0.5(6)
C8	15.9(7)	13.6(7)	14.3(7)	-0.6(6)	1.3(6)	1.5(6)
C9	15.9(7)	14.1(7)	12.4(7)	-0.3(6)	1.4(5)	-0.9(6)
C10	16.9(7)	13.9(7)	14.4(7)	0.4(6)	3.3(6)	0.2(6)
C12	12.6(7)	18.8(8)	12.8(7)	-0.1(6)	3.9(5)	0.7(6)
C14	16.4(7)	25.9(9)	16.7(8)	2.1(7)	4.2(6)	0.8(7)
C16	13.0(7)	15.5(8)	17.6(7)	1.8(6)	0.4(5)	2.1(6)
C17	16.4(7)	18.5(8)	16.0(7)	2.2(6)	3.1(6)	-0.1(6)
C18	19.4(8)	21.3(8)	15.8(8)	-1.4(6)	1.9(6)	1.9(6)
C19	15.5(7)	14.8(8)	21.6(8)	-1.2(6)	-0.9(6)	-0.2(6)
C20	21.1(8)	16.8(8)	20.0(8)	4.9(7)	3.1(6)	0.2(6)
C21	21.5(8)	17.8(8)	14.2(7)	0.4(6)	1.5(6)	0.7(6)
C23	16.8(7)	17.3(8)	10.5(7)	1.6(6)	3.7(5)	-1.3(6)
C24	18.2(7)	18.0(8)	17.1(8)	3.8(6)	2.1(6)	0.7(6)
C25	15.4(7)	23.8(9)	20.9(8)	2.9(7)	-0.8(6)	-2.1(6)
C26	21.2(8)	20.9(8)	21.6(8)	-1.3(7)	2.5(6)	-6.6(7)
C27	24.2(8)	16.2(8)	20.4(8)	-1.2(7)	3.5(6)	-0.5(7)
C28	17.2(7)	19.7(8)	16.2(7)	1.2(6)	0.9(6)	0.3(6)
C29	15.0(7)	18.5(8)	9.3(7)	2.0(6)	1.5(5)	0.3(6)
C30	17.7(8)	17.5(8)	19.6(8)	2.2(6)	4.1(6)	2.5(6)
C31	19.8(8)	19.3(8)	26.7(9)	0.4(7)	3.1(6)	-3.4(7)
C32	14.2(7)	29.5(9)	20.9(8)	-0.3(7)	2.3(6)	-1.5(7)
C33	17.7(8)	23.3(9)	20.7(8)	1.7(7)	2.5(6)	5.5(7)
C34	20.1(7)	17.2(8)	15.6(7)	0.9(6)	3.1(6)	0.8(6)

Table S4 Bond Lengths for 241003LT_auto.

Atom	Atom	Length/Å	Atom	Atom	Length/Å
Cl15	C4	1.7430(15)	C16	C17	1.403(2)
Cl22	C19	1.7449(16)	C16	C21	1.398(2)
O11	C10	1.2401(18)	C17	C18	1.388(2)
N7	C1	1.4107(18)	C18	C19	1.385(2)
N7	C8	1.3578(19)	C19	C20	1.386(2)
N13	C12	1.290(2)	C20	C21	1.389(2)
N13	C16	1.417(2)	C23	C24	1.401(2)
C1	C2	1.399(2)	C23	C28	1.395(2)
C1	C6	1.397(2)	C24	C25	1.384(2)
C2	C3	1.384(2)	C25	C26	1.391(2)
C3	C4	1.390(2)	C26	C27	1.386(2)
C4	C5	1.378(2)	C27	C28	1.389(2)
C5	C6	1.389(2)	C29	C30	1.390(2)
C8	C9	1.386(2)	C29	C34	1.392(2)
C8	C29	1.493(2)	C30	C31	1.394(2)
C9	C10	1.457(2)	C31	C32	1.387(2)
C9	C12	1.498(2)	C32	C33	1.387(2)
C10	C14	1.510(2)	C33	C34	1.389(2)
C12	C23	1.491(2)			

Table S5 Bond Angles for 241003LT_auto.

Atom	Atom	Atom	Angle/˚	Atom	Atom	Atom	Angle/˚
C8	N7	C1	131.35(13)	C21	C16	N13	115.72(13)
C12	N13	C16	123.80(13)	C21	C16	C17	118.42(14)

C2	C1	N7	115.71(13)	C18	C17	C16	120.58(14)
C6	C1	N7	125.56(14)	C19	C18	C17	119.65(14)
C6	C1	C2	118.58(14)	C18	C19	Cl22	119.39(12)
C3	C2	C1	121.25(14)	C18	C19	C20	121.04(15)
C2	C3	C4	119.05(14)	C20	C19	Cl22	119.57(12)
C3	C4	Cl15	120.48(12)	C19	C20	C21	119.07(15)
C5	C4	Cl15	118.85(12)	C20	C21	C16	121.23(14)
C5	C4	C3	120.66(14)	C24	C23	C12	120.47(14)
C4	C5	C6	120.25(14)	C28	C23	C12	120.51(13)
C5	C6	C1	120.20(14)	C28	C23	C24	119.02(14)
N7	C8	C9	121.09(13)	C25	C24	C23	119.93(15)
N7	C8	C29	119.27(13)	C24	C25	C26	120.62(14)
C9	C8	C29	119.61(13)	C27	C26	C25	119.80(15)
C8	C9	C10	122.11(13)	C26	C27	C28	119.87(15)
C8	C9	C12	118.32(13)	C27	C28	C23	120.73(14)
C10	C9	C12	119.57(13)	C30	C29	C8	118.97(14)
O11	C10	C9	122.44(13)	C30	C29	C34	120.11(14)
O11	C10	C14	118.74(13)	C34	C29	C8	120.89(14)
C9	C10	C14	118.70(13)	C29	C30	C31	119.79(15)
N13	C12	C9	125.03(14)	C32	C31	C30	120.05(15)
N13	C12	C23	116.54(13)	C31	C32	C33	120.04(14)
C23	C12	C9	118.40(13)	C32	C33	C34	120.23(15)
C17	C16	N13	125.65(14)	C33	C34	C29	119.76(15)

Table S6 Torsion Angles for 241003LT_auto.

A	B	C	D	Angle/˚	A	B	C	D	Angle/˚
Cl15	C4	C5	C6	178.59(12)	C9	C12	C23	C28	-36.15(19)
Cl22	C19	C20	C21	179.41(12)	C10	C9	C12	N13	-62.5(2)
N7	C1	C2	C3	174.73(14)	C10	C9	C12	C23	115.50(16)
N7	C1	C6	C5	-174.48(15)	C12	N13	C16	C17	-43.1(2)
N7	C8	C9	C10	4.4(2)	C12	N13	C16	C21	142.30(15)
N7	C8	C9	C12	-175.58(14)	C12	C9	C10	O11	173.74(14)
N7	C8	C29	C30	-70.06(19)	C12	C9	C10	C14	-10.3(2)
N7	C8	C29	C34	111.91(17)	C12	C23	C24	C25	-179.87(14)
N13	C12	C23	C24	-37.6(2)	C12	C23	C28	C27	-178.43(14)
N13	C12	C23	C28	142.04(14)	C16	N13	C12	C9	-3.4(2)
N13	C16	C17	C18	-174.63(14)	C16	N13	C12	C23	178.59(12)
N13	C16	C21	C20	175.99(14)	C16	C17	C18	C19	-0.8(2)
C1	N7	C8	C9	166.15(15)	C17	C16	C21	C20	1.0(2)
C1	N7	C8	C29	-15.9(3)	C17	C18	C19	Cl22	-178.61(12)
C1	C2	C3	C4	0.7(2)	C17	C18	C19	C20	1.0(2)
C2	C1	C6	C5	1.0(2)	C18	C19	C20	C21	-0.2(2)
C2	C3	C4	Cl15	-178.75(12)	C19	C20	C21	C16	-0.8(2)
C2	C3	C4	C5	0.0(2)	C21	C16	C17	C18	-0.2(2)
C3	C4	C5	C6	-0.2(2)	C23	C24	C25	C26	-1.4(2)
C4	C5	C6	C1	-0.3(2)	C24	C23	C28	C27	1.2(2)
C6	C1	C2	C3	-1.2(2)	C24	C25	C26	C27	0.7(2)

C8	N7	C1	C2	175.98(16)	C25	C26	C27	C28	1.0(2)
C8	N7	C1	C6	-8.4(3)	C26	C27	C28	C23	-2.0(2)
C8	C9	C10	O11	-6.2(2)	C28	C23	C24	C25	0.5(2)
C8	C9	C10	C14	169.73(14)	C29	C8	C9	C10	-173.55(14)
C8	C9	C12	N13	117.43(17)	C29	C8	C9	C12	6.5(2)
C8	C9	C12	C23	-64.54(19)	C29	C30	C31	C32	-0.6(2)
C8	C29	C30	C31	-178.16(14)	C30	C29	C34	C33	1.1(2)
C8	C29	C34	C33	179.11(14)	C30	C31	C32	C33	0.3(2)
C9	C8	C29	C30	107.91(17)	C31	C32	C33	C34	0.7(2)
C9	C8	C29	C34	-70.1(2)	C32	C33	C34	C29	-1.4(2)
C9	C12	C23	C24	144.23(14)	C34	C29	C30	C31	-0.1(2)

Table S7 Hydrogen Atom Coordinates (Å×10^4) and Isotropic Displacement Parameters (Å^2×10^3) for 241003LT_auto.

Atom	*x*	*y*	*z*	U(eq)
H7	6030(20)	2020(20)	7478(8)	32(5)
H2	6585.84	1173.49	8359.99	20
H3	6108.19	952.34	9299.79	21
H5	2308.02	1925.48	8850.76	21
H6	2782.13	2159.98	7909.64	21
H14A	7078.64	4019.79	5911.43	29
H14B	6817.49	2471.84	5718.33	29
H14C	8159.07	2872.64	6077.27	29
H17	4678.25	5172.29	6883.84	20
H18	5669.14	7207.31	7207.26	23

H20	6483.32	8151.44	5609.97	23
H21	5448.48	6141.9	5282.23	21
H24	1973.08	3235.68	5438.29	21
H25	736.63	1542.12	4953.18	24
H26	1574.83	-667.39	4886.09	25
H27	3652.12	-1196.3	5321.55	24
H28	4853.89	463.2	5847	21
H30	3153.15	-69.94	6954.04	22
H31	879.19	-450.14	6817.98	26
H32	-568.59	1398.36	6722	26
H33	246.82	3626.27	6757.13	25
H34	2516.93	4014.61	6866.27	21

(b) X-ray crystallographic data of compound (5a)

Ellipsoid contour % probability level = 50%

Sample Preparation for Crystal Growth: The compound **5a** was dissolved in Ethyl acetate/Hexane (1:5) and kept for slow evaporation (3 days). A rod-shaped crystal was formed, and its X-ray analysis was performed.

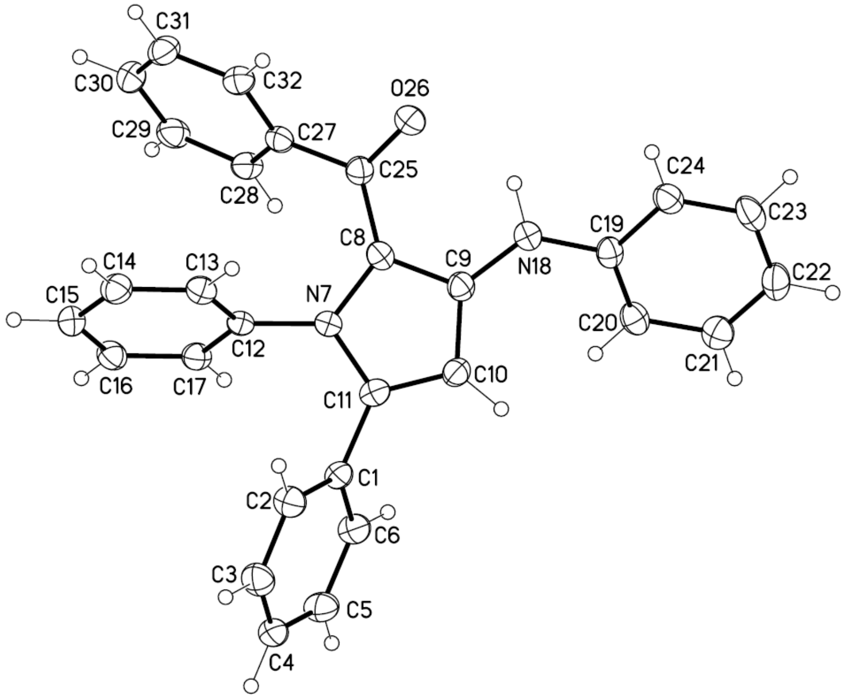

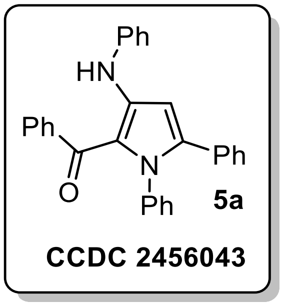

2503147lt_auto

Table S8 Crystal data and structure refinement for 2503147lt_auto.

Identification code	2503147lt_auto
Empirical formula	$C_{29}H_{22}N_2O$
Formula weight	414.48
Temperature/K	100.00(10)
Crystal system	orthorhombic
Space group	$P2_12_12$
a/Å	30.2689(7)
b/Å	11.4368(3)
c/Å	6.25354(13)
α/°	90
β/°	90

γ/°	90
Volume/Å^3	2164.86(9)
Z	4
ρ_{calc}g/cm^3	1.272
μ/mm^{-1}	0.604
F(000)	872.0
Crystal size/mm^3	0.23 × 0.06 × 0.06
Radiation	Cu Kα (λ = 1.54184)
2Θ range for data collection/°	8.264 to 146.386
Index ranges	-36 ≤ h ≤ 37, -13 ≤ k ≤ 13, -7 ≤ l ≤ 6
Reflections collected	19467
Independent reflections	4209 [R_{int} = 0.0417, R_{sigma} = 0.0374]
Data/restraints/parameters	4209/0/294
Goodness-of-fit on F^2	1.061
Final R indexes [I>=2σ (I)]	R_1 = 0.0389, wR_2 = 0.0956
Final R indexes [all data]	R_1 = 0.0456, wR_2 = 0.0984
Largest diff. peak/hole / e Å^{-3}	0.18/-0.21
Flack parameter	-0.03(17)

Table S9 Fractional Atomic Coordinates (×10^4) and Equivalent Isotropic Displacement Parameters (Å^2×10^3) for 2503147lt_auto. U_{eq} is defined as 1/3 of the trace of the orthogonalised U_{IJ} tensor.

Atom	*x*	*y*	*z*	U(eq)
O26	3133.5(6)	5294.5(16)	9066(3)	30.4(4)
N7	3739.2(6)	3988.5(17)	4645(3)	21.2(4)
N18	3798.6(7)	6674.8(18)	7563(3)	24.8(5)
C1	4391.3(7)	3807(2)	2241(4)	23.4(5)

Table S9 Fractional Atomic Coordinates (×10^4) and Equivalent Isotropic Displacement Parameters (Å^2×10^3) for 2503147lt_auto. U_{eq} is defined as 1/3 of the trace of the orthogonalised U_{IJ} tensor.

Atom	*x*	*y*	*z*	U(eq)
C2	4587.1(8)	2739(2)	2813(4)	28.0(6)
C3	4855.1(8)	2153(3)	1391(4)	31.4(6)
C4	4931.2(8)	2608(2)	-638(4)	30.2(6)
C5	4738.8(8)	3660(3)	-1223(4)	32.0(6)
C6	4472.0(8)	4262(2)	210(4)	28.1(6)
C8	3569.8(8)	4776(2)	6171(4)	22.5(5)
C9	3857.1(7)	5751(2)	6174(4)	22.6(5)
C10	4188.7(7)	5548(2)	4625(4)	24.0(5)
C11	4110.6(7)	4459(2)	3763(4)	22.8(5)
C12	3576.4(7)	2834(2)	4187(4)	20.9(5)
C13	3543.7(8)	2011(2)	5799(4)	23.9(5)
C14	3376.8(8)	909(2)	5331(4)	27.4(6)
C15	3248.6(8)	632(2)	3265(4)	27.0(6)
C16	3290.0(8)	1459(2)	1653(4)	26.1(6)
C17	3453.8(7)	2565(2)	2112(4)	23.5(5)
C19	3980.0(7)	7799(2)	7458(4)	22.9(5)
C20	4178.1(8)	8248(2)	5629(4)	25.5(5)
C21	4369.2(8)	9356(2)	5686(4)	28.9(6)
C22	4359.1(9)	10027(2)	7524(4)	31.5(6)
C23	4142.9(8)	9594(2)	9318(4)	30.5(6)
C24	3954.3(8)	8493(2)	9298(4)	26.0(5)
C25	3172.8(8)	4689(2)	7419(4)	22.6(5)
C27	2797.5(7)	3908(2)	6787(4)	22.6(5)
C28	2640.7(7)	3861(2)	4689(4)	24.7(5)

Table S9 Fractional Atomic Coordinates (×10^4) and Equivalent Isotropic Displacement Parameters (Å^2×10^3) for 2503147lt_auto. U_{eq} is defined as 1/3 of the trace of the orthogonalised U_{IJ} tensor.

Atom	*x*	*y*	*z*	U(eq)
C29	2283.0(8)	3142(2)	4203(4)	29.7(6)
C30	2094.3(8)	2447(2)	5775(4)	30.9(6)
C31	2248.2(8)	2501(2)	7861(4)	28.2(6)
C32	2593.5(8)	3251(2)	8367(4)	25.6(5)

Table S10 Anisotropic Displacement Parameters (Å^2×10^3) for 2503147lt_auto. The Anisotropic displacement factor exponent takes the form: $-2\pi^2[h^2a^{*2}U_{11}+2hka^*b^*U_{12}+\ldots]$.

Atom	U_{11}	U_{22}	U_{33}	U_{23}	U_{13}	U_{12}
O26	31.3(9)	31.6(10)	28.1(9)	-7.8(8)	5.5(7)	-3.6(8)
N7	20.7(9)	21.0(11)	22.0(9)	-2.1(8)	2.9(8)	0.5(8)
N18	25.6(10)	22.7(11)	26.1(10)	-3.2(10)	2.2(9)	-1.4(9)
C1	18.2(11)	25.6(13)	26.3(12)	-2.5(11)	0.5(9)	-2.2(10)
C2	29.4(13)	27.5(14)	27.0(12)	1.3(11)	1.0(10)	2.2(11)
C3	25.7(13)	29.8(15)	38.5(13)	-3.9(12)	-2.0(10)	5.2(11)
C4	23.7(12)	33.2(15)	33.7(13)	-10.1(14)	5.0(10)	-4.7(11)
C5	32.4(14)	36.9(16)	26.6(12)	0.0(12)	6.9(10)	-2.8(12)
C6	26.0(12)	29.5(15)	28.9(13)	1.3(11)	2.2(10)	-0.2(11)
C8	23.6(12)	21.1(13)	22.8(11)	-2.3(10)	-0.7(9)	1.8(10)
C9	20.1(11)	22.9(13)	24.8(11)	-2.4(10)	-2.8(9)	-0.1(10)
C10	20.8(11)	22.2(13)	29.1(12)	0.7(11)	1.6(9)	-1.6(10)
C11	20.0(11)	24.6(14)	23.8(11)	3.7(10)	1.0(9)	0.7(10)
C12	17.8(11)	19.9(12)	24.9(11)	-1.7(10)	3.4(8)	1.9(9)
C13	24.3(11)	25.8(13)	21.7(11)	-0.9(11)	0.5(9)	1.8(10)
C14	29.0(12)	26.1(14)	26.9(12)	2.9(11)	3.3(10)	0.0(11)

Table S10 Anisotropic Displacement Parameters (Å^2×10^3) for 2503147lt_auto. The Anisotropic displacement factor exponent takes the form: $-2\pi^2[h^2a^{*2}U_{11}+2hka^*b^*U_{12}+…]$.

Atom	U_{11}	U_{22}	U_{33}	U_{23}	U_{13}	U_{12}
C15	27.0(12)	21.6(13)	32.3(13)	-3.0(11)	3.8(10)	-2.2(11)
C16	25.8(12)	28.4(14)	24.1(12)	-5.8(11)	0.3(9)	0.1(11)
C17	23.0(11)	25.4(13)	22.2(11)	-0.7(11)	2.0(9)	0.4(10)
C19	21.4(11)	20.8(13)	26.5(11)	-1.4(11)	-5.1(9)	0.7(10)
C20	27.3(12)	24.1(14)	25.1(12)	-3.1(11)	-5.6(10)	2.8(10)
C21	29.2(13)	26.7(14)	30.9(13)	2.4(12)	-1.8(10)	2.2(11)
C22	29.8(13)	24.6(14)	40.1(14)	-4.2(12)	-6.7(11)	-2.3(11)
C23	31.0(13)	28.0(14)	32.7(13)	-11.8(13)	-7.2(11)	4.1(11)
C24	24.8(12)	26.0(14)	27.2(12)	-3.5(12)	-0.8(10)	3.0(10)
C25	23.7(12)	21.4(12)	22.7(11)	-0.2(11)	0.6(9)	-0.4(10)
C27	20.0(11)	22.0(13)	25.7(11)	-3.4(10)	1.7(9)	2.6(10)
C28	21.6(11)	29.1(14)	23.4(11)	-1.1(11)	1.8(9)	1.5(10)
C29	24.4(12)	38.3(15)	26.4(12)	-9.3(12)	-0.8(10)	-0.2(11)
C30	24.2(12)	29.5(14)	39.1(13)	-6.7(13)	1.9(11)	-4.8(11)
C31	26.3(12)	27.9(14)	30.5(13)	3.6(12)	5.5(9)	-2.2(11)
C32	23.2(12)	27.3(14)	26.4(12)	1.2(11)	1.4(9)	1.7(11)

Table S11 Bond Lengths for 2503147lt_auto.

Atom	Atom	Length/Å	Atom	Atom	Length/Å
O26	C25	1.247(3)	C12	C17	1.384(3)
N7	C8	1.409(3)	C13	C14	1.389(3)
N7	C11	1.363(3)	C14	C15	1.386(3)
N7	C12	1.438(3)	C15	C16	1.388(3)

Table S11 Bond Lengths for 2503147lt_auto.

Atom	Atom	Length/Å	Atom	Atom	Length/Å
N18	C9	1.379(3)	C16	C17	1.389(3)
N18	C19	1.400(3)	C19	C20	1.390(3)
C1	C2	1.403(3)	C19	C24	1.400(3)
C1	C6	1.395(3)	C20	C21	1.393(4)
C1	C11	1.478(3)	C21	C22	1.382(4)
C2	C3	1.378(3)	C22	C23	1.390(4)
C3	C4	1.391(4)	C23	C24	1.383(4)
C4	C5	1.385(4)	C25	C27	1.498(3)
C5	C6	1.389(4)	C27	C28	1.397(3)
C8	C9	1.414(3)	C27	C32	1.386(3)
C8	C25	1.436(3)	C28	C29	1.393(3)
C9	C10	1.414(3)	C29	C30	1.387(4)
C10	C11	1.378(3)	C30	C31	1.387(4)
C12	C13	1.383(3)	C31	C32	1.388(3)

Table S12 Bond Angles for 2503147lt_auto.

Atom	Atom	Atom	Angle/˚	Atom	Atom	Atom	Angle/˚
C8	N7	C12	126.66(19)	C12	C13	C14	119.3(2)
C11	N7	C8	108.8(2)	C15	C14	C13	120.4(2)
C11	N7	C12	124.39(19)	C14	C15	C16	119.7(2)
C9	N18	C19	128.6(2)	C15	C16	C17	120.2(2)
C2	C1	C11	121.2(2)	C12	C17	C16	119.4(2)
C6	C1	C2	118.9(2)	N18	C19	C24	117.4(2)
C6	C1	C11	119.9(2)	C20	C19	N18	123.2(2)

Table S12 Bond Angles for 2503147lt_auto.

Atom	Atom	Atom	Angle/˚	Atom	Atom	Atom	Angle/˚
C3	C2	C1	120.5(2)	C20	C19	C24	119.4(2)
C2	C3	C4	120.3(3)	C19	C20	C21	119.6(2)
C5	C4	C3	119.7(2)	C22	C21	C20	121.1(2)
C4	C5	C6	120.3(2)	C21	C22	C23	118.9(2)
C5	C6	C1	120.3(3)	C24	C23	C22	120.8(2)
N7	C8	C9	106.32(19)	C23	C24	C19	120.0(2)
N7	C8	C25	128.9(2)	O26	C25	C8	119.3(2)
C9	C8	C25	124.7(2)	O26	C25	C27	118.5(2)
N18	C9	C8	121.7(2)	C8	C25	C27	122.2(2)
N18	C9	C10	130.4(2)	C28	C27	C25	121.9(2)
C8	C9	C10	107.9(2)	C32	C27	C25	118.2(2)
C11	C10	C9	107.1(2)	C32	C27	C28	119.8(2)
N7	C11	C1	122.4(2)	C29	C28	C27	119.5(2)
N7	C11	C10	109.9(2)	C30	C29	C28	120.2(2)
C10	C11	C1	127.6(2)	C31	C30	C29	120.2(2)
C13	C12	N7	120.3(2)	C30	C31	C32	119.6(2)
C13	C12	C17	120.9(2)	C27	C32	C31	120.5(2)
C17	C12	N7	118.8(2)				

Table S13 Torsion Angles for 2503147lt_auto.

A	B	C	D	Angle/˚	A	B	C	D	Angle/˚
O26	C25	C27	C28	133.4(2)	C11	N7	C8	C9	0.0(3)
O26	C25	C27	C32	-44.2(3)	C11	N7	C8	C25	176.4(2)
N7	C8	C9	N18	-177.2(2)	C11	N7	C12	C13	118.3(3)

Table S13 Torsion Angles for 2503147lt_auto.

A	B	C	D	Angle/˚	A	B	C	D	Angle/˚
N7	C8	C9	C10	1.2(3)	C11	N7	C12	C17	-61.7(3)
N7	C8	C25	O26	161.1(2)	C11	C1	C2	C3	-178.7(2)
N7	C8	C25	C27	-20.2(4)	C11	C1	C6	C5	179.4(2)
N7	C12	C13	C14	178.6(2)	C12	N7	C8	C9	175.1(2)
N7	C12	C17	C16	-179.1(2)	C12	N7	C8	C25	-8.5(4)
N18	C9	C10	C11	176.2(2)	C12	N7	C11	C1	-0.9(3)
N18	C19	C20	C21	-177.0(2)	C12	N7	C11	C10	-176.5(2)
N18	C19	C24	C23	177.5(2)	C12	C13	C14	C15	0.7(4)
C1	C2	C3	C4	-0.6(4)	C13	C12	C17	C16	0.9(3)
C2	C1	C6	C5	0.5(4)	C13	C14	C15	C16	0.4(4)
C2	C1	C11	N7	-58.7(3)	C14	C15	C16	C17	-0.9(4)
C2	C1	C11	C10	116.1(3)	C15	C16	C17	C12	0.2(3)
C2	C3	C4	C5	0.4(4)	C17	C12	C13	C14	-1.3(3)
C3	C4	C5	C6	0.2(4)	C19	N18	C9	C8	-163.5(2)
C4	C5	C6	C1	-0.7(4)	C19	N18	C9	C10	18.5(4)
C6	C1	C2	C3	0.1(3)	C19	C20	C21	C22	-1.1(4)
C6	C1	C11	N7	122.5(3)	C20	C19	C24	C23	-3.0(3)
C6	C1	C11	C10	-62.7(3)	C20	C21	C22	C23	-1.8(4)
C8	N7	C11	C1	174.4(2)	C21	C22	C23	C24	2.3(4)
C8	N7	C11	C10	-1.2(3)	C22	C23	C24	C19	0.0(4)
C8	N7	C12	C13	-56.1(3)	C24	C19	C20	C21	3.5(3)
C8	N7	C12	C17	123.8(2)	C25	C8	C9	N18	6.2(4)
C8	C9	C10	C11	-2.0(3)	C25	C8	C9	C10	-175.4(2)
C8	C25	C27	C28	-45.3(4)	C25	C27	C28	C29	-178.3(2)
C8	C25	C27	C32	137.0(2)	C25	C27	C32	C31	-179.1(2)

Table S13 Torsion Angles for 2503147lt_auto.

A	B	C	D	Angle/˚	A	B	C	D	Angle/˚
C9	N18	C19	C20	14.9(4)	C27	C28	C29	C30	-2.3(4)
C9	N18	C19	C24	-165.5(2)	C28	C27	C32	C31	3.2(4)
C9	C8	C25	O26	-23.1(4)	C28	C29	C30	C31	2.8(4)
C9	C8	C25	C27	155.6(2)	C29	C30	C31	C32	-0.3(4)
C9	C10	C11	N7	2.0(3)	C30	C31	C32	C27	-2.8(4)
C9	C10	C11	C1	-173.4(2)	C32	C27	C28	C29	-0.7(4)

Table S14 Hydrogen Atom Coordinates (Å×10^4) and Isotropic Displacement Parameters (Å^2×10^3) for 2503147lt_auto.

Atom	*x*	*y*	*z*	U(eq)
H18	3600(9)	6510(30)	8680(40)	33(8)
H2	4534.4	2418.64	4190.88	34
H3	4988.42	1434.29	1797.8	38
H4	5114.39	2199.44	-1619.89	36
H5	4789.6	3970.34	-2609.94	38
H6	4343.93	4987.28	-196.4	34
H10	4421.55	6066.24	4248.84	29
H13	3634.51	2197.52	7212.99	29
H14	3350.31	342.43	6434.12	33
H15	3132.81	-120.83	2953.02	32
H16	3206.13	1267.55	231.55	31
H17	3481.39	3133.14	1011.11	28
H20	4183.11	7802.68	4346.92	31
H21	4508.93	9655.4	4440.93	35

Table S14 Hydrogen Atom Coordinates (Å×10^4) and Isotropic Displacement Parameters (Å^2×10^3) for 2503147lt_auto.

Atom	*x*	*y*	*z*	U(eq)
H22	4497.74	10771.87	7560.15	38
H23	4124.46	10060.54	10572.29	37
H24	3807.23	8207.34	10534.3	31
H28	2776.78	4315.57	3601.8	30
H29	2167.88	3127.21	2789.6	36
H30	1858.9	1932.89	5421.6	37
H31	2118.13	2027.26	8938.8	34
H32	2690.68	3313.76	9806.89	31

(c) X-ray crystallographic data of compound (6a)

Ellipsoid contour % probability level = 50%

Sample Preparation for Crystal Growth: The compound **6a** was dissolved in Ethyl acetate/Hexane (1:5) and kept for slow evaporation (6 days). A needle-shaped crystal was formed, and its X-ray analysis was performed.

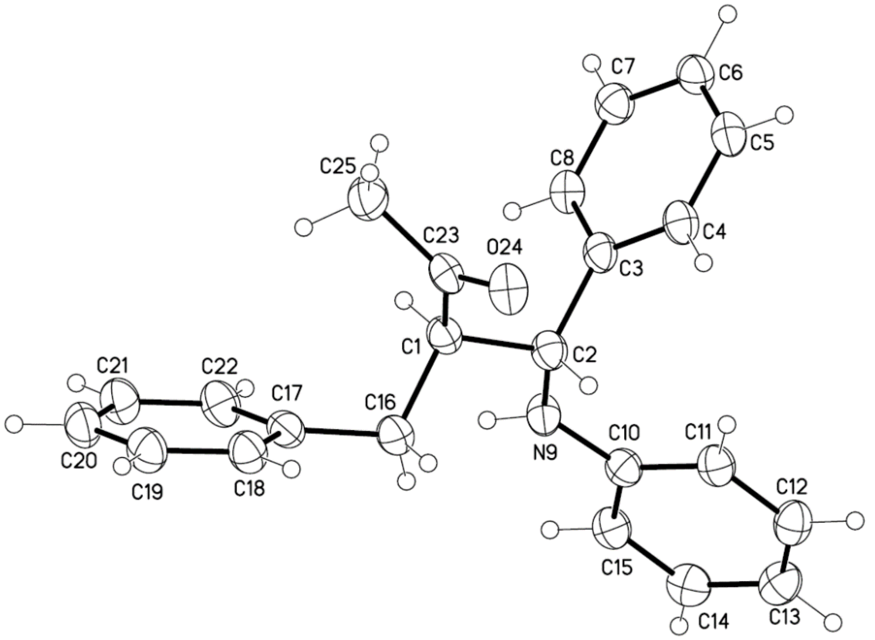

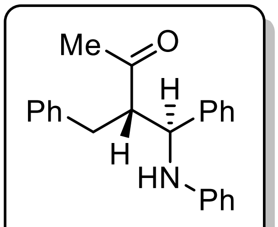

6a
CCDC 2483190

2508043lt2_auto

Table S15 Crystal data and structure refinement for 2508043lt2_auto.

Identification code	2508043lt2_auto
Empirical formula	$C_{23}H_{23}NO$
Formula weight	329.42
Temperature/K	100.00(15)
Crystal system	monoclinic
Space group	$P2_1/c$
a/Å	17.0188(4)
b/Å	5.82210(19)
c/Å	17.9920(6)
α/°	90
β/°	92.976(3)

γ/°	90
Volume/Å^3	1780.33(9)
Z	4
ρ_{calc}g/cm^3	1.229
μ/mm^{-1}	0.575
F(000)	704.0
Crystal size/mm^3	0.16 × 0.02 × 0.01
Radiation	Cu Kα (λ = 1.54184)
2Θ range for data collection/°	5.2 to 146.034
Index ranges	-20 ≤ h ≤ 20, -4 ≤ k ≤ 6, -21 ≤ l ≤ 22
Reflections collected	9787
Independent reflections	3407 [R_{int} = 0.0336, R_{sigma} = 0.0382]
Data/restraints/parameters	3407/0/228
Goodness-of-fit on F^2	1.095
Final R indexes [I>=2σ (I)]	R_1 = 0.0534, wR_2 = 0.1424
Final R indexes [all data]	R_1 = 0.0695, wR_2 = 0.1503
Largest diff. peak/hole / e Å^{-3}	0.29/-0.28

Table S16 Fractional Atomic Coordinates (×10^4) and Equivalent Isotropic Displacement Parameters (Å^2×10^3) for 2508043lt2_auto. U_{eq} is defined as 1/3 of the trace of the orthogonalised U_{IJ} tensor.

Atom	*x*	*y*	*z*	U(eq)
O24	2144.2(8)	6129(2)	1716.3(8)	35.9(4)
N9	2625.0(9)	-77(3)	2885.2(9)	28.7(4)
C1	1992.5(10)	2083(3)	1859.7(11)	27.1(4)
C2	2691.0(10)	1995(3)	2440.8(11)	27.1(4)
C3	3477.4(10)	2195(3)	2077.5(10)	26.6(4)

Table S16 Fractional Atomic Coordinates (×10^4) and Equivalent Isotropic Displacement Parameters (Å^2×10^3) for 2508043lt2_auto. U_{eq} is defined as 1/3 of the trace of the orthogonalised U_{IJ} tensor.

Atom	*x*	*y*	*z*	U(eq)
C4	3949.2(11)	4122(3)	2191.6(11)	29.3(4)
C5	4675.3(11)	4286(3)	1873.5(11)	31.2(5)
C6	4931.6(11)	2522(3)	1428.6(11)	31.1(5)
C7	4462.6(11)	592(3)	1308.7(11)	31.1(4)
C8	3742.9(11)	425(3)	1628.9(11)	29.0(4)
C10	2994.5(10)	-319(3)	3585.6(11)	27.1(4)
C11	3450.3(10)	1414(3)	3931.9(11)	29.3(4)
C12	3782.7(11)	1102(3)	4644.8(11)	33.0(5)
C13	3676.8(12)	-921(4)	5031.4(12)	34.7(5)
C14	3231.4(11)	-2662(4)	4689.5(12)	33.6(5)
C15	2897.2(11)	-2376(3)	3976.5(11)	29.8(4)
C16	1197.2(11)	1978(4)	2240.2(11)	31.9(5)
C17	488.8(10)	1546(3)	1715.6(11)	29.1(4)
C18	-100.2(11)	3189(4)	1611.9(12)	32.5(5)
C19	-764.7(11)	2757(4)	1151.3(12)	35.5(5)
C20	-846.5(11)	677(4)	786.3(12)	36.1(5)
C21	-260.0(11)	-968(4)	879.1(12)	35.7(5)
C22	399.1(11)	-541(3)	1343.2(12)	33.6(5)
C23	2004.5(10)	4285(3)	1409.8(11)	28.2(4)
C25	1801.6(13)	4133(4)	594.2(12)	38.1(5)

Table S17 Anisotropic Displacement Parameters (Å^2×10^3) for 2508043lt2_auto. The Anisotropic displacement factor exponent takes the form: $-2\pi^2[h^2a^{*2}U_{11}+2hka^*b^*U_{12}+…]$.

Atom	U_{11}	U_{22}	U_{33}	U_{23}	U_{13}	U_{12}
O24	33.0(7)	27.9(8)	45.8(9)	-1.5(6)	-7.0(6)	1.3(6)
N9	26.7(8)	27.9(9)	30.9(9)	1.7(7)	-4.6(7)	-3.8(6)
C1	22.2(9)	29.3(10)	29.9(10)	-0.5(8)	1.1(7)	-0.6(7)
C2	25.7(9)	25.9(10)	29.2(10)	2.1(8)	-1.4(8)	-0.3(7)
C3	22.6(9)	28.5(10)	28.1(10)	3.2(7)	-4.5(7)	0.9(7)
C4	25.5(9)	27.1(10)	34.6(11)	1.6(8)	-4.2(8)	0.2(7)
C5	27.5(9)	29.5(11)	35.8(11)	1.1(8)	-4.5(8)	-4.3(8)
C6	24.7(9)	37.8(11)	30.6(10)	3.7(8)	-0.8(8)	-0.9(8)
C7	28.9(10)	32.3(11)	31.8(10)	0.8(8)	-0.5(8)	2.0(8)
C8	27.3(9)	27.3(10)	31.9(10)	-1.9(8)	-3.7(8)	-1.5(8)
C10	21.8(8)	29.5(10)	30.0(10)	-0.1(8)	0.7(7)	3.9(7)
C11	25.7(9)	28.1(10)	34.0(10)	-0.8(8)	-0.2(8)	2.5(7)
C12	31.2(10)	32.7(11)	34.6(11)	-4.7(9)	-3.5(8)	3.8(8)
C13	35.5(11)	36.3(12)	31.7(11)	1.3(9)	-1.8(9)	7.8(9)
C14	31.9(10)	32.0(11)	37.1(11)	3.6(9)	2.6(8)	5.6(8)
C15	26.3(9)	28.4(10)	34.7(11)	0.3(8)	-0.2(8)	2.5(8)
C16	24.2(9)	39.5(11)	31.9(10)	1.4(9)	0.2(8)	-0.8(8)
C17	24.2(9)	32.5(11)	30.7(10)	3.2(8)	2.5(8)	-2.1(8)
C18	27.6(10)	32.3(11)	37.7(11)	-3.8(9)	1.6(8)	0.4(8)
C19	25.8(9)	39.9(12)	40.2(12)	0.3(9)	-2.1(8)	5.5(8)
C20	26.3(10)	42.0(12)	39.6(12)	-4.4(9)	-2.8(9)	-2.1(8)
C21	31.0(10)	33.3(11)	42.7(12)	-5.2(9)	0.2(9)	-1.8(8)
C22	27.2(10)	29.5(11)	43.9(12)	1.9(9)	-1.7(8)	1.5(8)
C23	18.8(8)	30.1(11)	35.4(11)	1.3(8)	-0.6(7)	-0.3(7)

Table S17 Anisotropic Displacement Parameters (Å^2×10^3) for 2508043lt2_auto. The Anisotropic displacement factor exponent takes the form: $-2\pi^2[h^2a^{*2}U_{11}+2hka^*b^*U_{12}+\ldots]$.

Atom	U_{11}	U_{22}	U_{33}	U_{23}	U_{13}	U_{12}
C25	39.3(11)	38.4(12)	36.1(12)	4.0(9)	-3.3(9)	-8.0(9)

Table S18 Bond Lengths for 2508043lt2_auto.

Atom	Atom	Length/Å	Atom	Atom	Length/Å
O24	C23	1.225(2)	C10	C15	1.403(3)
N9	C2	1.455(2)	C11	C12	1.387(3)
N9	C10	1.386(2)	C12	C13	1.384(3)
C1	C2	1.543(2)	C13	C14	1.390(3)
C1	C16	1.549(3)	C14	C15	1.386(3)
C1	C23	1.517(3)	C16	C17	1.513(2)
C2	C3	1.524(3)	C17	C18	1.391(3)
C3	C4	1.388(3)	C17	C22	1.392(3)
C3	C8	1.399(3)	C18	C19	1.390(3)
C4	C5	1.392(3)	C19	C20	1.381(3)
C5	C6	1.387(3)	C20	C21	1.387(3)
C6	C7	1.388(3)	C21	C22	1.386(3)
C7	C8	1.384(3)	C23	C25	1.493(3)
C10	C11	1.399(3)			

Table S19 Bond Angles for 2508043lt2_auto.

Atom	Atom	Atom	Angle/˚	Atom	Atom	Atom	Angle/˚
C10	N9	C2	122.67(15)	C12	C11	C10	120.51(18)

Table S19 Bond Angles for 2508043lt2_auto.

Atom	Atom	Atom	Angle/˚	Atom	Atom	Atom	Angle/˚
C2	C1	C16	111.06(15)	C13	C12	C11	121.25(18)
C23	C1	C2	111.02(15)	C12	C13	C14	118.70(18)
C23	C1	C16	107.78(15)	C15	C14	C13	120.68(19)
N9	C2	C1	108.75(14)	C14	C15	C10	120.86(18)
N9	C2	C3	113.20(15)	C17	C16	C1	114.65(16)
C3	C2	C1	111.70(15)	C18	C17	C16	120.95(18)
C4	C3	C2	120.84(17)	C18	C17	C22	118.39(17)
C4	C3	C8	118.62(17)	C22	C17	C16	120.62(17)
C8	C3	C2	120.54(16)	C19	C18	C17	120.91(19)
C3	C4	C5	120.91(18)	C20	C19	C18	120.08(19)
C6	C5	C4	119.94(18)	C19	C20	C21	119.60(18)
C5	C6	C7	119.58(18)	C22	C21	C20	120.23(19)
C8	C7	C6	120.42(19)	C21	C22	C17	120.78(18)
C7	C8	C3	120.52(18)	O24	C23	C1	120.55(17)
N9	C10	C11	123.13(17)	O24	C23	C25	121.61(18)
N9	C10	C15	118.86(17)	C25	C23	C1	117.77(16)
C11	C10	C15	118.00(17)				

Table S20 Torsion Angles for 2508043lt2_auto.

A	B	C	D	Angle/˚	A	B	C	D	Angle/˚
N9	C2	C3	C4	-124.51(18)	C10	N9	C2	C3	76.4(2)
N9	C2	C3	C8	54.7(2)	C10	C11	C12	C13	0.2(3)
N9	C10	C11	C12	177.67(17)	C11	C10	C15	C14	1.2(3)

Table S20 Torsion Angles for 2508043lt2_auto.

A	B	C	D	Angle/˚	A	B	C	D	Angle/˚
N9	C10	C15	C14	-177.58(17)	C11	C12	C13	C14	0.4(3)
C1	C2	C3	C4	112.33(19)	C12	C13	C14	C15	-0.3(3)
C1	C2	C3	C8	-68.5(2)	C13	C14	C15	C10	-0.5(3)
C1	C16	C17	C18	-115.8(2)	C15	C10	C11	C12	-1.0(3)
C1	C16	C17	C22	66.3(2)	C16	C1	C2	N9	56.7(2)
C2	N9	C10	C11	1.2(3)	C16	C1	C2	C3	-177.65(16)
C2	N9	C10	C15	179.84(16)	C16	C1	C23	O24	78.3(2)
C2	C1	C16	C17	-167.57(16)	C16	C1	C23	C25	-98.66(19)
C2	C1	C23	O24	-43.5(2)	C16	C17	C18	C19	-177.59(19)
C2	C1	C23	C25	139.50(18)	C16	C17	C22	C21	178.11(19)
C2	C3	C4	C5	178.58(17)	C17	C18	C19	C20	-0.3(3)
C2	C3	C8	C7	-178.97(17)	C18	C17	C22	C21	0.2(3)
C3	C4	C5	C6	0.8(3)	C18	C19	C20	C21	-0.4(3)
C4	C3	C8	C7	0.3(3)	C19	C20	C21	C22	0.9(3)
C4	C5	C6	C7	-0.5(3)	C20	C21	C22	C17	-0.8(3)
C5	C6	C7	C8	0.1(3)	C22	C17	C18	C19	0.4(3)
C6	C7	C8	C3	0.0(3)	C23	C1	C2	N9	176.60(15)
C8	C3	C4	C5	-0.6(3)	C23	C1	C2	C3	-57.8(2)
C10	N9	C2	C1	-158.84(17)	C23	C1	C16	C17	70.6(2)

Table S21 Hydrogen Atom Coordinates (Å×10^4) and Isotropic Displacement Parameters (Å^2×10^3) for 2508043lt2_auto.

Atom	*x*	*y*	*z*	U(eq)
H9	2338.87	-1222.95	2701.99	34
H1	2027.78	743.73	1515.07	33
H2	2642.96	3341.41	2780.37	32
H4	3774.05	5344.72	2490.93	35
H5	4995.04	5605.8	1961.1	37
H6	5424.94	2632.27	1207.16	37
H7	4636.99	-621.2	1004.93	37
H8	3426.62	-903.05	1543.24	35
H11	3532.76	2814.42	3676.63	35
H12	4088.9	2298.74	4872.12	40
H13	3903.94	-1115.17	5520.9	42
H14	3155.4	-4062.31	4947.12	40
H15	2598.37	-3589.25	3749.55	36
H16A	1226.46	744.62	2619.26	38
H16B	1118.92	3448.57	2502.71	38
H18	-47.53	4626.62	1859.39	39
H19	-1163.06	3893.22	1087.4	43
H20	-1301.68	375.02	473.47	43
H21	-310.38	-2392.86	623.65	43
H22	794.54	-1685.78	1408	40
H25A	1727.94	5683.08	389.67	57
H25B	1314.39	3252.31	510.2	57
H25C	2229.1	3365.26	347.26	57

Experimental

Single crystals of $C_{23}H_{23}NO$ **[2508043lt2_auto]** were **[]**. A suitable crystal was selected and **[]** on a **XtaLAB Synergy R, DW system, HyPix-Arc 150** diffractometer. The crystal was kept at 100.00(15) K during data collection. Using Olex2 [1], the structure was solved with the SHELXT [2] structure solution program using Intrinsic Phasing and refined with the SHELXL [3] refinement package using Least Squares minimisation.

1. Dolomanov, O.V., Bourhis, L.J., Gildea, R.J, Howard, J.A.K. & Puschmann, H. (2009), J. Appl. Cryst. 42, 339-341.
2. Sheldrick, G.M. (2015). Acta Cryst. A71, 3-8.
3. Sheldrick, G.M. (2015). Acta Cryst. C71, 3-8.

Crystal structure determination of [2508043lt2_auto]

Crystal Data for $C_{23}H_{23}NO$ (M =329.42 g/mol): monoclinic, space group P2$_1$/c (no. 14), a = 17.0188(4) Å, b = 5.82210(19) Å, c = 17.9920(6) Å, β = 92.976(3)°, V = 1780.33(9) Å^3, Z = 4, T = 100.00(15) K, μ(Cu Kα) = 0.575 mm^{-1}, $Dcalc$ = 1.229 g/cm^3, 9787 reflections measured (5.2° ≤ 2Θ ≤ 146.034°), 3407 unique (R_{int} = 0.0336, R_{sigma} = 0.0382) which were used in all calculations. The final R_1 was 0.0534 (I > 2σ(I)) and wR_2 was 0.1503 (all data).

Refinement model description

Number of restraints - 0, number of constraints - unknown.

Details:

```
1. Fixed Uiso

 At 1.2 times of:

  All C(H) groups, All C(H,H) groups, All N(H) groups

 At 1.5 times of:

  All C(H,H,H) groups

2.a Ternary CH refined with riding coordinates:

 C1(H1), C2(H2)

2.b Secondary CH2 refined with riding coordinates:

 C16(H16A,H16B)

2.c Aromatic/amide H refined with riding coordinates:

 N9(H9), C4(H4), C5(H5), C6(H6), C7(H7), C8(H8), C11(H11), C12(H12), C13(H13),

 C14(H14), C15(H15), C18(H18), C19(H19), C20(H20), C21(H21), C22(H22)

2.d Idealised Me refined as rotating group:

 C25(H25A,H25B,H25C)
```

(d) X-ray crystallographic data of compound (6b)

Ellipsoid contour % probability level = 50%

Sample Preparation for Crystal Growth: The compound **6b** was dissolved in tetrahydofuran and kept for slow evaporation (4 days). A rod-shaped crystal was formed, and its X-ray analysis was performed.

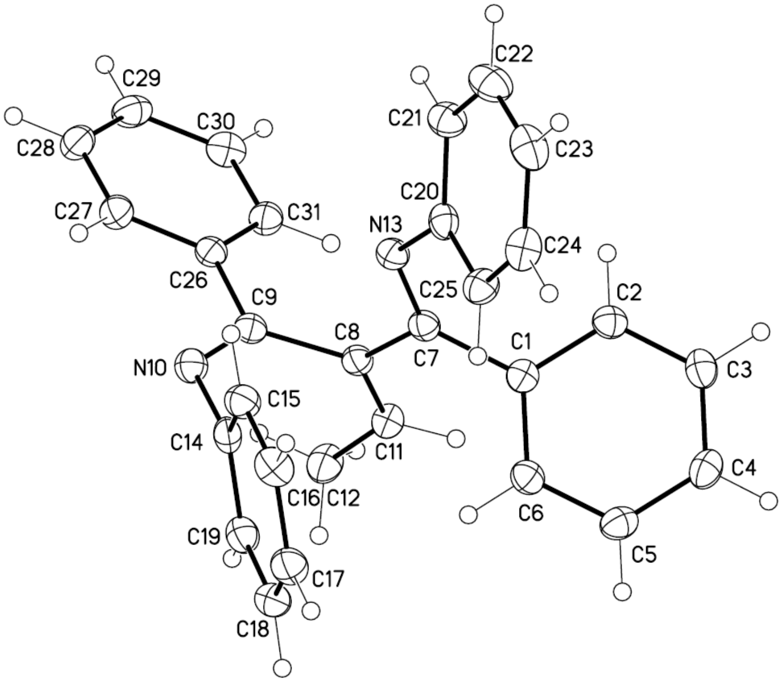

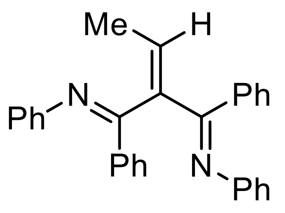

6b
CCDC 2483359

2508082lt2

Table S22 Crystal data and structure refinement for 2508082lt2.

Identification code	2508082lt2
Empirical formula	$C_{29}H_{24}N_2$
Formula weight	400.50
Temperature/K	100.00(10)
Crystal system	triclinic

Space group	P-1
a/Å	9.8243(3)
b/Å	10.0260(3)
c/Å	12.1866(4)
α/°	106.834(3)
β/°	105.750(3)
γ/°	97.612(2)
Volume/Å^3	1076.37(6)
Z	2
ρ_{calc}g/cm^3	1.236
μ/mm^{-1}	0.552
F(000)	424.0
Crystal size/mm^3	0.13 × 0.07 × 0.04
Radiation	Cu Kα (λ = 1.54184)
2Θ range for data collection/°	8.02 to 134.096
Index ranges	-11 ≤ h ≤ 11, -11 ≤ k ≤ 11, -14 ≤ l ≤ 14
Reflections collected	12117
Independent reflections	3786 [R_{int} = 0.0239, R_{sigma} = 0.0271]
Data/restraints/parameters	3786/0/281
Goodness-of-fit on F^2	1.059
Final R indexes [I>=2σ (I)]	R_1 = 0.0357, wR_2 = 0.0871
Final R indexes [all data]	R_1 = 0.0412, wR_2 = 0.0903
Largest diff. peak/hole / e Å^{-3}	0.16/-0.20

Table 23 Fractional Atomic Coordinates (×10[4]) and Equivalent Isotropic Displacement Parameters ($Å^2×10^3$) for 2508082lt2. U_{eq} is defined as 1/3 of the trace of the orthogonalised U_{IJ} tensor.

Atom	*x*	*y*	*z*	U(eq)
N10	7674.0(11)	9476.3(10)	4786.5(9)	21.5(2)
N13	5153.4(11)	6646.9(10)	4004.6(9)	20.1(2)
C1	6270.6(12)	4699.5(12)	3145.0(10)	19.0(3)
C2	5517.8(13)	3463.9(13)	3207.3(11)	21.1(3)
C3	5509.9(13)	2125.6(13)	2448.1(11)	24.2(3)
C4	6239.3(13)	2016.6(13)	1613.4(11)	25.1(3)
C5	6974.2(13)	3248.0(13)	1530.2(11)	24.3(3)
C6	7002.5(13)	4585.3(13)	2296.5(10)	21.6(3)
C7	6283.9(13)	6139.4(12)	3969.6(10)	18.6(3)
C8	7706.8(13)	7058.1(12)	4819.8(10)	18.5(3)
C9	7738.1(12)	8617.2(12)	5387.4(10)	18.6(3)
C11	8920.1(13)	6565.9(13)	5031.1(11)	21.4(3)
C12	10407.0(14)	7422.1(13)	5820.9(12)	26.8(3)
C14	7465.6(13)	8942.3(12)	3521.6(11)	21.1(3)
C15	6089.6(14)	8775.3(13)	2719.8(11)	24.2(3)
C16	5843.0(14)	8311.5(13)	1483.3(11)	27.0(3)
C17	6964.9(14)	8036.3(13)	1027.3(12)	26.9(3)
C18	8343.3(14)	8226.4(13)	1826.9(12)	26.6(3)
C19	8599.6(14)	8670.0(13)	3071.0(11)	24.1(3)
C20	3752.8(13)	5958.3(12)	3146.8(11)	20.9(3)
C21	2576.0(14)	5962.5(13)	3577.9(12)	25.6(3)
C22	1166.5(14)	5382.9(15)	2778.9(13)	30.7(3)
C23	911.1(14)	4819.5(14)	1542.4(13)	30.3(3)
C24	2074.2(14)	4846.3(13)	1106.9(12)	27.0(3)

Table 23 Fractional Atomic Coordinates (×10[4]) and Equivalent Isotropic Displacement Parameters (Å[2]×10[3]) for 2508082lt2. U_{eq} is defined as 1/3 of the trace of the orthogonalised U_{IJ} tensor.

Atom	*x*	*y*	*z*	U(eq)
C25	3489.0(14)	5409.9(13)	1895.7(11)	24.0(3)
C26	7870.4(12)	9199.6(12)	6694.8(10)	19.2(3)
C27	7696.1(13)	10588.8(13)	7175.1(11)	23.0(3)
C28	7850.3(14)	11161.1(13)	8394.2(11)	25.9(3)
C29	8182.4(14)	10366.0(14)	9153.4(11)	26.0(3)
C30	8340.7(13)	8985.1(14)	8680.6(11)	24.9(3)
C31	8174.7(13)	8399.1(13)	7458.2(11)	22.1(3)

Table 24 Anisotropic Displacement Parameters (Å[2]×10[3]) for 2508082lt2. The Anisotropic displacement factor exponent takes the form: $-2\pi^2[h^2a^{*2}U_{11}+2hka^*b^*U_{12}+\ldots]$.

Atom	U_{11}	U_{22}	U_{33}	U_{23}	U_{13}	U_{12}
N10	21.1(5)	20.8(5)	21.5(5)	6.8(4)	6.0(4)	4.4(4)
N13	18.0(5)	21.2(5)	21.3(5)	7.4(4)	6.6(4)	4.0(4)
C1	16.0(6)	20.2(6)	17.9(6)	5.4(5)	2.4(5)	3.7(5)
C2	17.8(6)	23.6(6)	20.8(6)	7.0(5)	5.2(5)	3.7(5)
C3	21.4(6)	19.6(6)	28.3(7)	7.8(5)	4.3(5)	2.4(5)
C4	23.9(6)	21.0(6)	24.0(7)	1.9(5)	4.0(5)	5.5(5)
C5	21.7(6)	28.0(7)	20.6(6)	4.2(5)	7.2(5)	5.7(5)
C6	18.9(6)	22.2(6)	21.3(6)	6.8(5)	4.7(5)	2.2(5)
C7	20.2(6)	19.4(6)	17.1(6)	7.3(5)	6.6(5)	3.8(5)
C8	19.9(6)	18.9(6)	16.1(6)	5.6(5)	6.3(5)	2.9(5)
C9	11.9(5)	20.1(6)	22.1(6)	6.2(5)	4.6(4)	2.8(4)
C11	22.5(6)	19.6(6)	21.2(6)	5.7(5)	6.7(5)	5.3(5)
C12	21.3(6)	25.0(6)	29.3(7)	4.7(5)	4.8(5)	6.4(5)

Table 24 Anisotropic Displacement Parameters (Å^2×10^3) for 2508082lt2. The Anisotropic displacement factor exponent takes the form: $-2\pi^2[h^2a^{*2}U_{11}+2hka^*b^*U_{12}+…]$.

Atom	U_{11}	U_{22}	U_{33}	U_{23}	U_{13}	U_{12}
C14	25.4(6)	14.8(6)	21.3(6)	5.9(5)	6.5(5)	1.8(5)
C15	24.6(6)	25.0(6)	25.9(7)	10.8(5)	10.2(5)	6.2(5)
C16	26.2(7)	29.8(7)	24.2(7)	12.1(5)	4.8(5)	4.1(5)
C17	31.7(7)	27.0(7)	20.7(6)	7.8(5)	9.0(5)	2.2(5)
C18	27.2(7)	25.7(7)	27.1(7)	7.5(5)	12.5(5)	2.8(5)
C19	22.1(6)	21.4(6)	26.4(7)	7.5(5)	6.5(5)	1.9(5)
C20	19.8(6)	16.6(6)	25.3(6)	7.8(5)	4.9(5)	4.8(5)
C21	22.2(6)	28.8(7)	28.4(7)	12.4(5)	8.7(5)	7.1(5)
C22	19.7(6)	37.4(8)	39.4(8)	19.1(6)	9.7(6)	6.8(6)
C23	20.9(6)	28.5(7)	37.2(8)	14.7(6)	0.2(6)	3.2(5)
C24	27.5(7)	24.5(6)	24.3(7)	7.2(5)	1.5(5)	7.4(5)
C25	22.1(6)	24.6(6)	25.5(7)	8.1(5)	7.0(5)	8.1(5)
C26	13.9(5)	19.9(6)	21.6(6)	4.9(5)	5.5(5)	2.1(5)
C27	22.0(6)	21.8(6)	25.4(7)	7.3(5)	8.5(5)	5.6(5)
C28	24.4(7)	22.7(6)	27.7(7)	2.4(5)	10.7(5)	5.2(5)
C29	22.8(6)	31.5(7)	20.2(6)	3.2(5)	8.6(5)	4.1(5)
C30	21.5(6)	30.4(7)	22.5(6)	10.1(5)	6.0(5)	5.1(5)
C31	19.7(6)	21.8(6)	24.2(6)	6.7(5)	7.4(5)	4.9(5)

Table 25 Bond Lengths for 2508082lt2.

Atom	Atom	Length/Å	Atom	Atom	Length/Å
N10	C9	1.2793(15)	C14	C19	1.3940(17)
N10	C14	1.4236(15)	C15	C16	1.3827(17)

Table 25 Bond Lengths for 2508082lt2.

Atom	Atom	Length/Å	Atom	Atom	Length/Å
N13	C7	1.2873(15)	C16	C17	1.3872(18)
N13	C20	1.4185(15)	C17	C18	1.3883(18)
C1	C2	1.3891(17)	C18	C19	1.3901(17)
C1	C6	1.3986(16)	C20	C21	1.3937(17)
C1	C7	1.4972(16)	C20	C25	1.3987(17)
C2	C3	1.3896(17)	C21	C22	1.3894(18)
C3	C4	1.3828(18)	C22	C23	1.3846(19)
C4	C5	1.3900(18)	C23	C24	1.3847(19)
C5	C6	1.3861(17)	C24	C25	1.3882(17)
C7	C8	1.4818(16)	C26	C27	1.3990(17)
C8	C9	1.5074(16)	C26	C31	1.3943(17)
C8	C11	1.3392(17)	C27	C28	1.3857(17)
C9	C26	1.4917(16)	C28	C29	1.3894(19)
C11	C12	1.4910(17)	C29	C30	1.3844(18)
C14	C15	1.3922(17)	C30	C31	1.3881(17)

Table 26 Bond Angles for 2508082lt2.

Atom	Atom	Atom	Angle/˚	Atom	Atom	Atom	Angle/˚
C9	N10	C14	119.74(10)	C19	C14	N10	122.45(11)
C7	N13	C20	122.71(10)	C16	C15	C14	120.16(12)
C2	C1	C6	119.36(11)	C15	C16	C17	120.62(12)
C2	C1	C7	120.25(11)	C16	C17	C18	119.26(12)
C6	C1	C7	120.39(10)	C17	C18	C19	120.66(12)
C1	C2	C3	120.40(12)	C18	C19	C14	119.70(12)

Table 26 Bond Angles for 2508082lt2.

Atom	Atom	Atom	Angle/˚	Atom	Atom	Atom	Angle/˚
C4	C3	C2	120.07(12)	C21	C20	N13	117.35(11)
C3	C4	C5	119.91(11)	C21	C20	C25	118.95(11)
C6	C5	C4	120.27(12)	C25	C20	N13	123.39(11)
C5	C6	C1	119.97(11)	C22	C21	C20	120.41(12)
N13	C7	C1	125.22(10)	C23	C22	C21	120.43(12)
N13	C7	C8	117.01(10)	C22	C23	C24	119.37(12)
C8	C7	C1	117.78(10)	C23	C24	C25	120.80(12)
C7	C8	C9	116.07(10)	C24	C25	C20	119.99(12)
C11	C8	C7	122.66(10)	C27	C26	C9	119.41(11)
C11	C8	C9	121.17(10)	C31	C26	C9	121.74(10)
N10	C9	C8	122.08(10)	C31	C26	C27	118.85(11)
N10	C9	C26	118.22(10)	C28	C27	C26	120.24(12)
C26	C9	C8	119.69(10)	C27	C28	C29	120.51(12)
C8	C11	C12	126.42(11)	C30	C29	C28	119.51(12)
C15	C14	N10	117.86(11)	C29	C30	C31	120.34(12)
C15	C14	C19	119.58(11)	C30	C31	C26	120.52(11)

Table 27 Torsion Angles for 2508082lt2.

A	B	C	D	Angle/˚	A	B	C	D	Angle/˚
N10	C9	C26	C27	9.78(16)	C9	N10	C14	C15	103.50(13)
N10	C9	C26	C31	-169.75(11)	C9	N10	C14	C19	-80.48(15)
N10	C14	C15	C16	177.57(11)	C9	C8	C11	C12	0.50(19)
N10	C14	C19	C18	-176.36(11)	C9	C26	C27	C28	-178.41(10)

Table 27 Torsion Angles for 2508082lt2.

A	B	C	D	Angle/˚	A	B	C	D	Angle/˚
N13	C7	C8	C9	-15.99(15)	C9	C26	C31	C30	177.80(11)
N13	C7	C8	C11	167.51(12)	C11	C8	C9	N10	103.28(14)
N13	C20	C21	C22	176.17(11)	C11	C8	C9	C26	-75.76(15)
N13	C20	C25	C24	-175.17(11)	C14	N10	C9	C8	4.46(17)
C1	C2	C3	C4	-0.78(18)	C14	N10	C9	C26	-176.49(10)
C1	C7	C8	C9	164.25(10)	C14	C15	C16	C17	-1.36(19)
C1	C7	C8	C11	-12.25(17)	C15	C14	C19	C18	-0.40(18)
C2	C1	C6	C5	-0.13(17)	C15	C16	C17	C18	0.24(19)
C2	C1	C7	N13	-60.74(17)	C16	C17	C18	C19	0.81(19)
C2	C1	C7	C8	119.00(12)	C17	C18	C19	C14	-0.72(18)
C2	C3	C4	C5	-0.35(19)	C19	C14	C15	C16	1.43(18)
C3	C4	C5	C6	1.23(19)	C20	N13	C7	C1	-8.03(18)
C4	C5	C6	C1	-0.99(18)	C20	N13	C7	C8	172.22(10)
C6	C1	C2	C3	1.02(18)	C20	C21	C22	C23	-1.3(2)
C6	C1	C7	N13	118.59(13)	C21	C20	C25	C24	-1.71(18)
C6	C1	C7	C8	-61.67(15)	C21	C22	C23	C24	-0.4(2)
C7	N13	C20	C21	140.59(12)	C22	C23	C24	C25	0.99(19)
C7	N13	C20	C25	-45.85(17)	C23	C24	C25	C20	0.06(19)
C7	C1	C2	C3	-179.64(11)	C25	C20	C21	C22	2.32(18)
C7	C1	C6	C5	-179.47(11)	C26	C27	C28	C29	0.15(19)
C7	C8	C9	N10	-73.28(14)	C27	C26	C31	C30	-1.73(18)
C7	C8	C9	C26	107.68(12)	C27	C28	C29	C30	-0.84(19)

Table 27 Torsion Angles for 2508082lt2.

A	B	C	D	Angle/˚	A	B	C	D	Angle/˚
C7	C8	C11	C12	176.83(11)	C28	C29	C30	C31	0.24(19)
C8	C9	C26	C27	-171.14(10)	C29	C30	C31	C26	1.06(19)
C8	C9	C26	C31	9.33(17)	C31	C26	C27	C28	1.13(18)

Table 28 Hydrogen Atom Coordinates (Å×10^4) and Isotropic Displacement Parameters (Å^2×10^3) for 2508082lt2.

Atom	*x*	*y*	*z*	U(eq)
H2	5005.22	3534.38	3772.53	25
H3	5002.82	1284.6	2502.46	29
H4	6238.04	1101.31	1097.49	30
H5	7459.05	3172.84	945.72	29
H6	7518.84	5423.92	2245.4	26
H11	8823.9	5573.46	4639.2	26
H12A	11025.56	7477.72	5318.29	40
H12B	10820.32	6961.81	6400.95	40
H12C	10352.34	8389.53	6262.42	40
H15	5318.19	8980.43	3023.05	29
H16	4896.36	8180.17	940.96	32
H17	6792.02	7721.25	177.22	32
H18	9119.02	8051.59	1520.85	32
H19	9543.48	8787.04	3611.8	29
H21	2738.66	6364.2	4423.35	31
H22	372.81	5372.98	3083.09	37
H23	-53.03	4418.5	997.85	36

Table 28 Hydrogen Atom Coordinates (Å×10[4]) and Isotropic Displacement Parameters (Å[2]×10[3]) for 2508082lt2.

Atom	*x*	*y*	*z*	U(eq)
H24	1901.96	4474.45	258.12	32
H25	4277.81	5423.01	1585.76	29
H27	7471.2	11141.77	6663.72	28
H28	7728.05	12104.4	8713.2	31
H29	8299.96	10766.95	9990.85	31
H30	8564.09	8435.79	9195.45	30
H31	8269.27	7445.02	7139.19	27

Experimental

Single crystals of $C_{29}H_{24}N_2$ **[2508082lt2]** were **[]**. A suitable crystal was selected and **[]** on a **XtaLAB Synergy R, DW system, HyPix-Arc 150** diffractometer. The crystal was kept at 100.00(10) K during data collection. Using Olex2 [1], the structure was solved with the SHELXT [2] structure solution program using Intrinsic Phasing and refined with the SHELXL [3] refinement package using Least Squares minimisation.

1. Dolomanov, O.V., Bourhis, L.J., Gildea, R.J, Howard, J.A.K. & Puschmann, H. (2009), J. Appl. Cryst. 42, 339-341.
2. Sheldrick, G.M. (2015). Acta Cryst. A71, 3-8.
3. Sheldrick, G.M. (2015). Acta Cryst. C71, 3-8.

Crystal structure determination of [2508082lt2]

Crystal Data for $C_{29}H_{24}N_2$ (M =400.50 g/mol): triclinic, space group P-1 (no. 2), a = 9.8243(3) Å, b = 10.0260(3) Å, c = 12.1866(4) Å, α = 106.834(3)°, β = 105.750(3)°, γ = 97.612(2)°, V = 1076.37(6) Å^3, Z = 2, T = 100.00(10) K, μ(Cu Kα) = 0.552 mm^{-1}, $Dcalc$ = 1.236 g/cm^3, 12117 reflections measured (8.02° ≤ 2Θ ≤ 134.096°), 3786 unique (R_{int} = 0.0239, R_{sigma} = 0.0271) which were used in all calculations. The final R_1 was 0.0357 (I > 2σ(I)) and wR_2 was 0.0903 (all data).

Refinement model description

Number of restraints - 0, number of constraints - unknown.

Details:

1. Fixed Uiso
 At 1.2 times of:
  All C(H) groups
 At 1.5 times of:
  All C(H,H,H) groups
2.a Aromatic/amide H refined with riding coordinates:
 C2(H2), C3(H3), C4(H4), C5(H5), C6(H6), C11(H11), C15(H15), C16(H16),
 C17(H17), C18(H18), C19(H19), C21(H21), C22(H22), C23(H23), C24(H24), C25(H25),
 C27(H27), C28(H28), C29(H29), C30(H30), C31(H31)
2.b Idealised Me refined as rotating group:
 C12(H12A,H12B,H12C)

7. ^{1}H,^{13}C spectra of key compounds

^{1}H-NMR (500 MHz, $CDCl_3$)

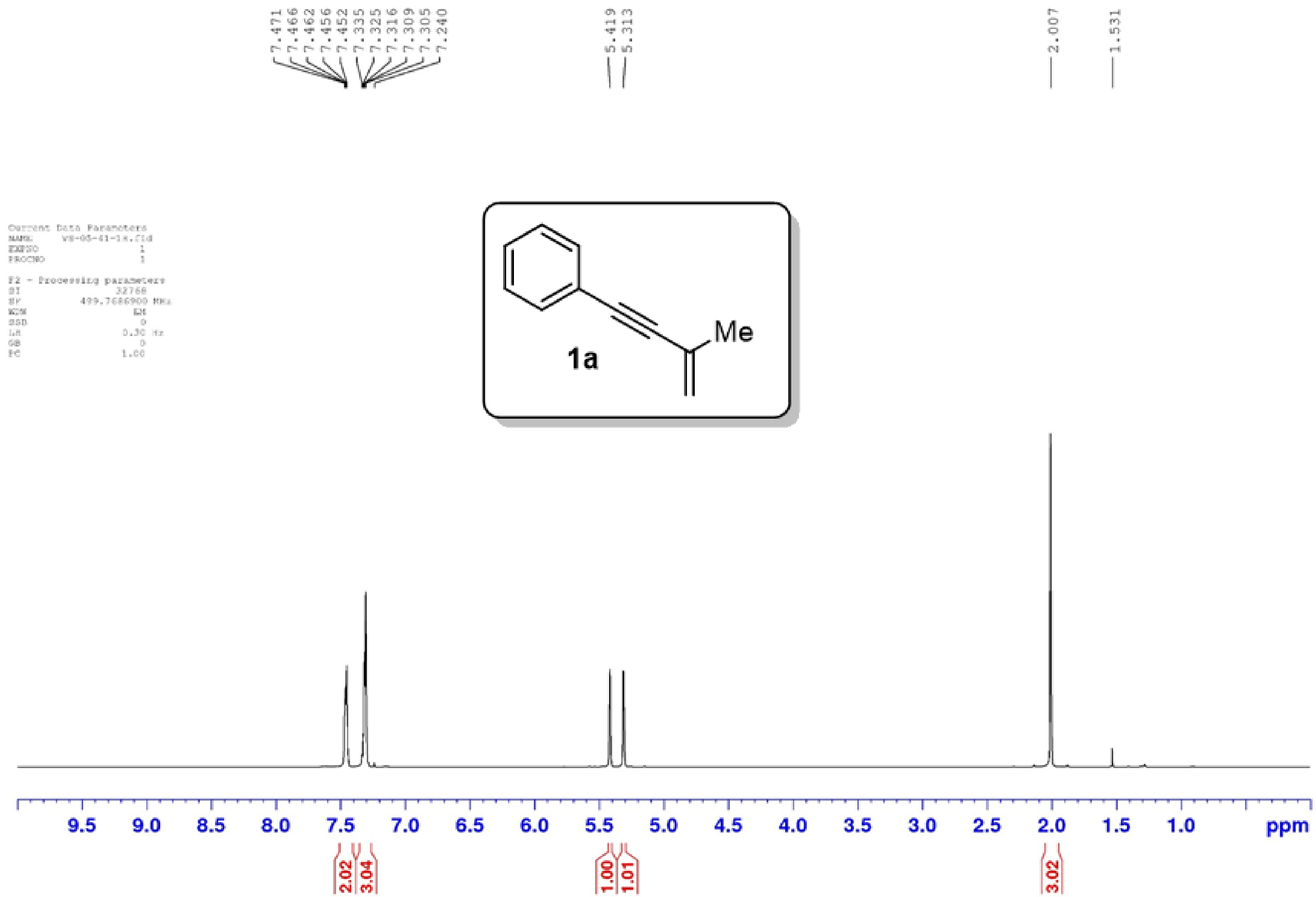

$^{13}C\{1H\}$ and DEPT NMR (125 MHz, $CDCl_3$)

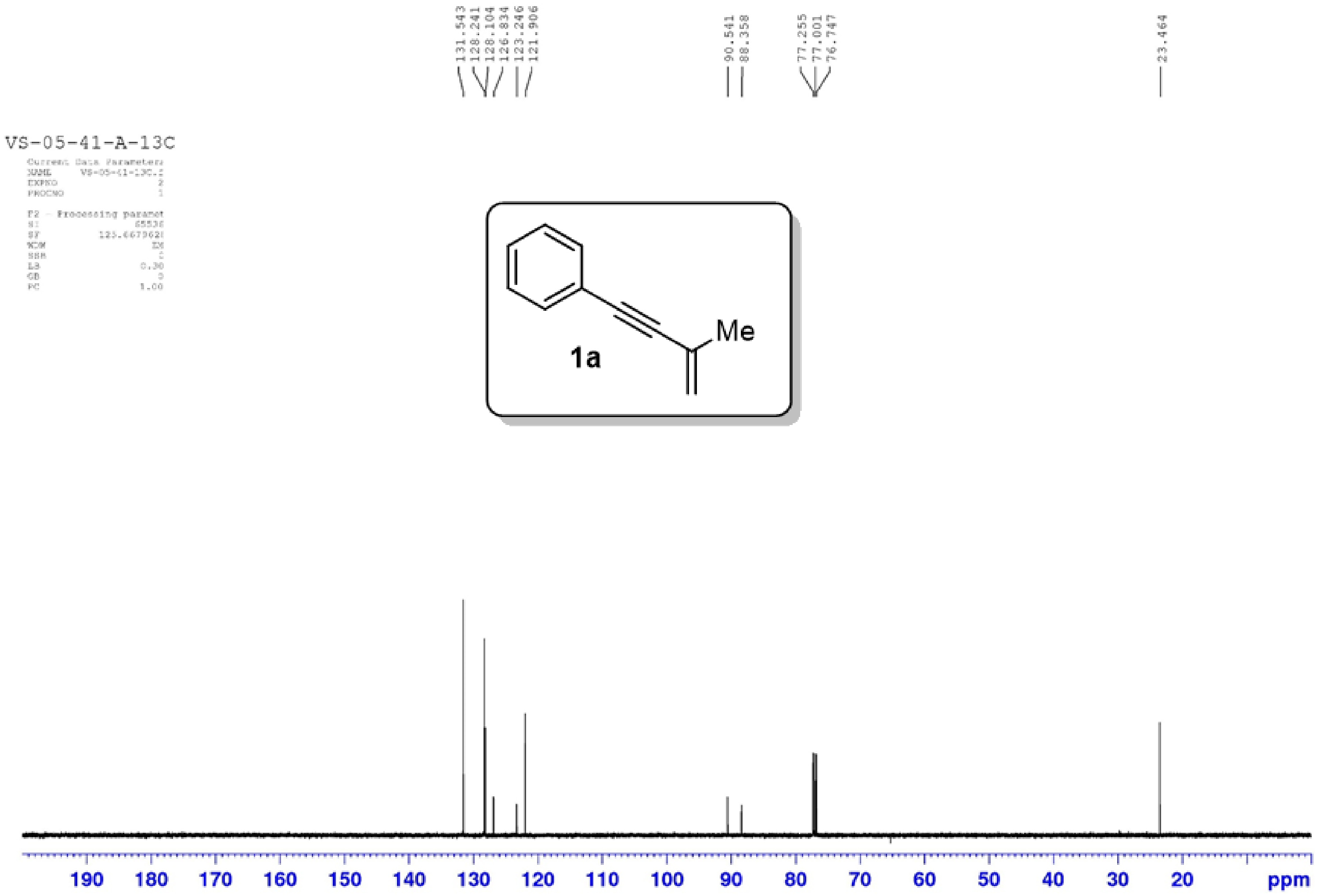

^{1}H-NMR (500 MHz, $CDCl_3$)

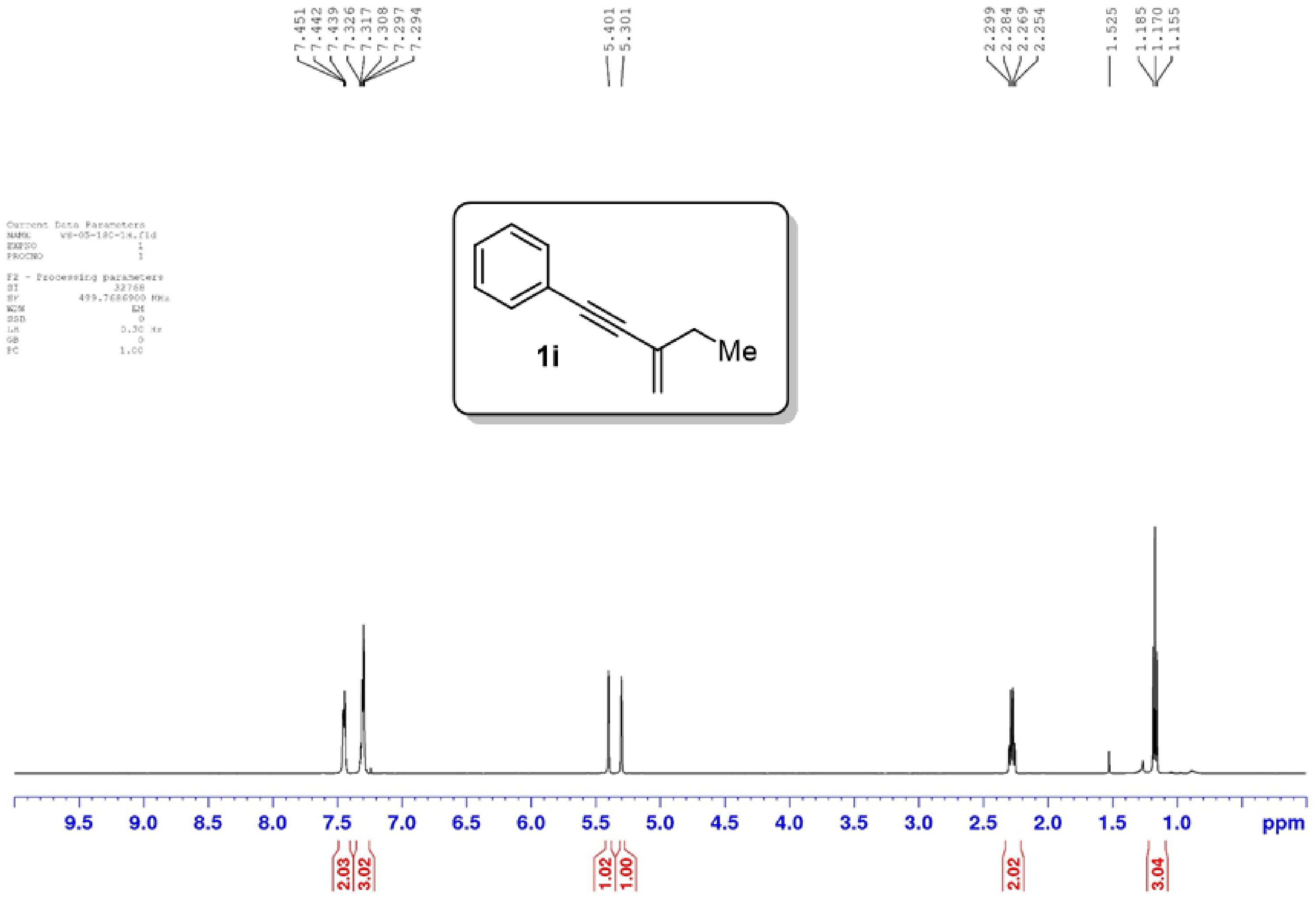

$^{13}C\{1H\}$ and DEPT NMR (125 MHz, $CDCl_3$)

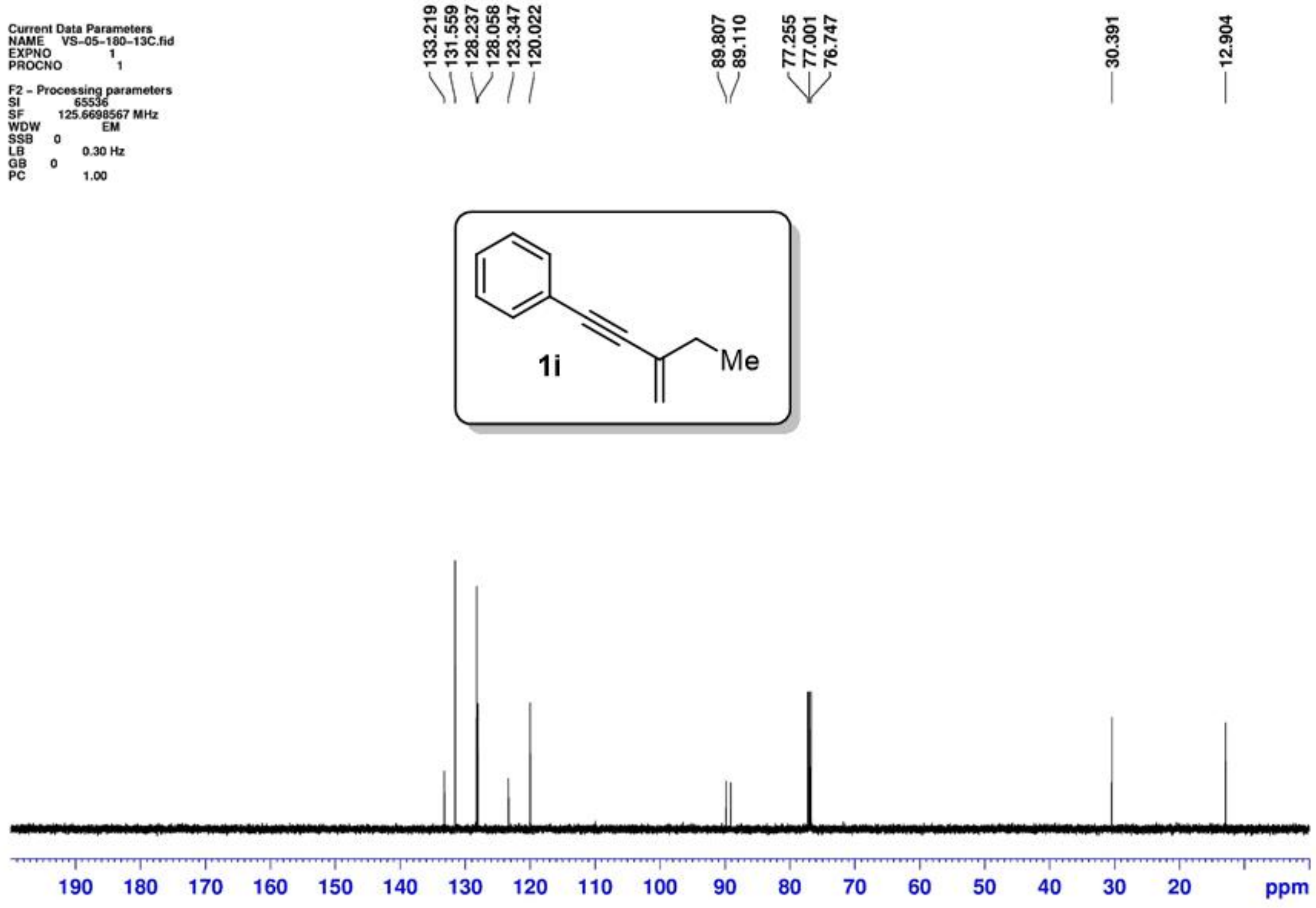

^{1}H-NMR (500 MHz, $CDCl_3$)

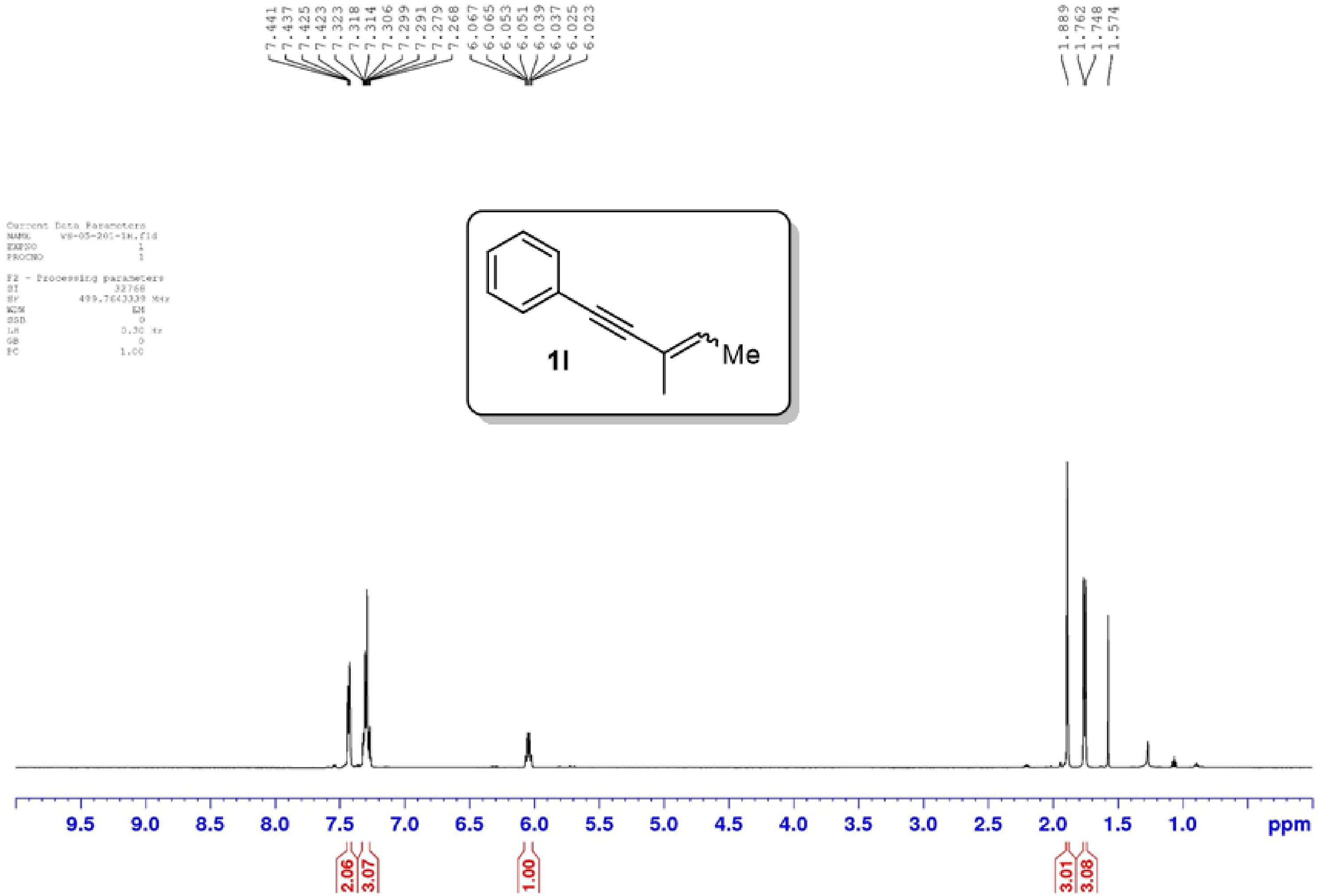

$^{13}C\{1H\}$ **and DEPT NMR (125 MHz, $CDCl_3$)**

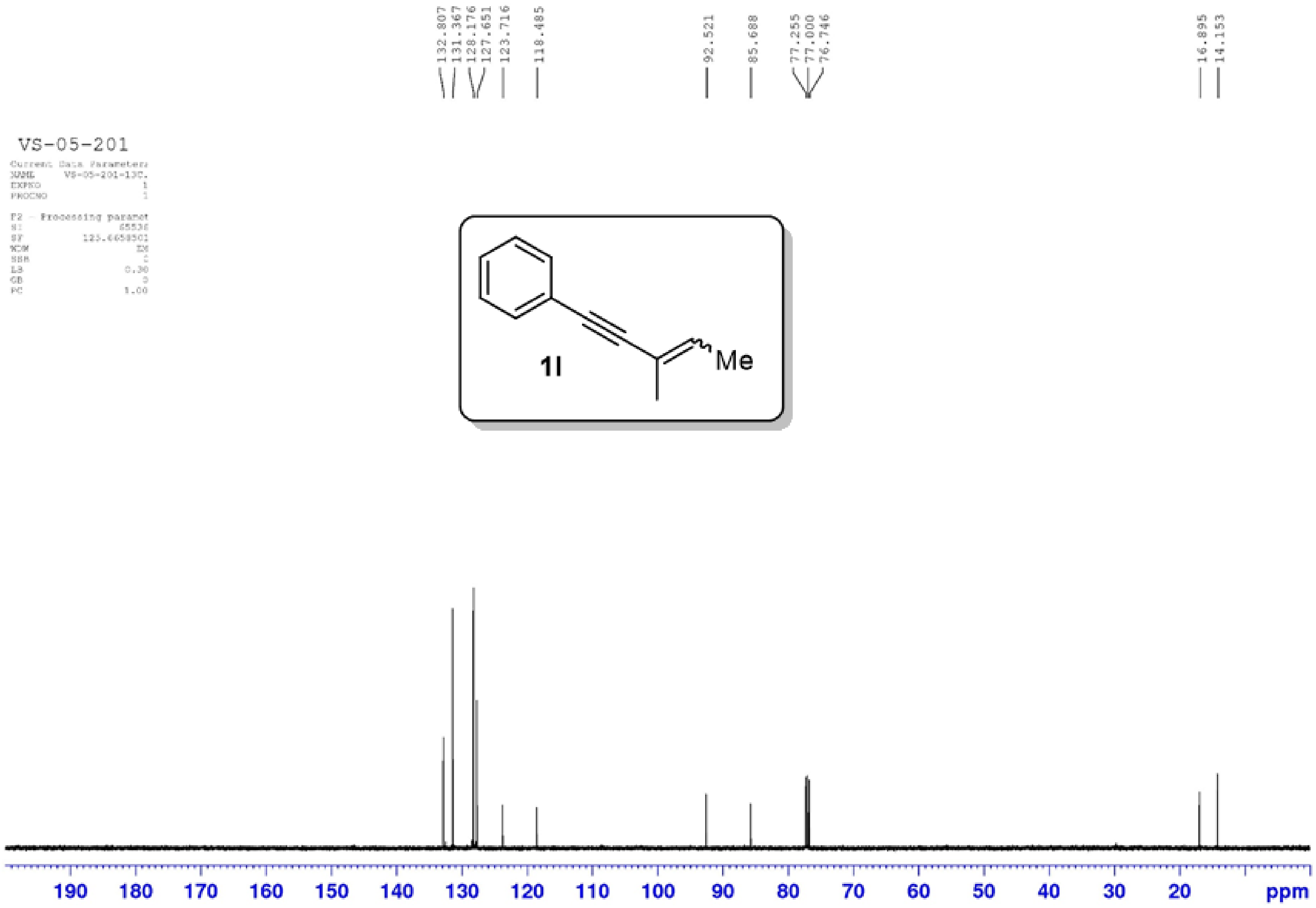

^{1}H-NMR (500 MHz, $CDCl_3$)

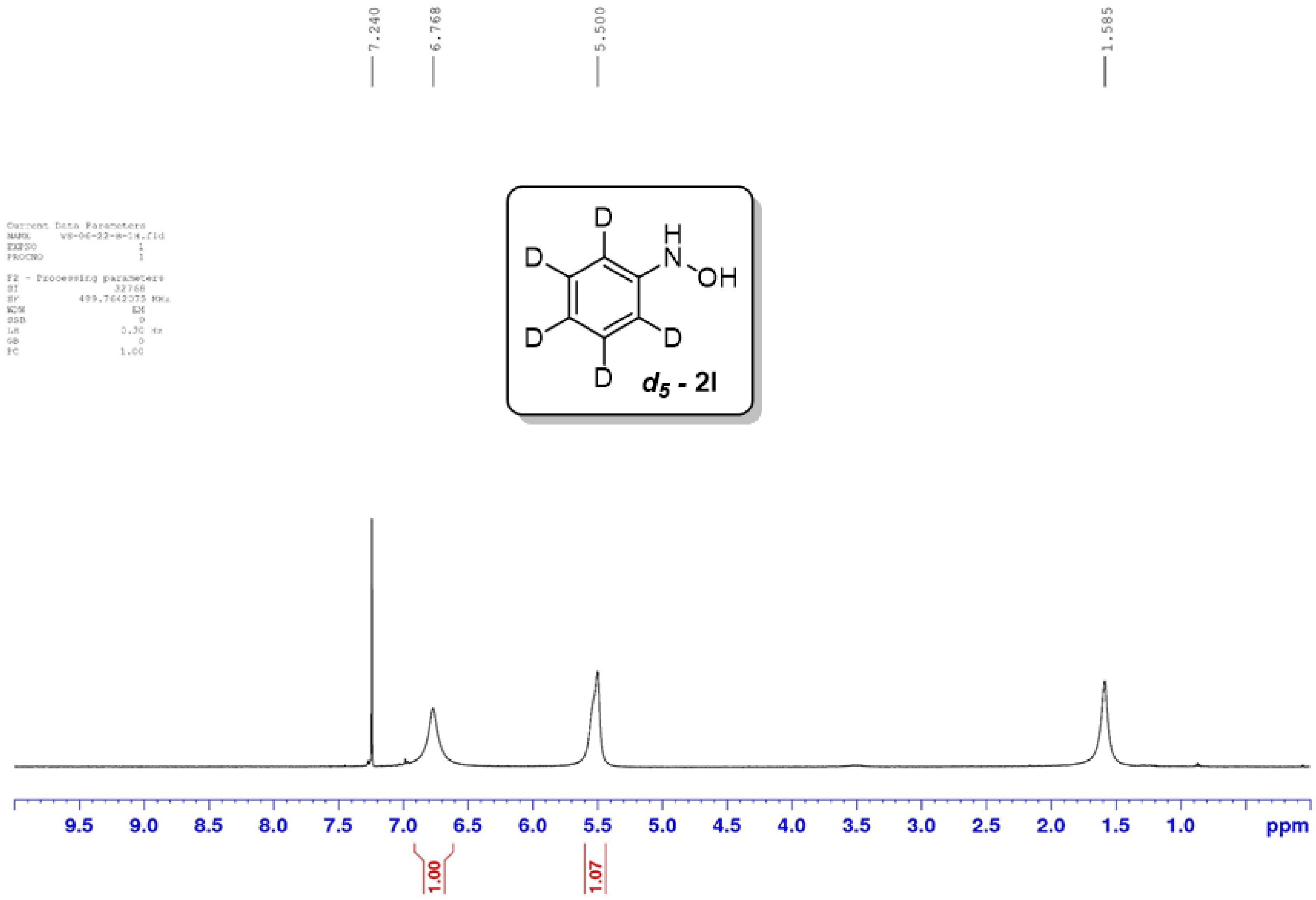

^{13}C{1H} and DEPT NMR (125 MHz, CDCl$_3$)

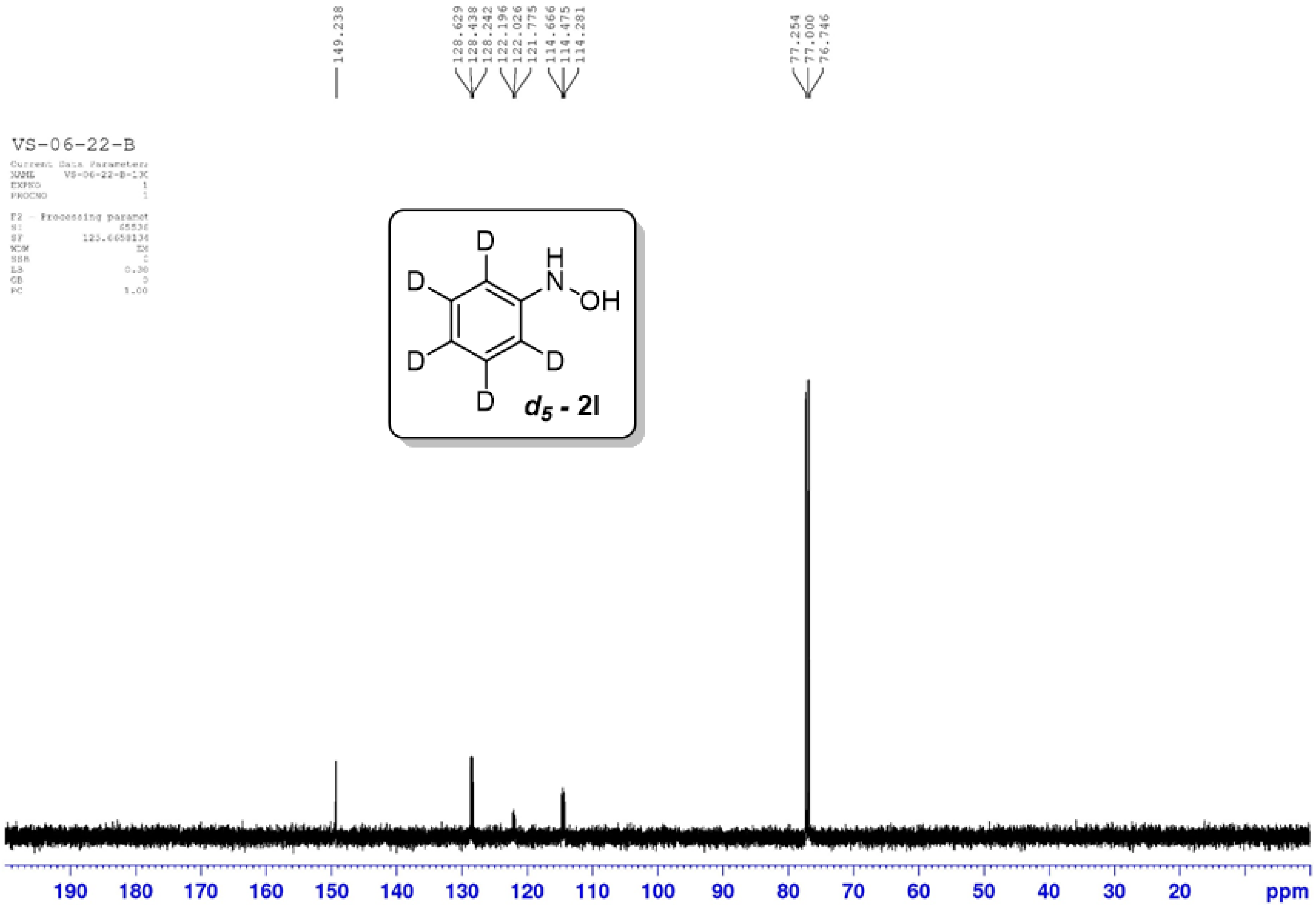

^{1}H-NMR (700 MHz, $CDCl_3$)

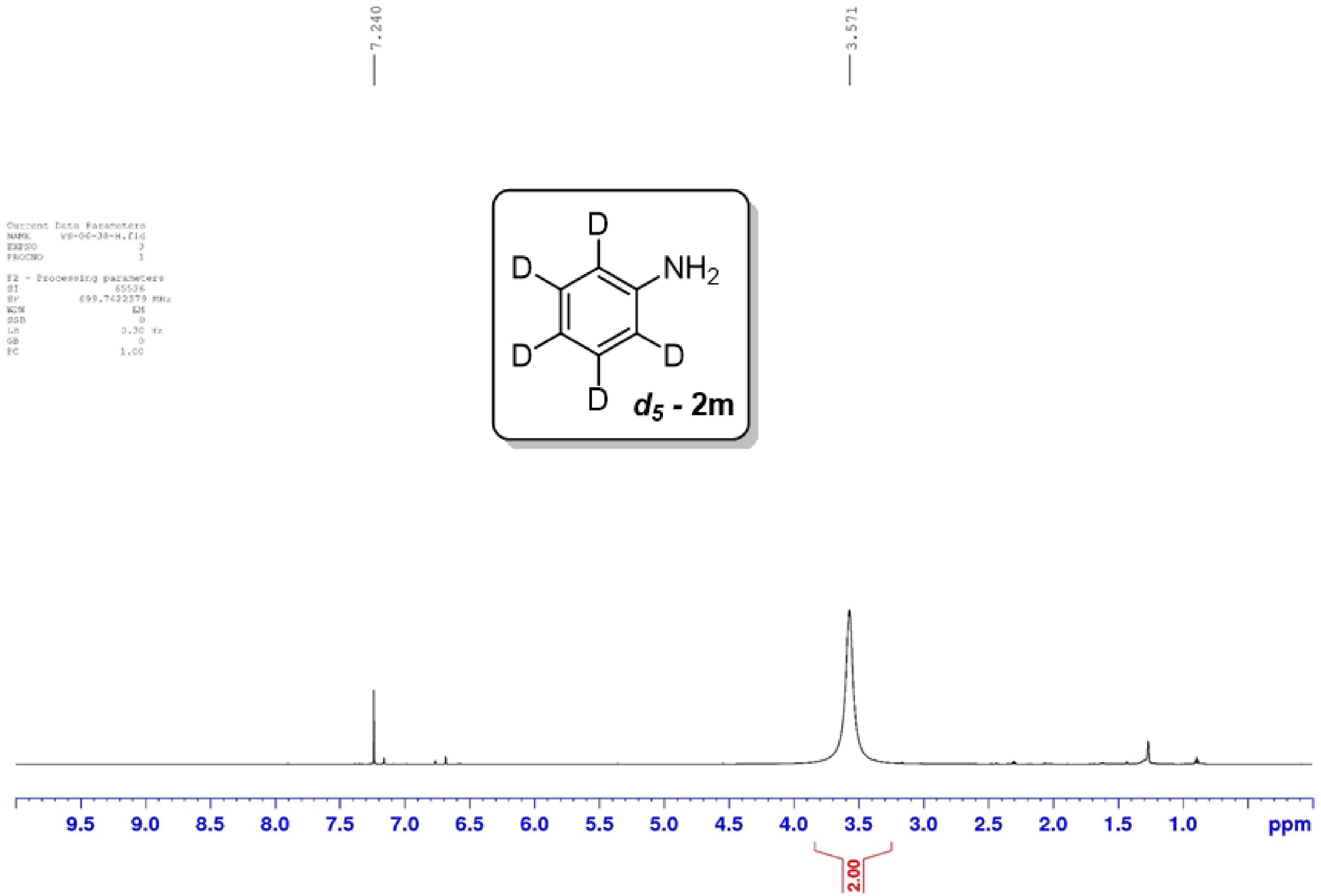

$^{13}C\{1H\}$ and DEPT NMR (175 MHz, $CDCl_3$)

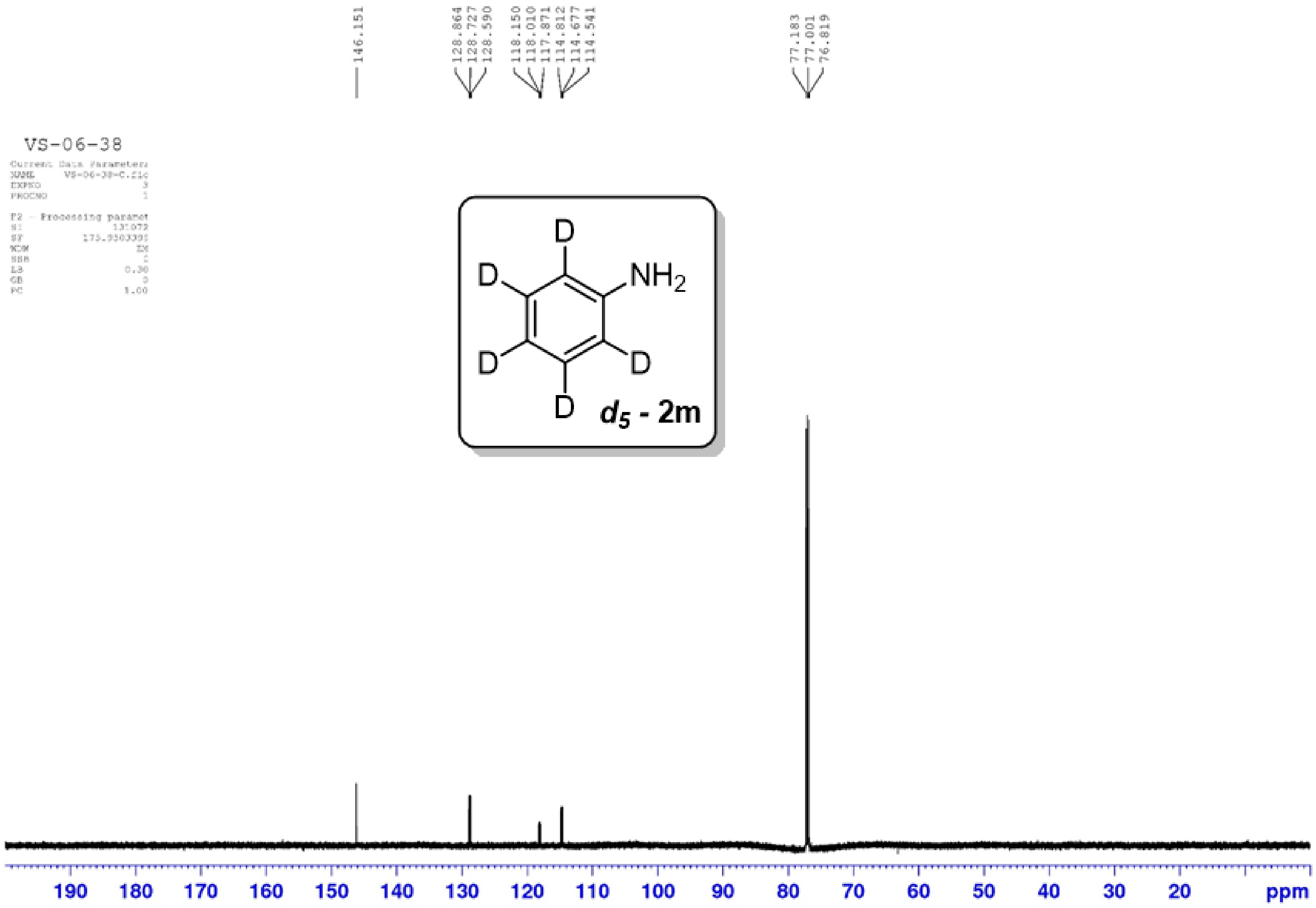

^{1}H-NMR (700 MHz, $CDCl_3$)

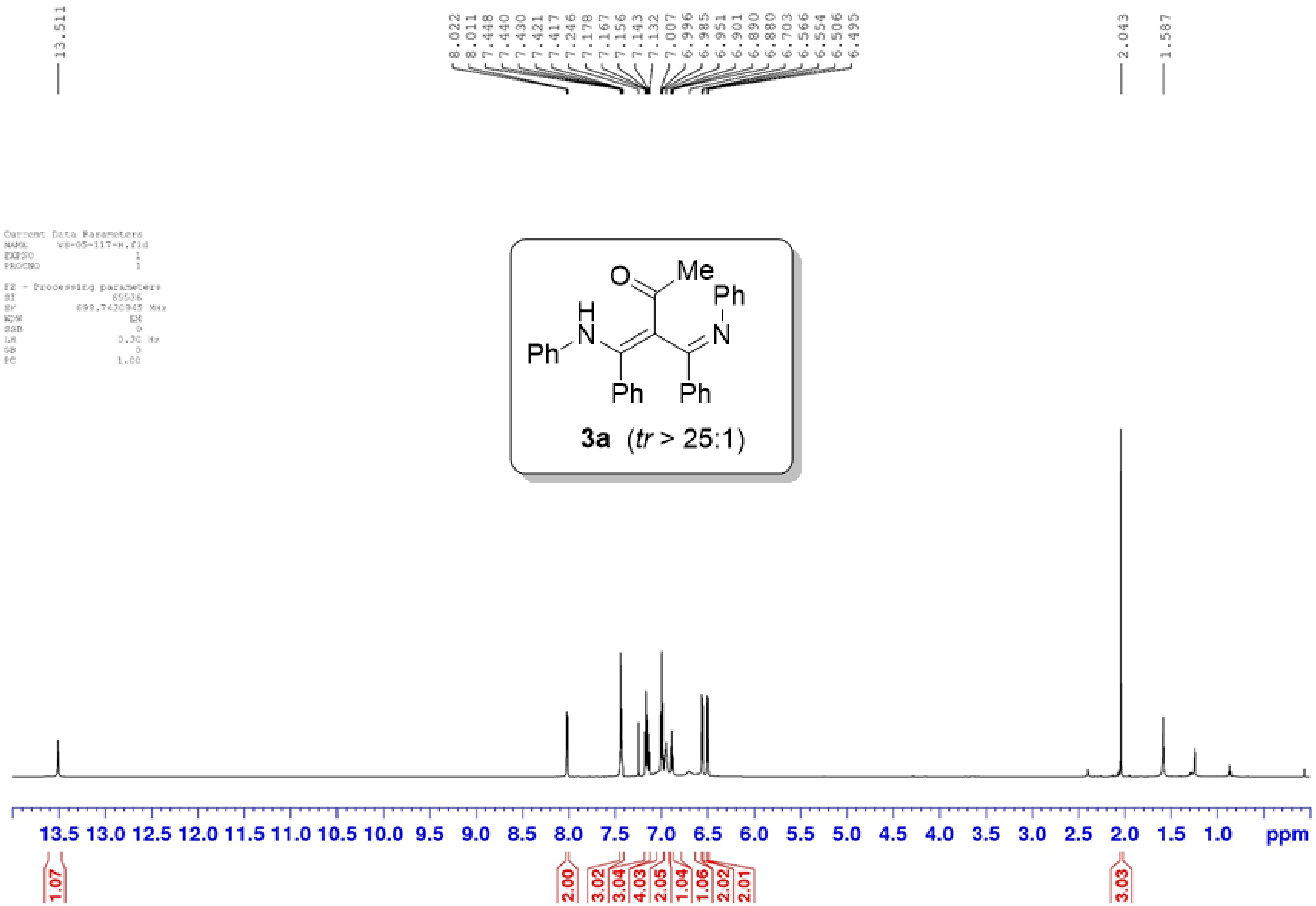

$^{13}C\{1H\}$ and DEPT NMR (175 MHz, $CDCl_3$)

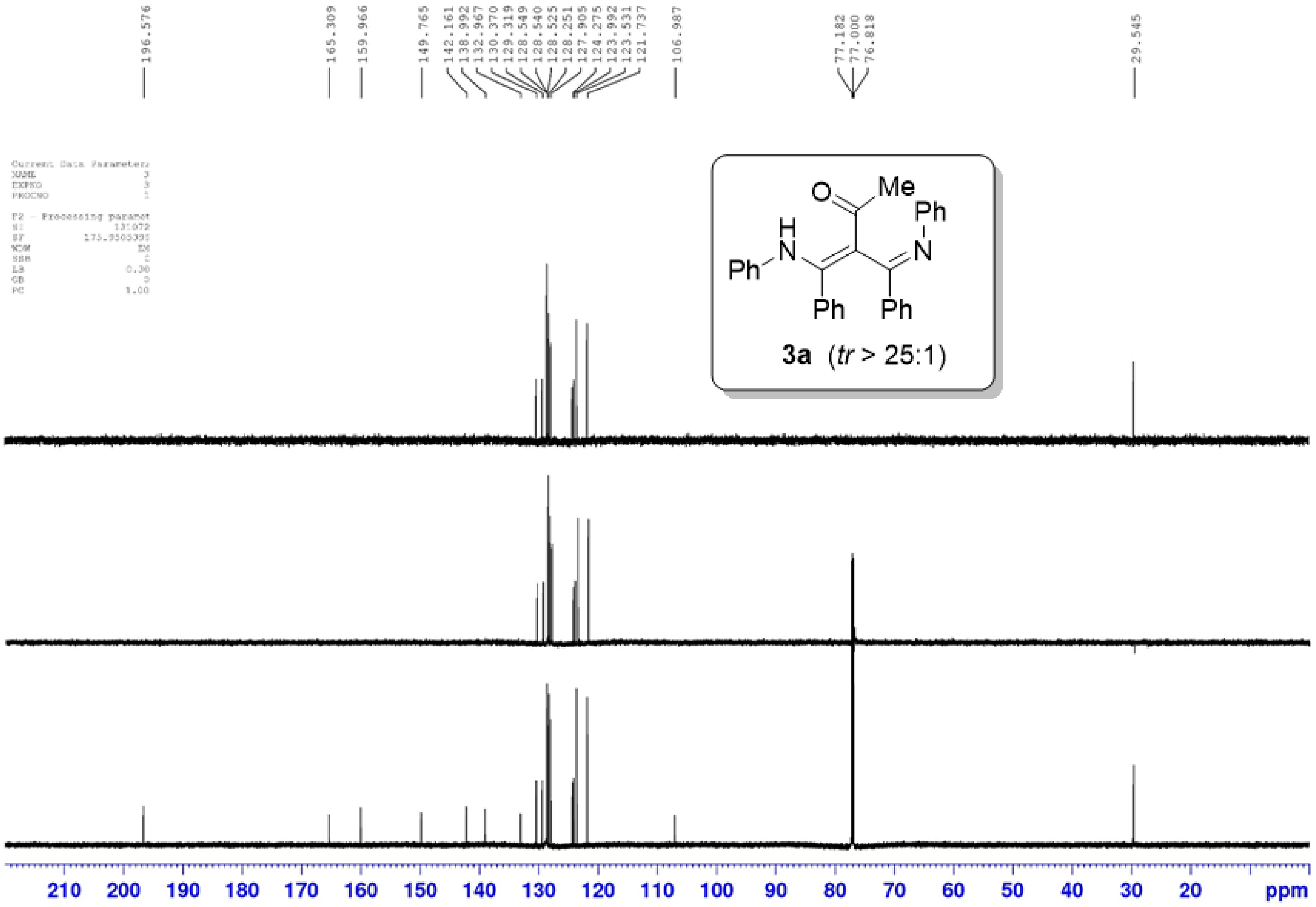

^{1}H-NMR 700 MHz, $CDCl_3$)

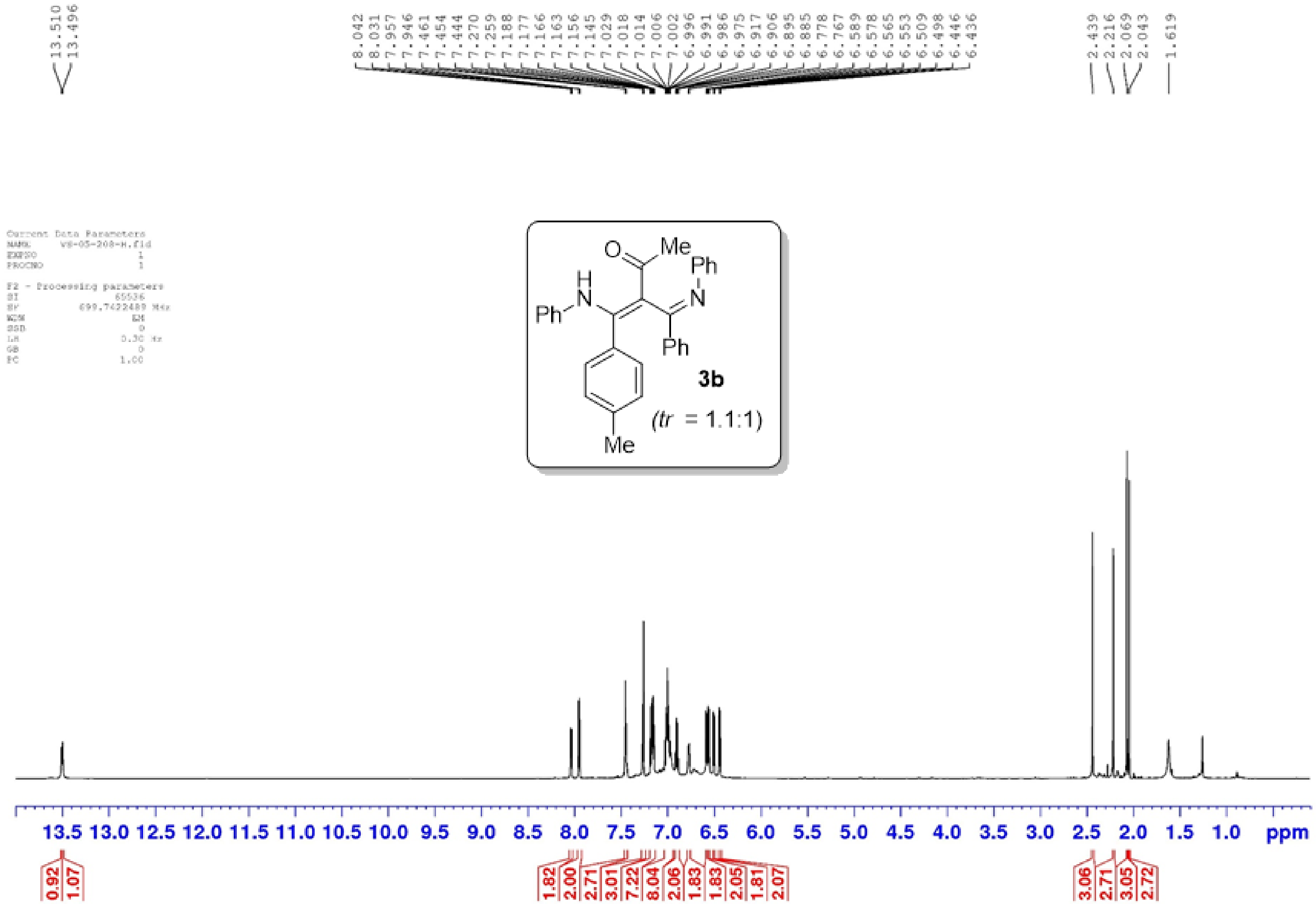

^{13}C{1H} and DEPT NMR (175 MHz, $CDCl_3$)

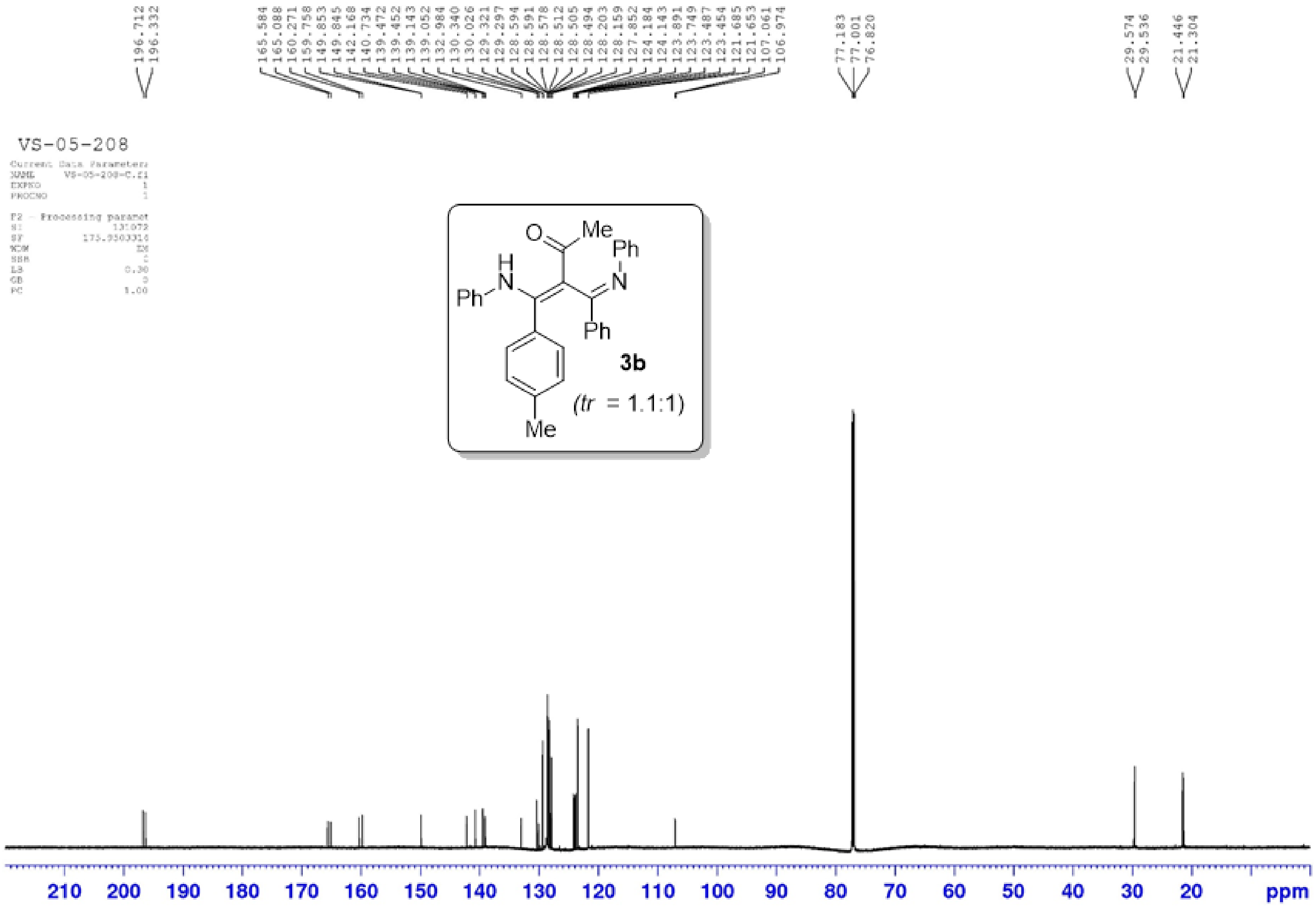

^{1}H-NMR (400 MHz, $CDCl_3$)

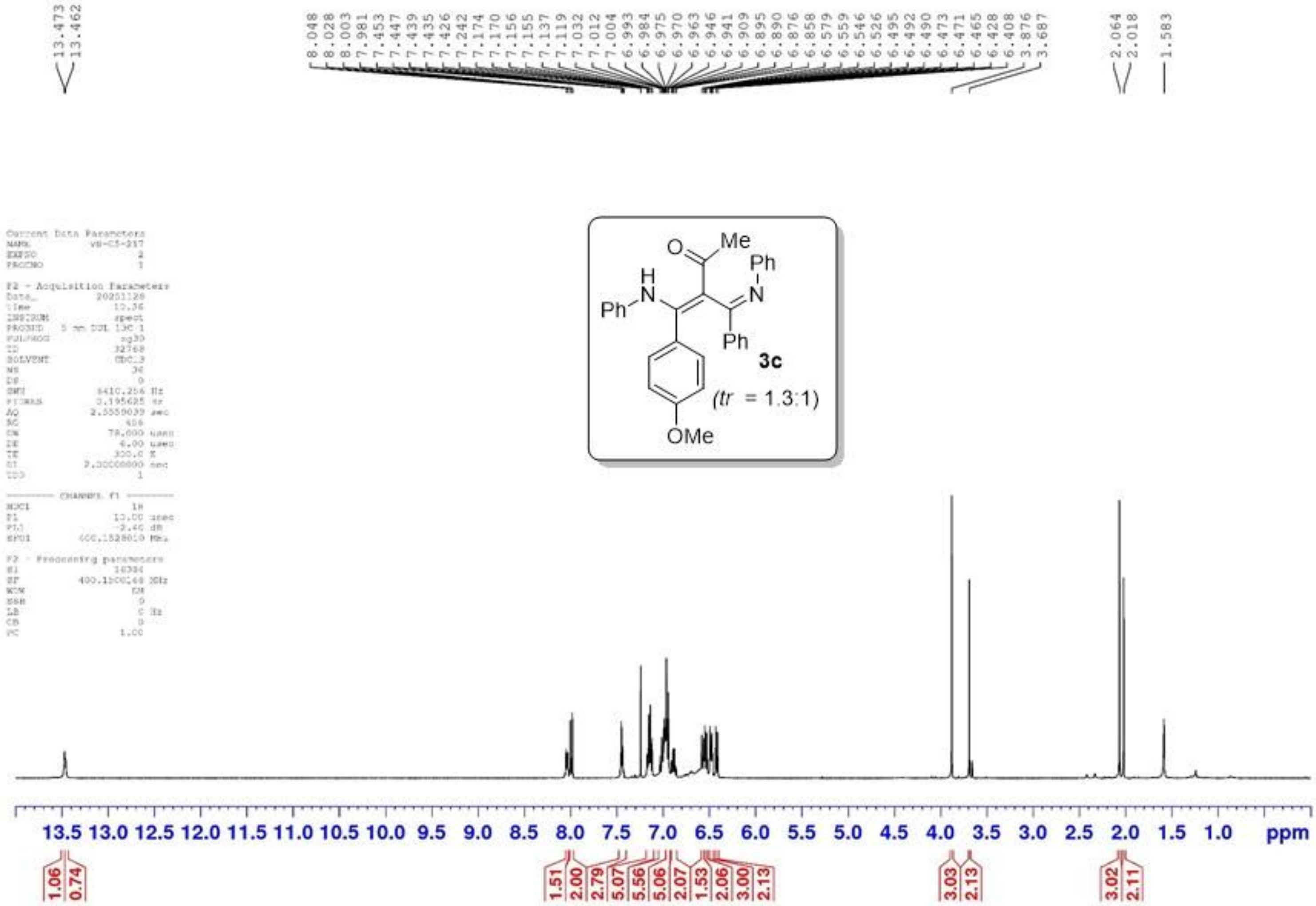

$^{13}C\{^1H\}$ and DEPT NMR (175 MHz, $CDCl_3$)

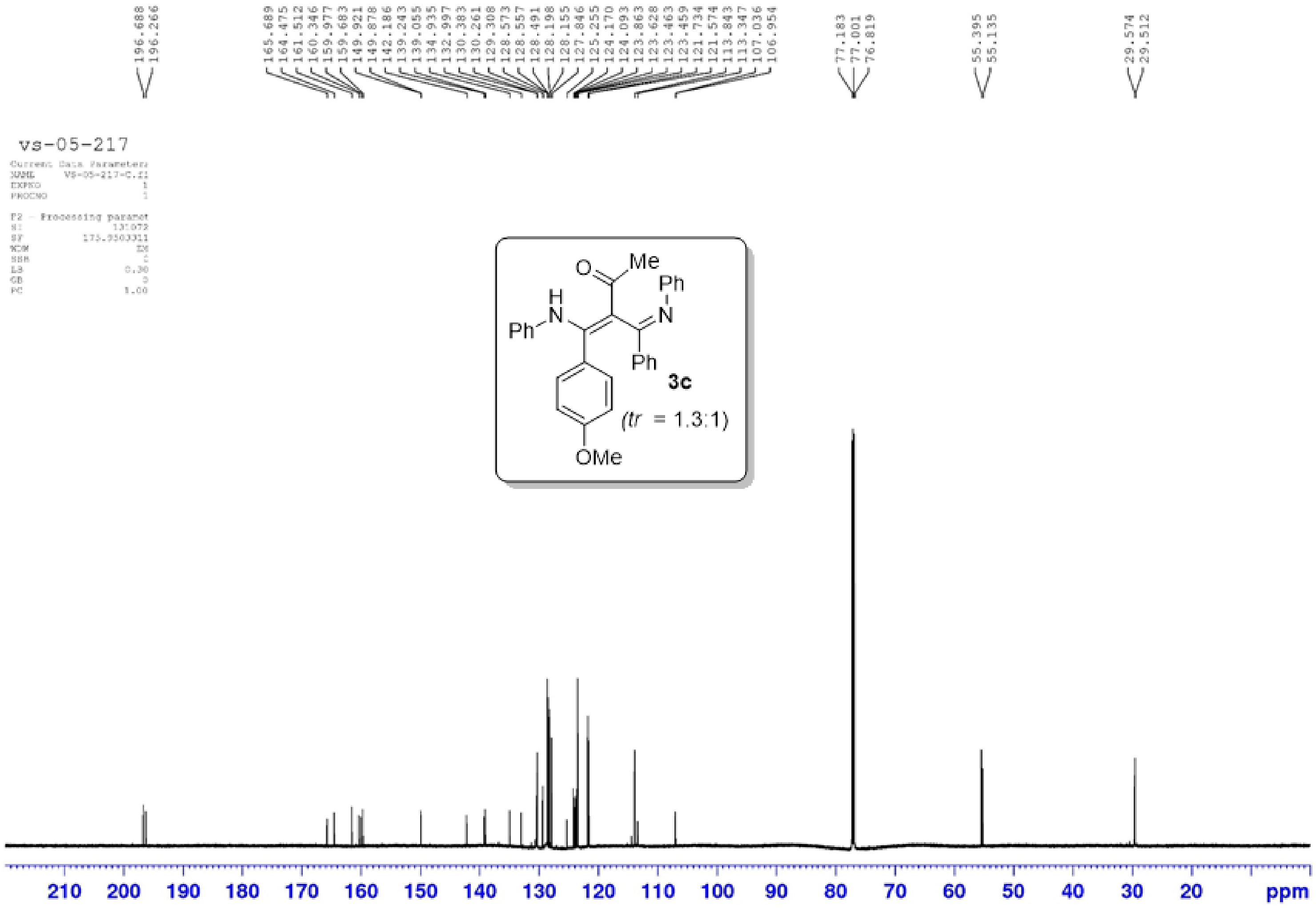

^{1}H-NMR (700 MHz, $CDCl_3$)

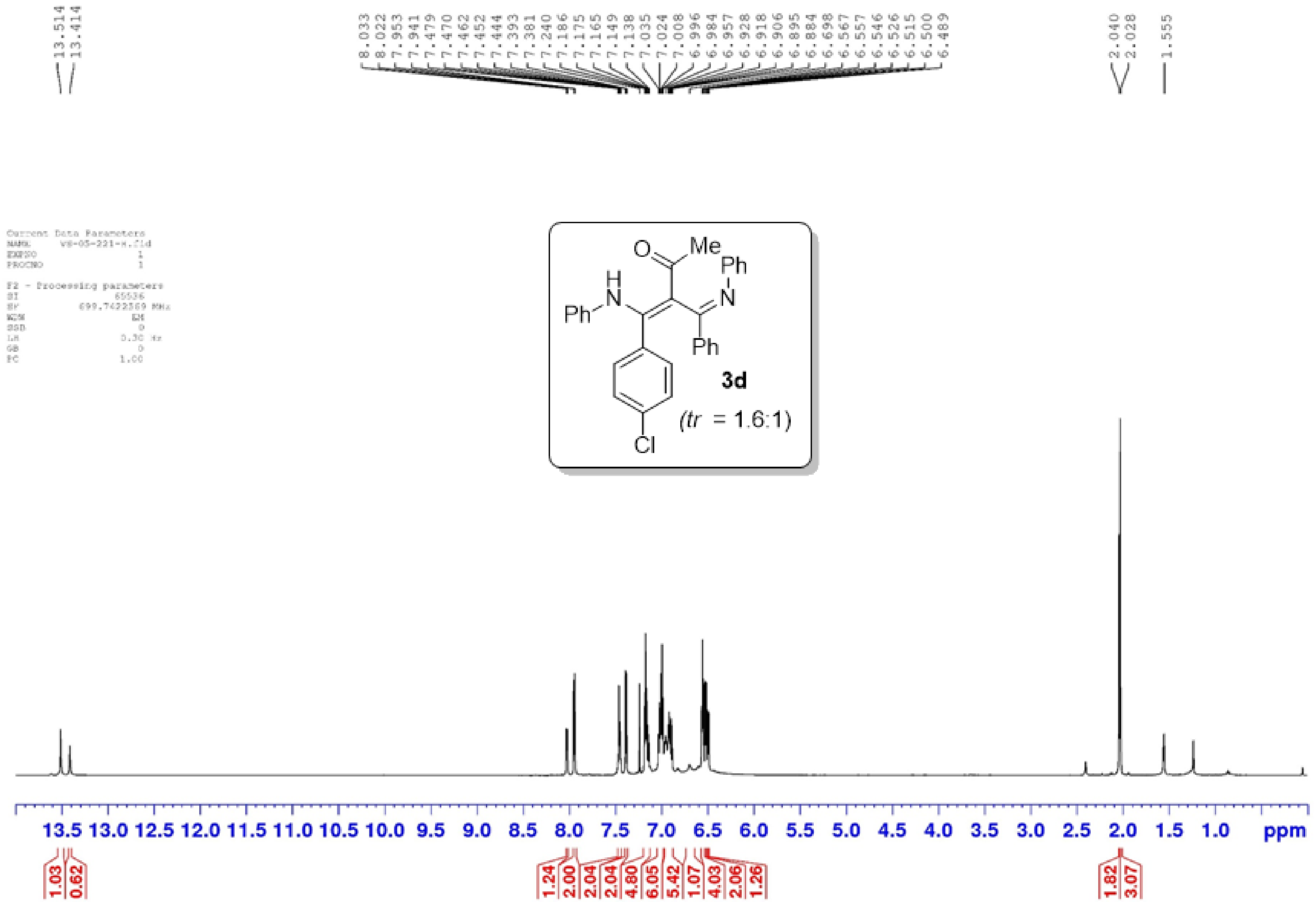

$^{13}C\{1H\}$ and DEPT NMR (175 MHz, $CDCl_3$)

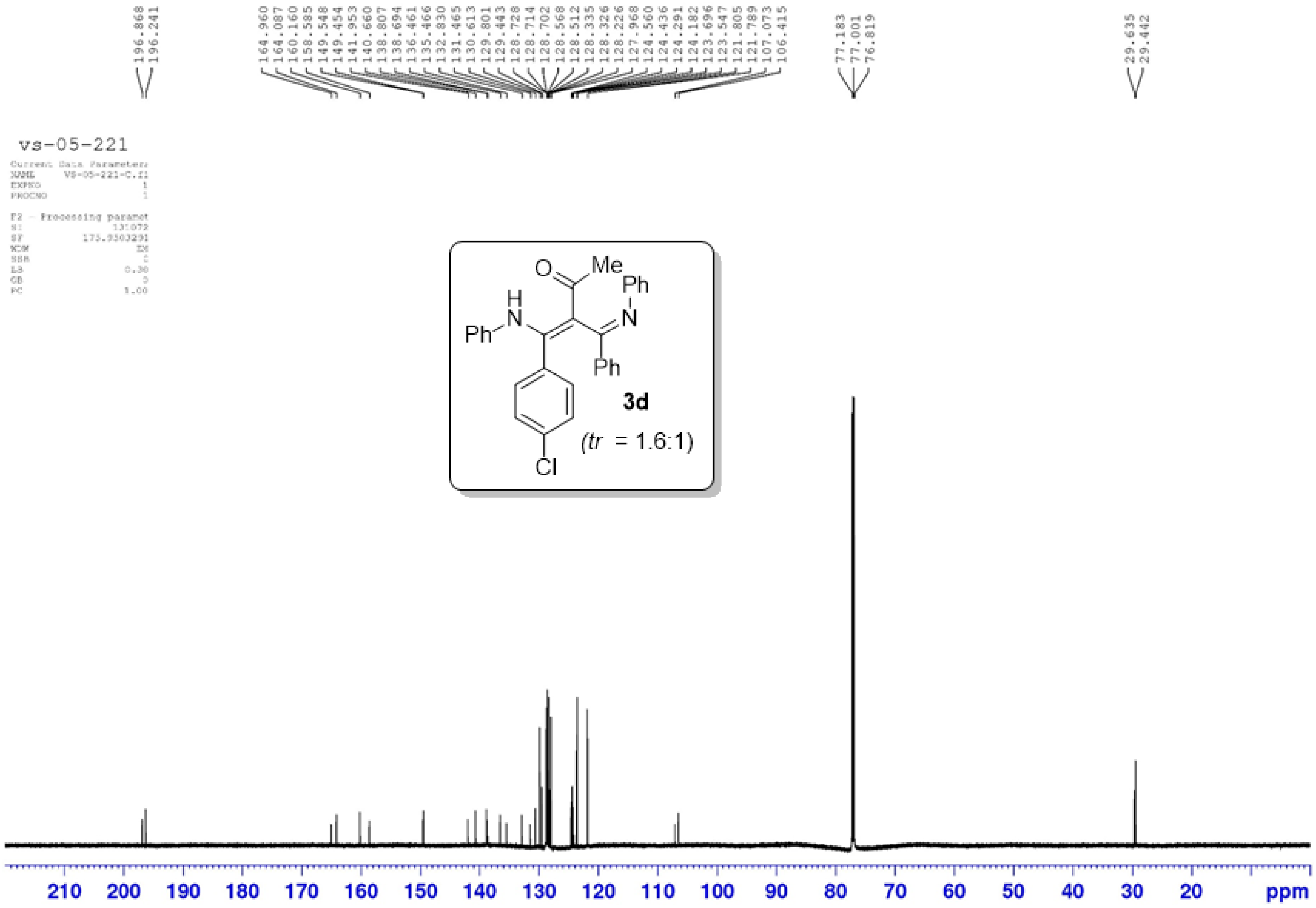

^{1}H-NMR (500 MHz, $CDCl_3$)

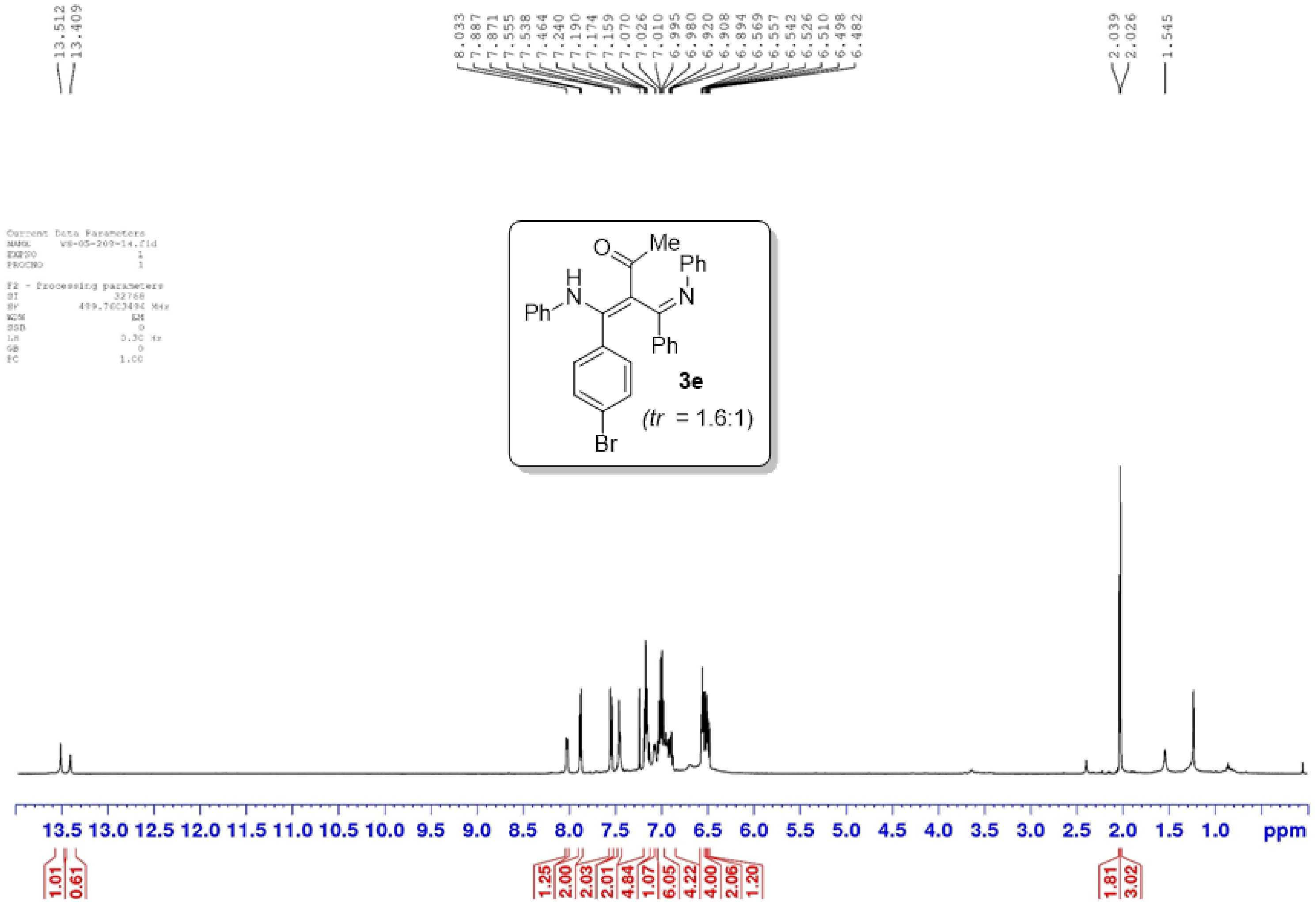

^{13}C{1H} and DEPT NMR (125 MHz, $CDCl_3$)

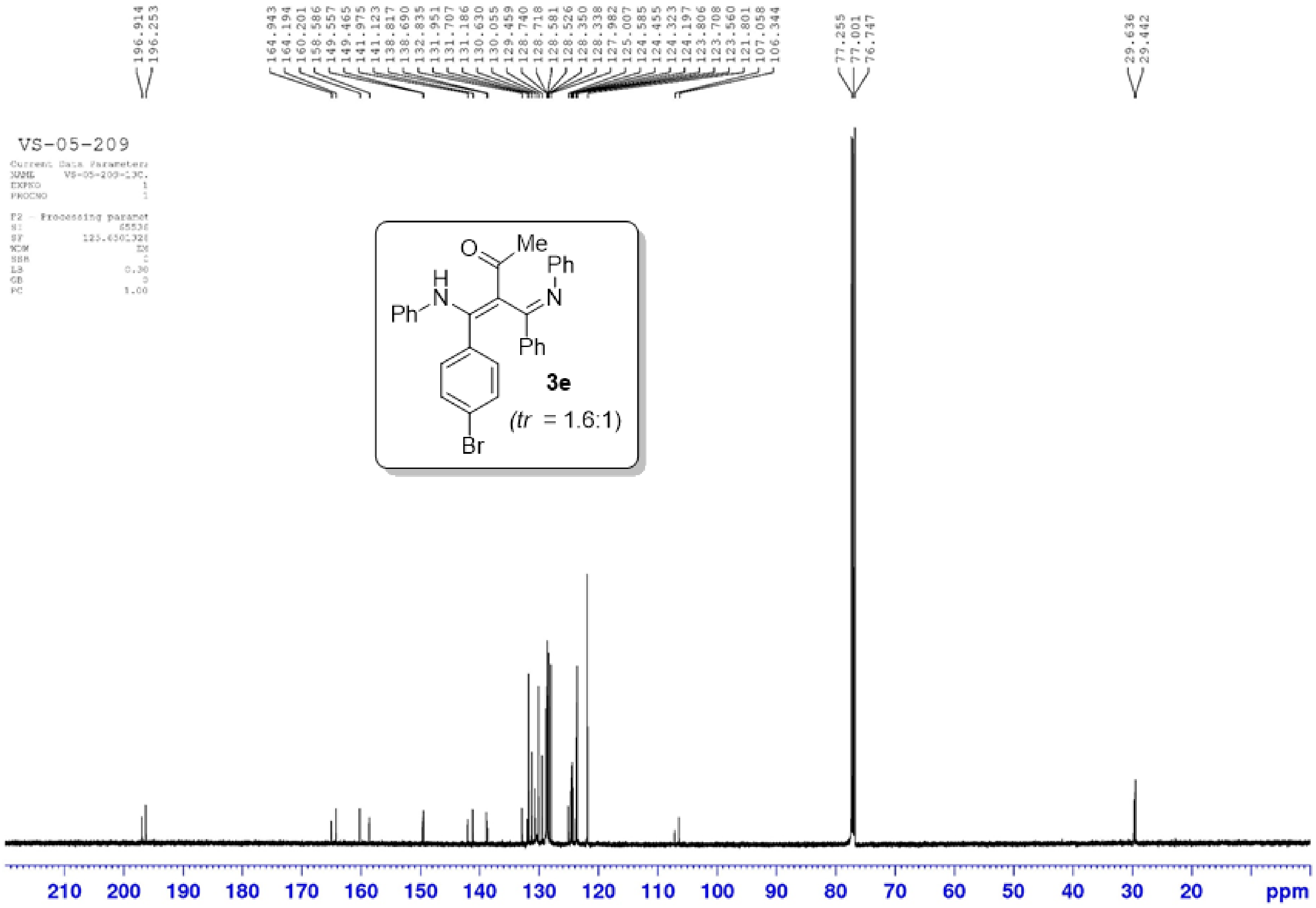

^{1}H-NMR (400 MHz, $CDCl_3$)

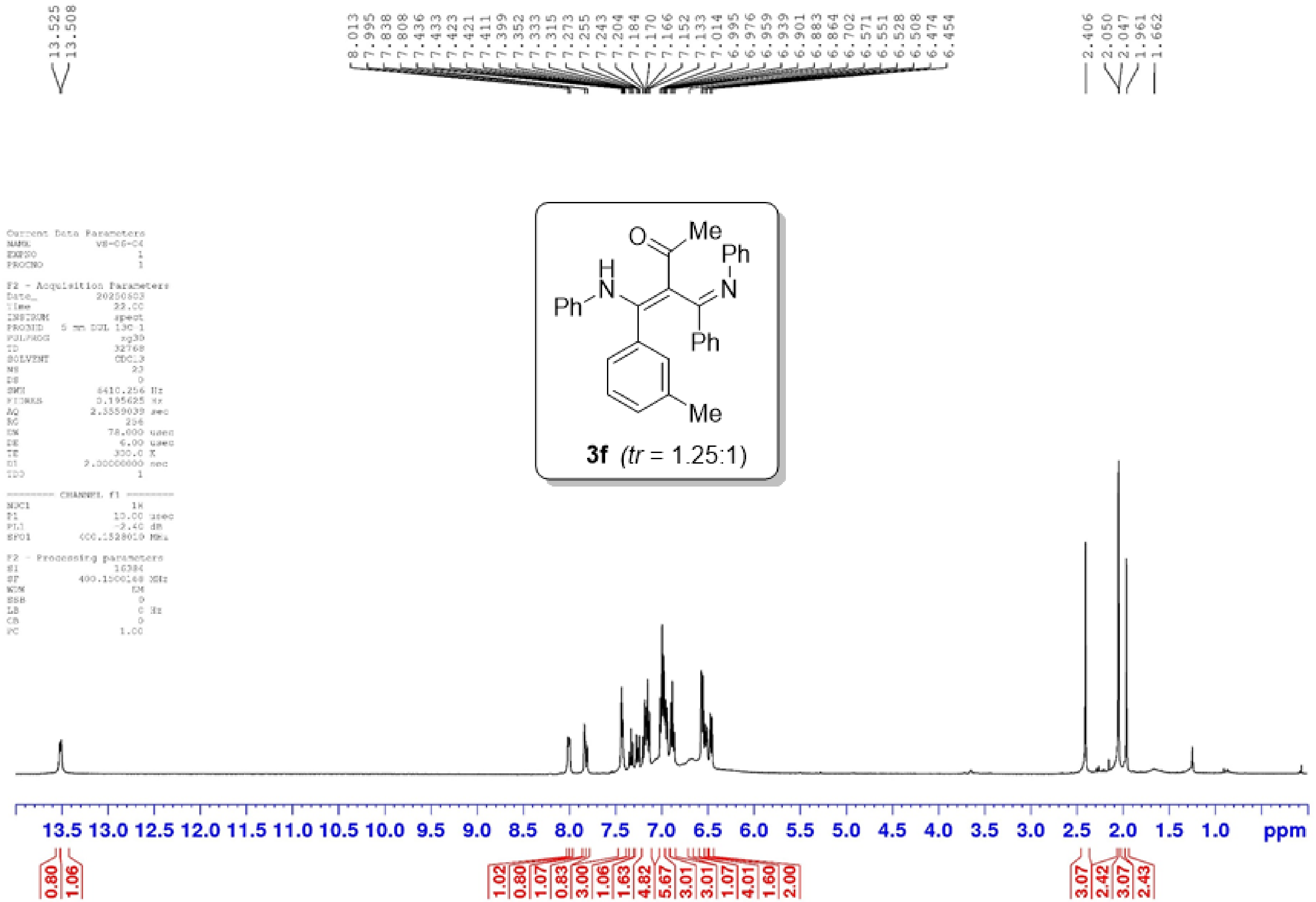

$^{13}C\{1H\}$ **and DEPT NMR (125 MHz, $CDCl_3$)**

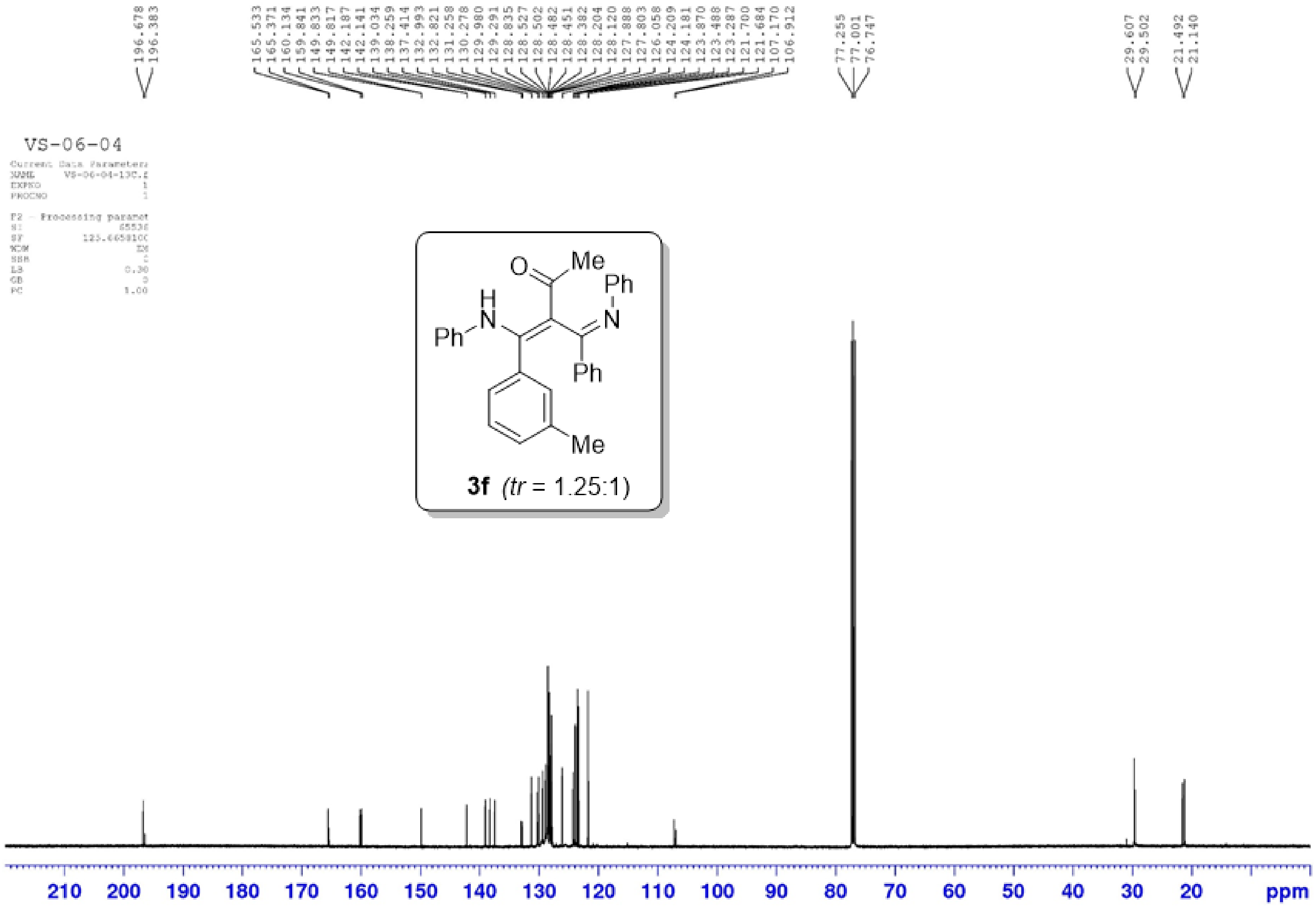

^{1}H-NMR (500 MHz, $CDCl_3$)

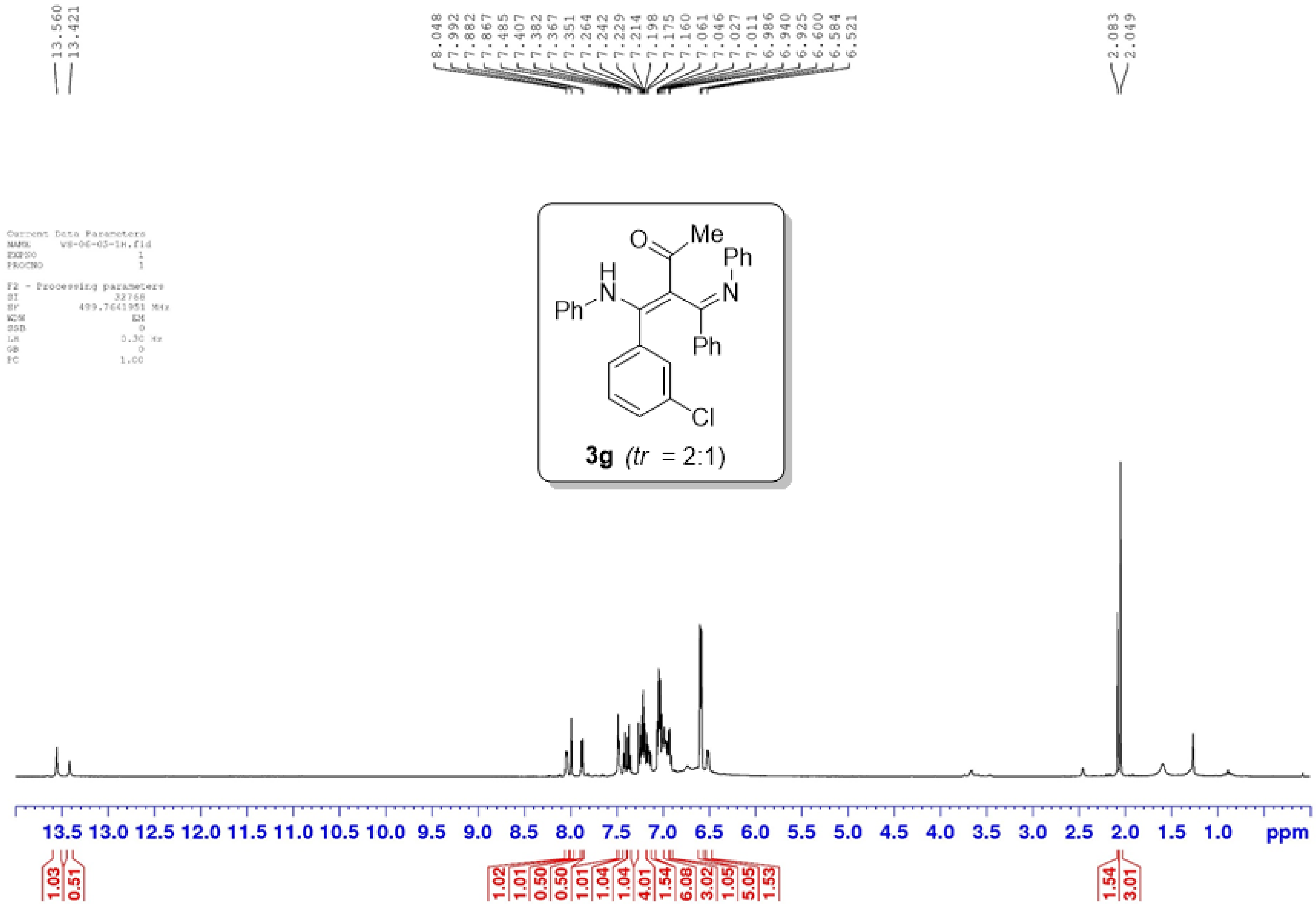

$^{13}C\{1H\}$ and DEPT NMR (100 MHz, $CDCl_3$)

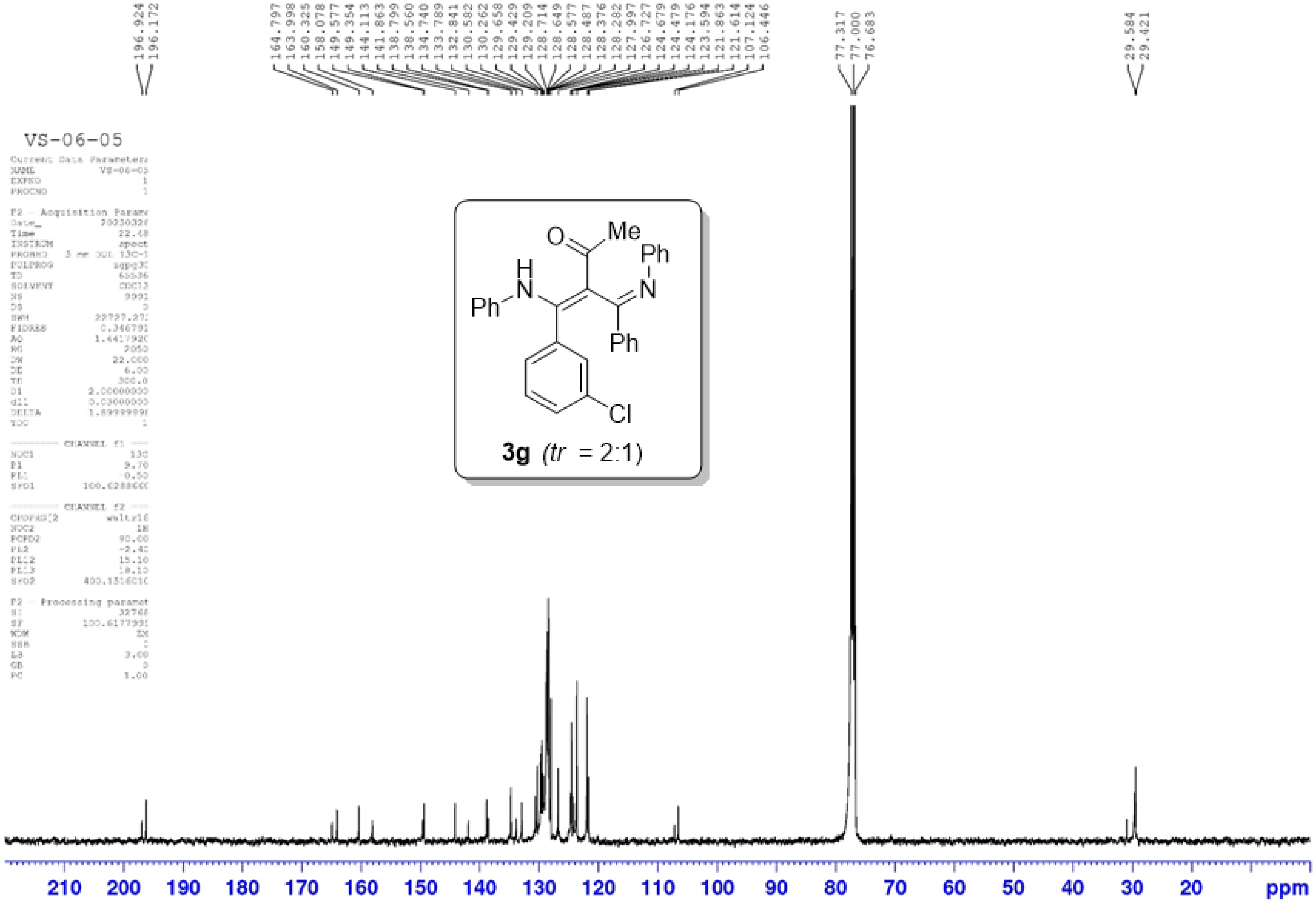

^{1}**H-NMR (700 MHz, CDCl$_3$)**

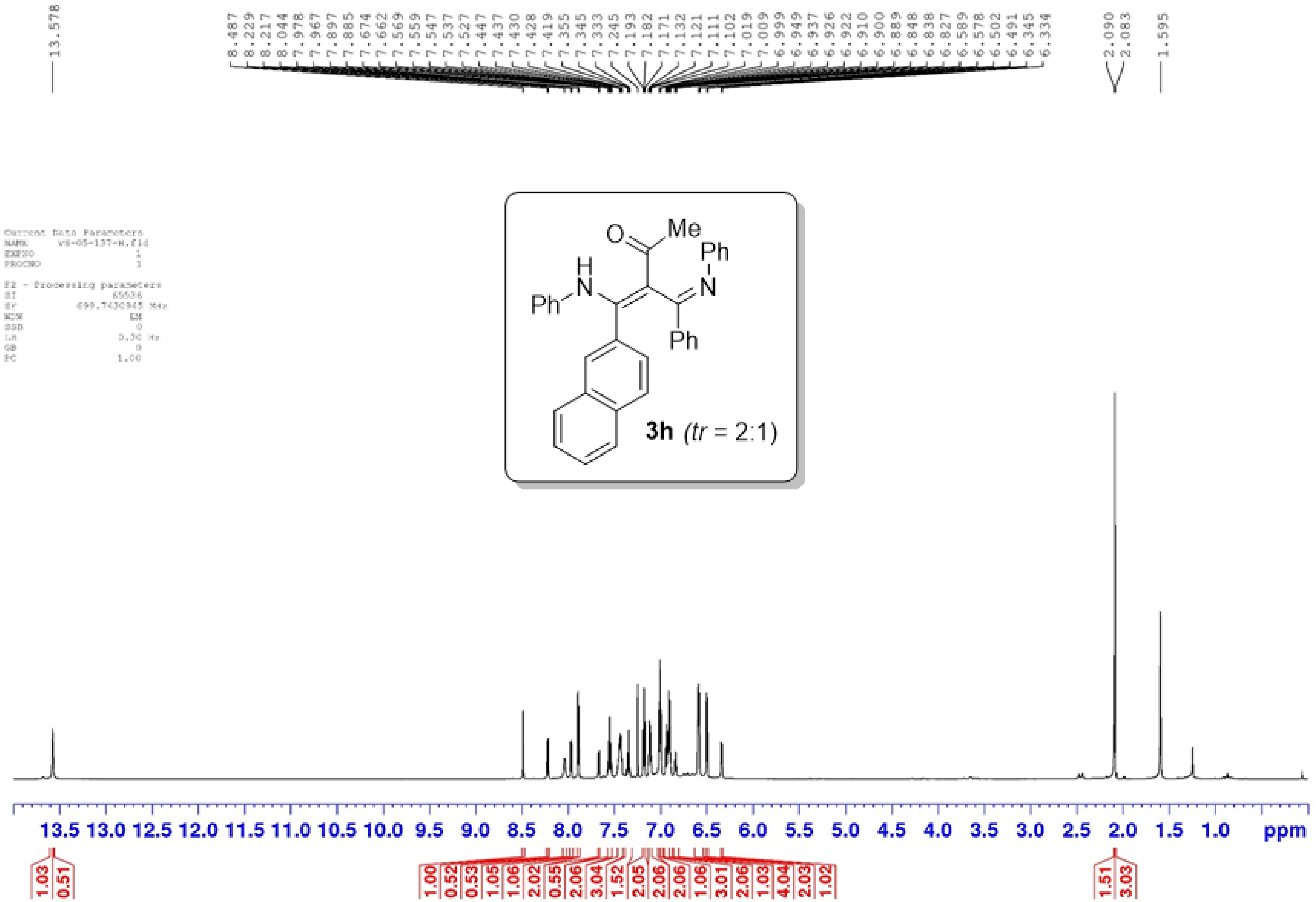

$^{13}C\{1H\}$ **and DEPT NMR (175 MHz, $CDCl_3$)**

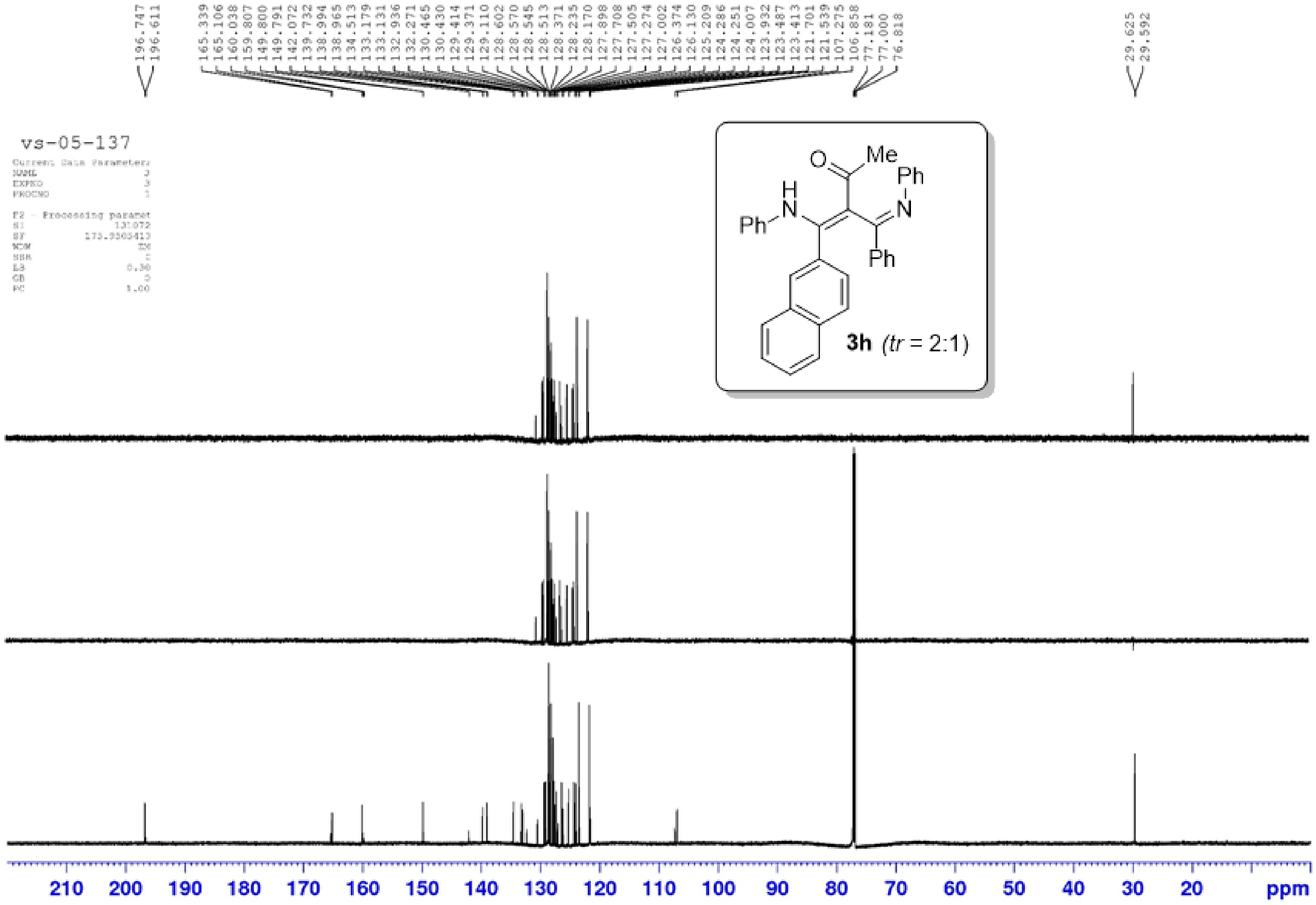

^{1}H-NMR (400 MHz, $CDCl_3$)

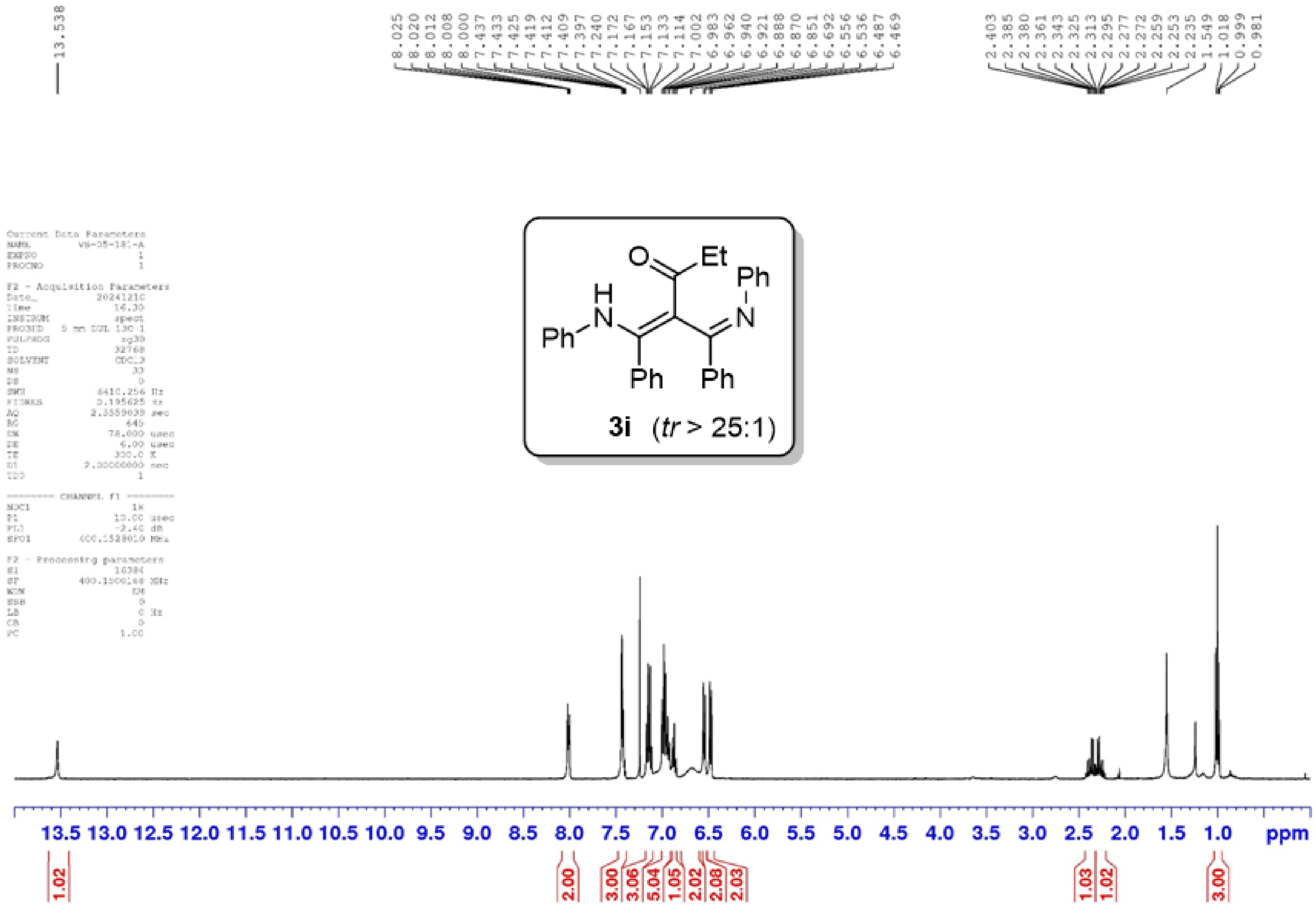

$^{13}C\{1H\}$ and DEPT NMR (175 MHz, $CDCl_3$)

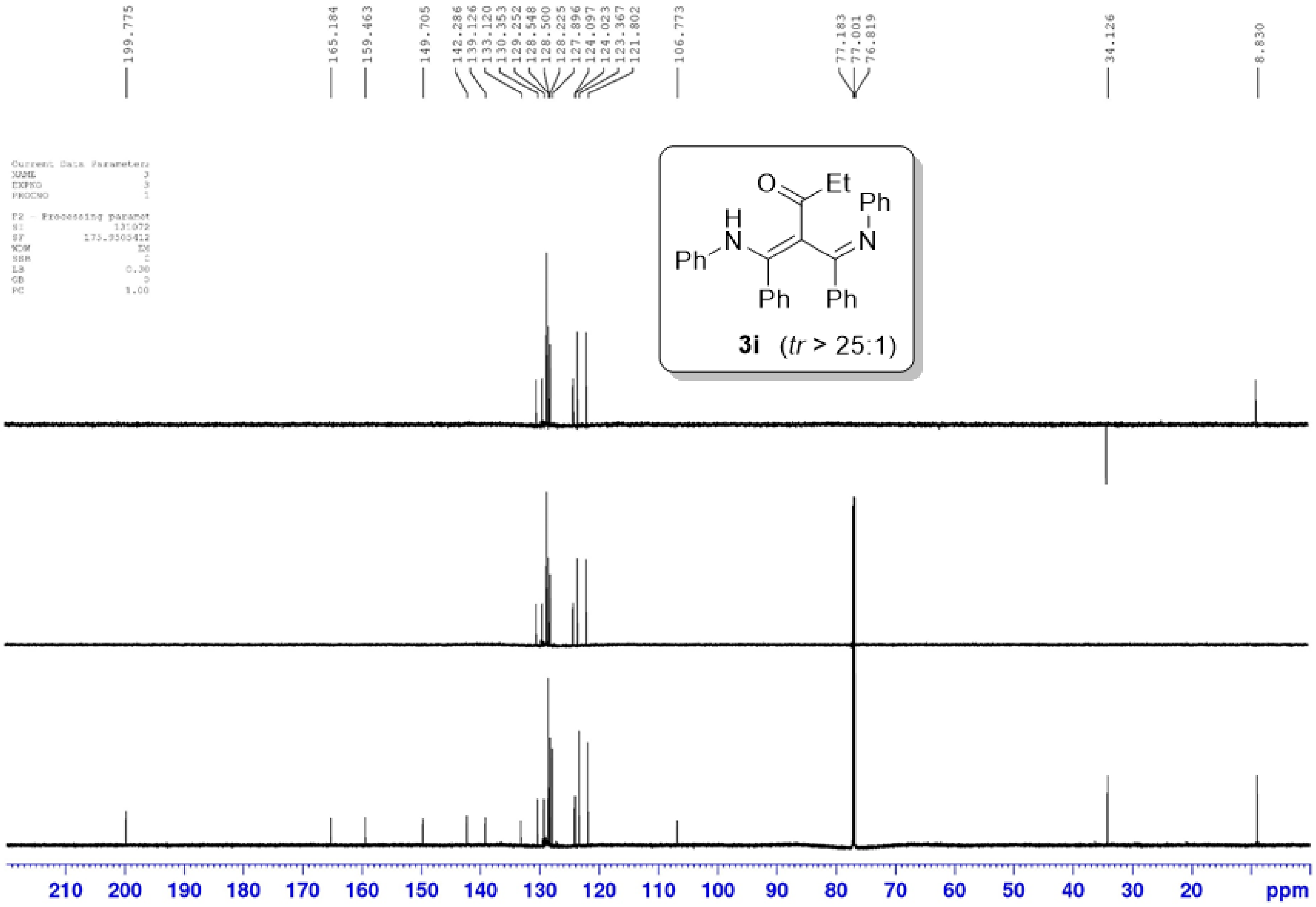

^{1}H-NMR (400 MHz, $CDCl_3$)

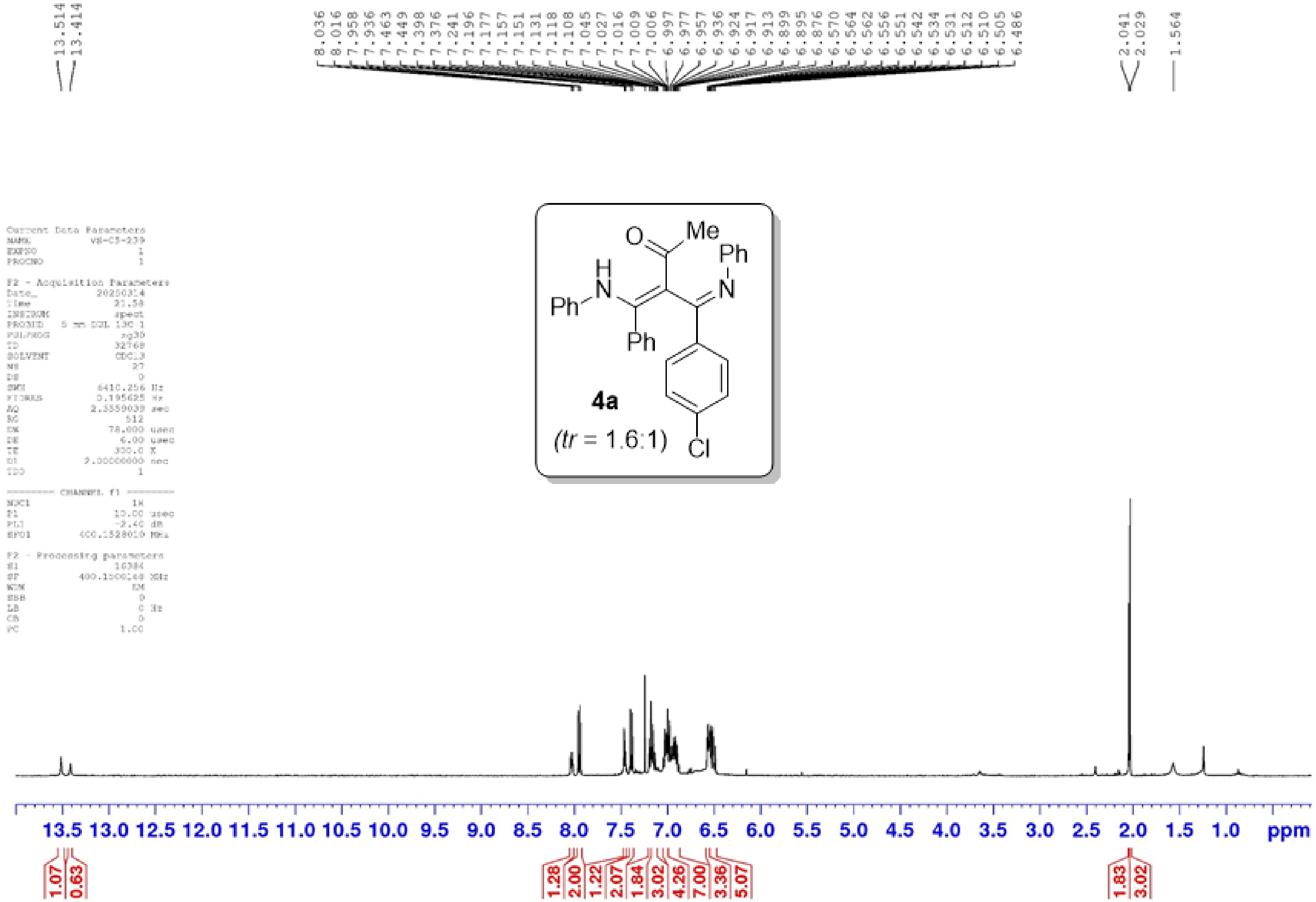

$^{13}C\{1H\}$ and DEPT NMR (100 MHz, $CDCl_3$)

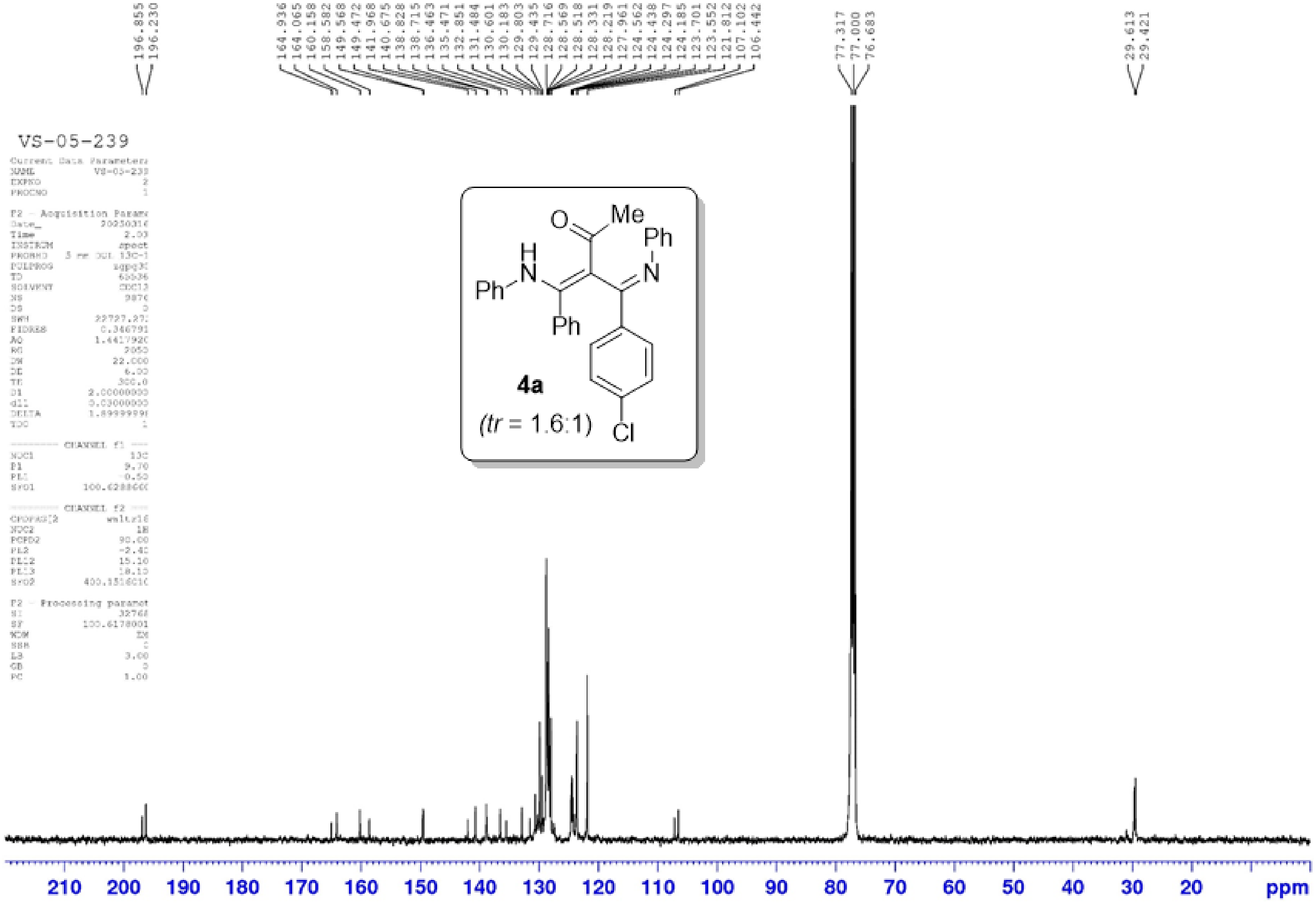

^{1}H-NMR (700 MHz, $CDCl_3$)

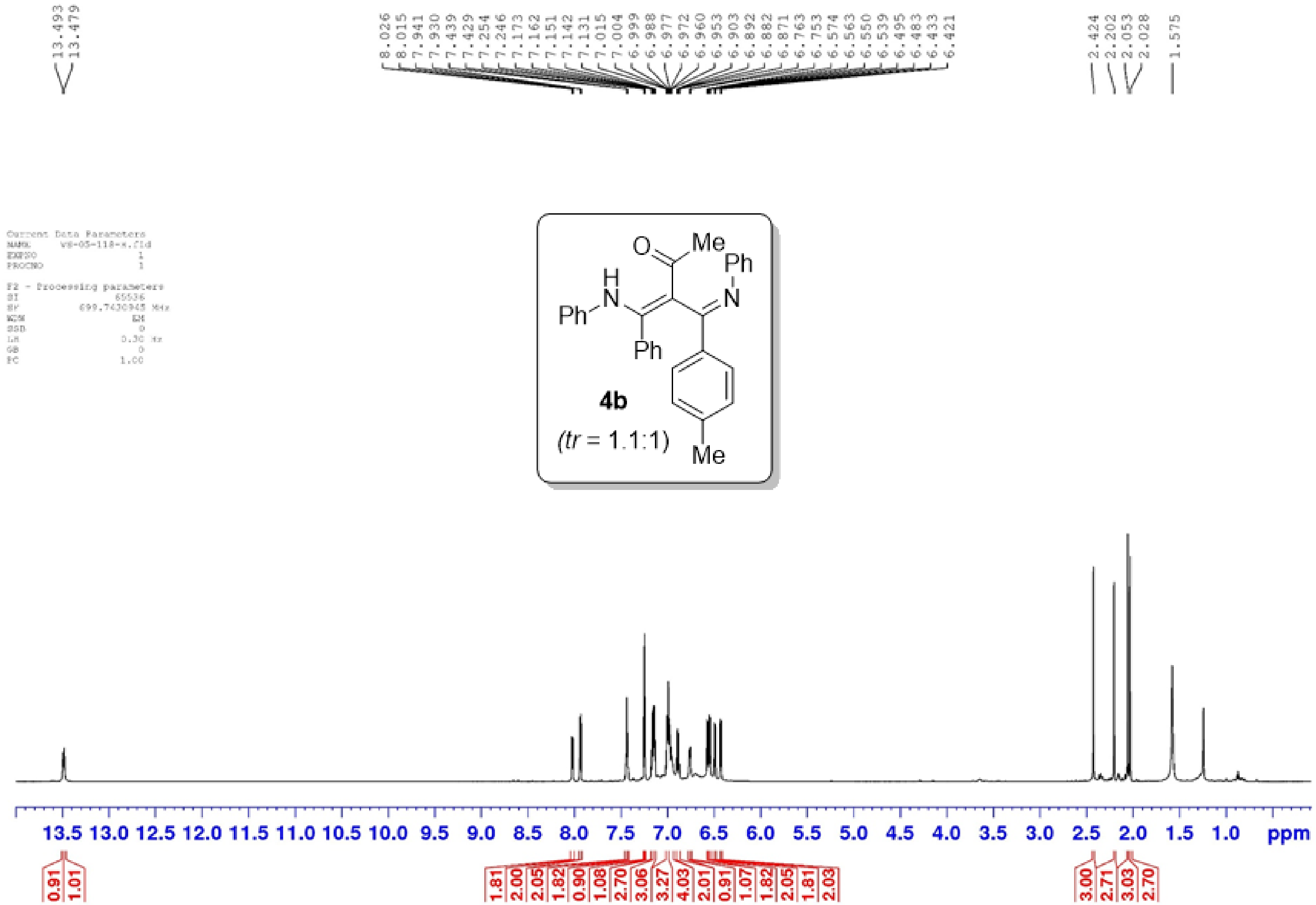

$^{13}C\{1H\}$ **and DEPT NMR (175 MHz,** $CDCl_3$**)**

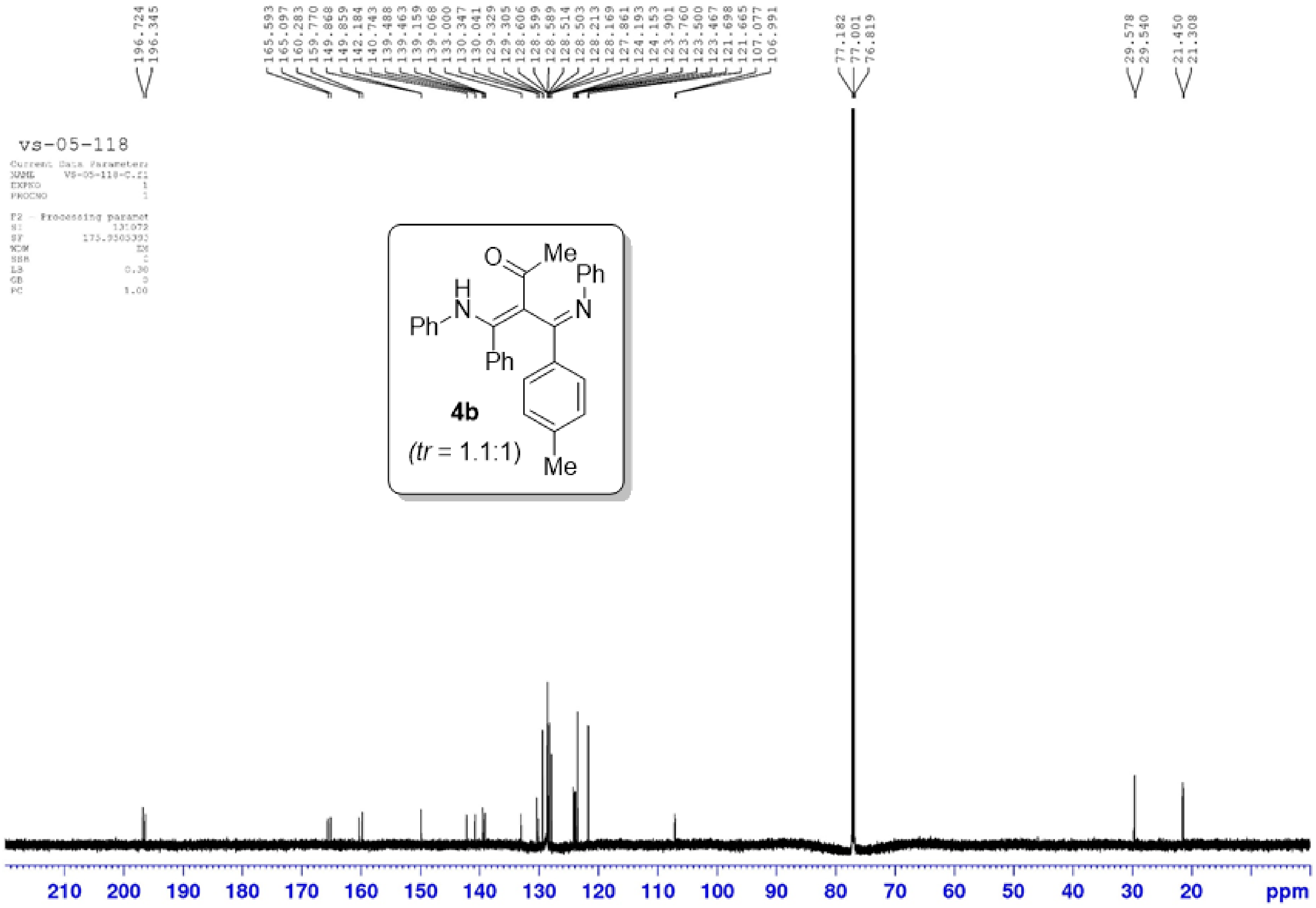

^{1}H-NMR (500 MHz, $CDCl_3$)

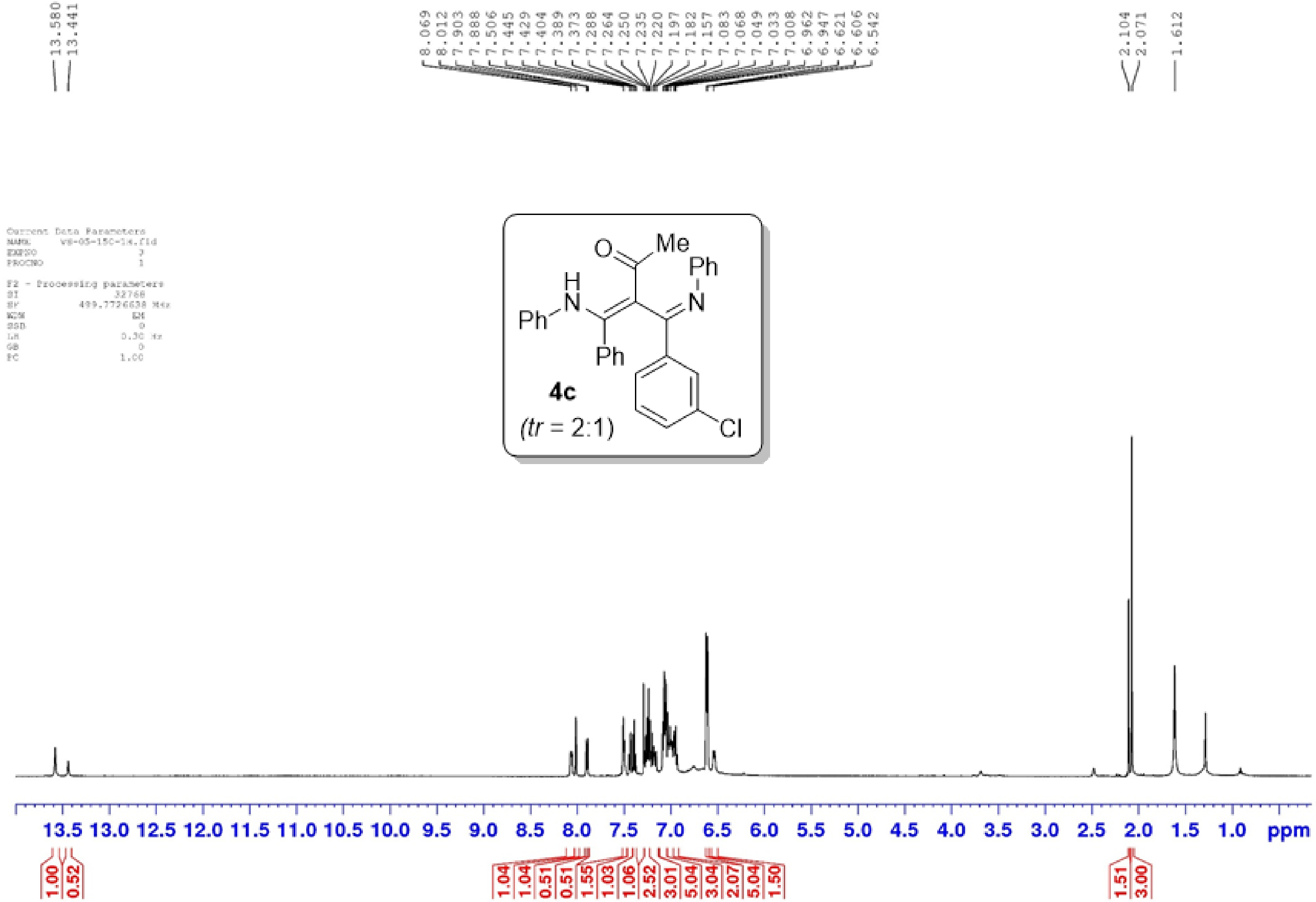

$^{13}C\{1H\}$ **and DEPT NMR (100 MHz, $CDCl_3$)**

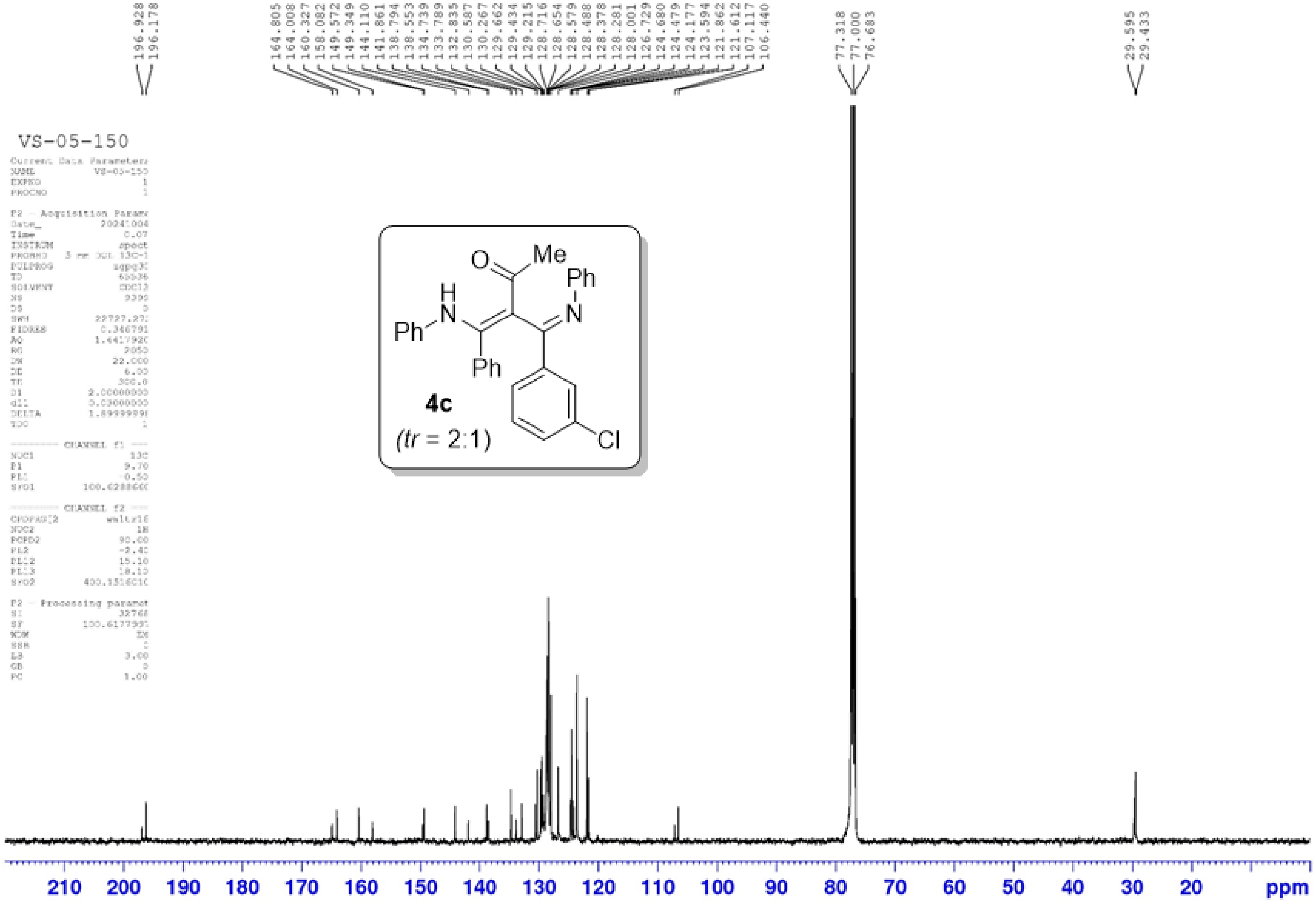

^{1}H-NMR (400 MHz, $CDCl_3$)

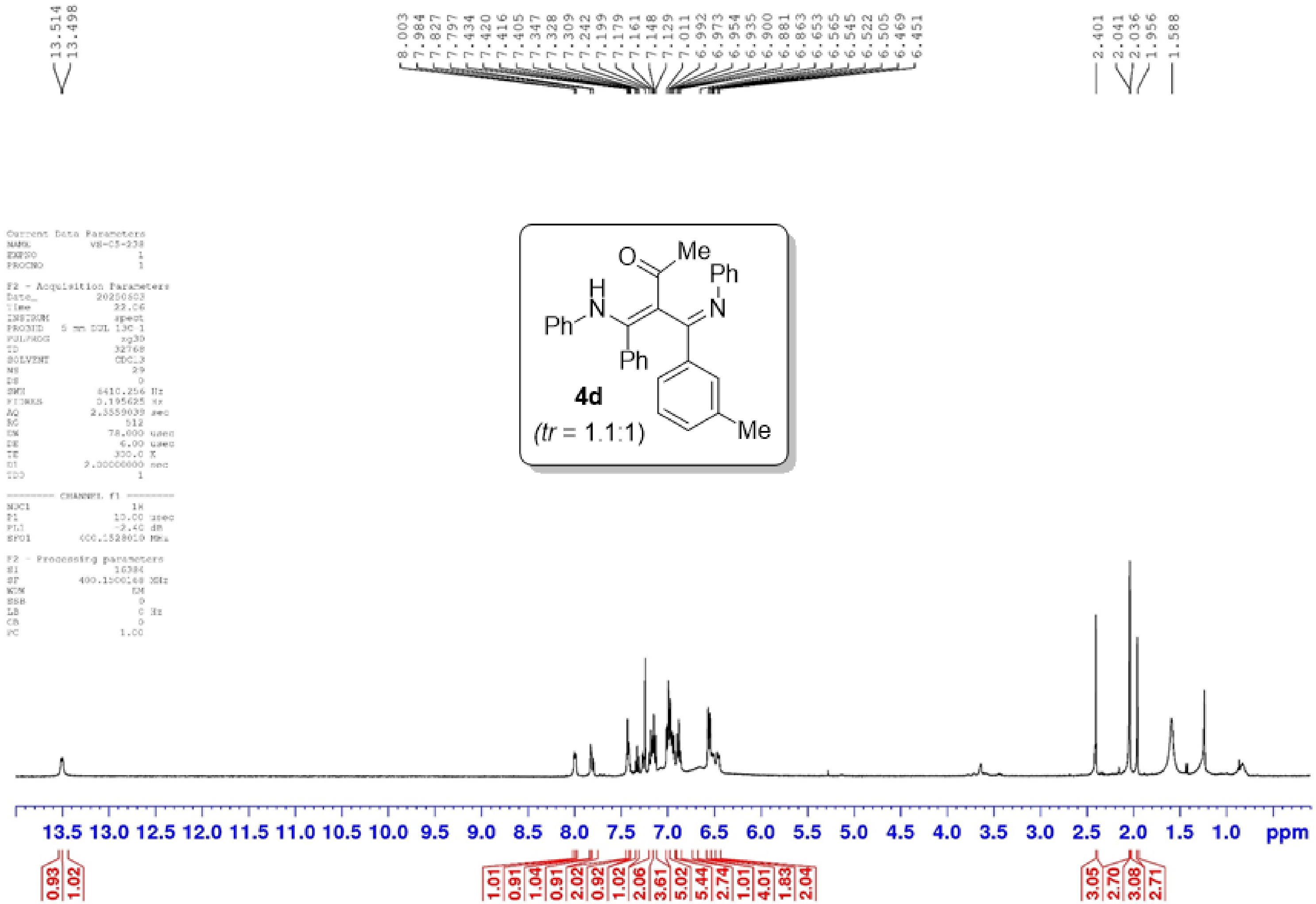

$^{13}C\{1H\}$ and DEPT NMR (125 MHz, $CDCl_3$)

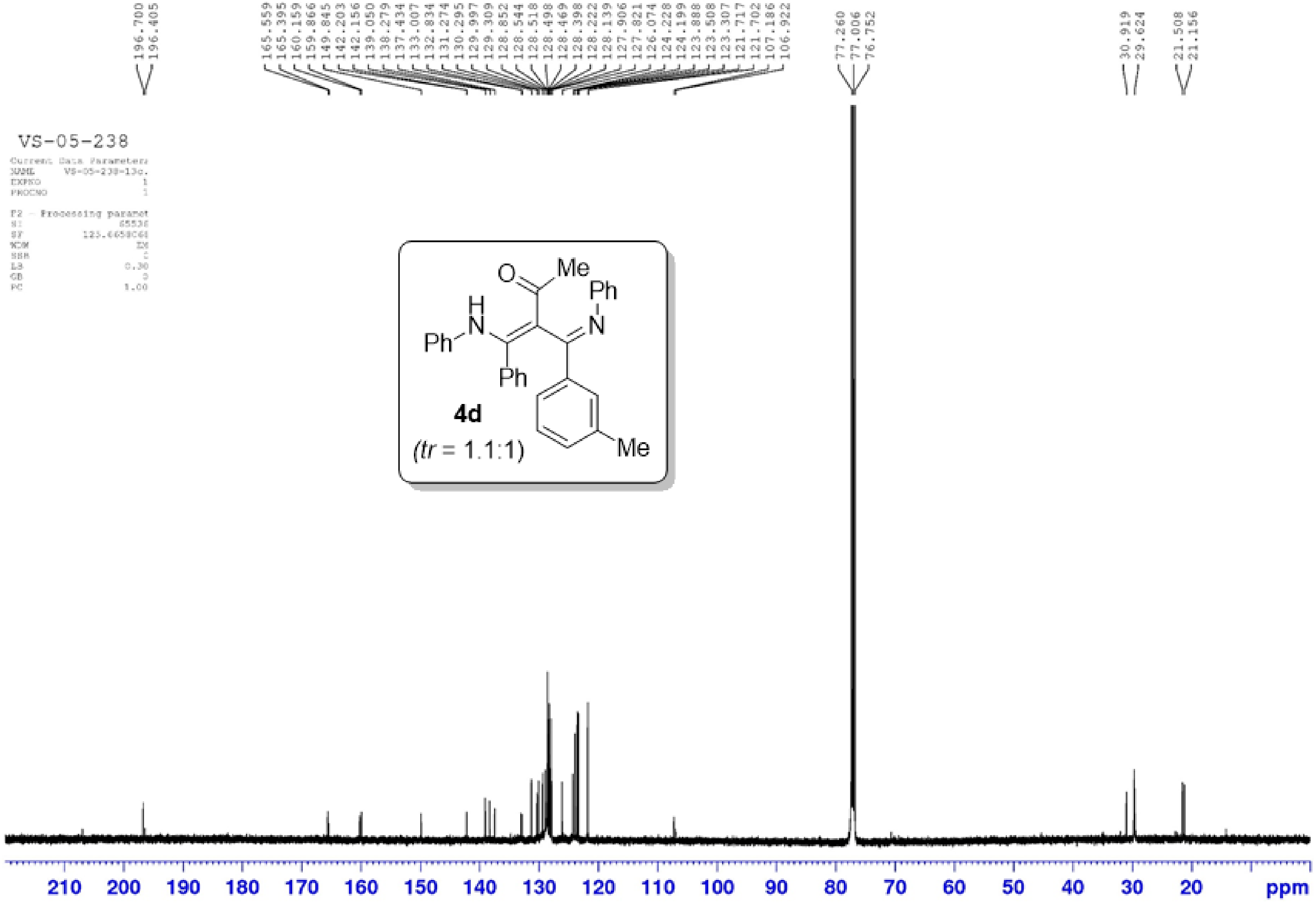

^{1}H-NMR (500 MHz, $CDCl_3$)

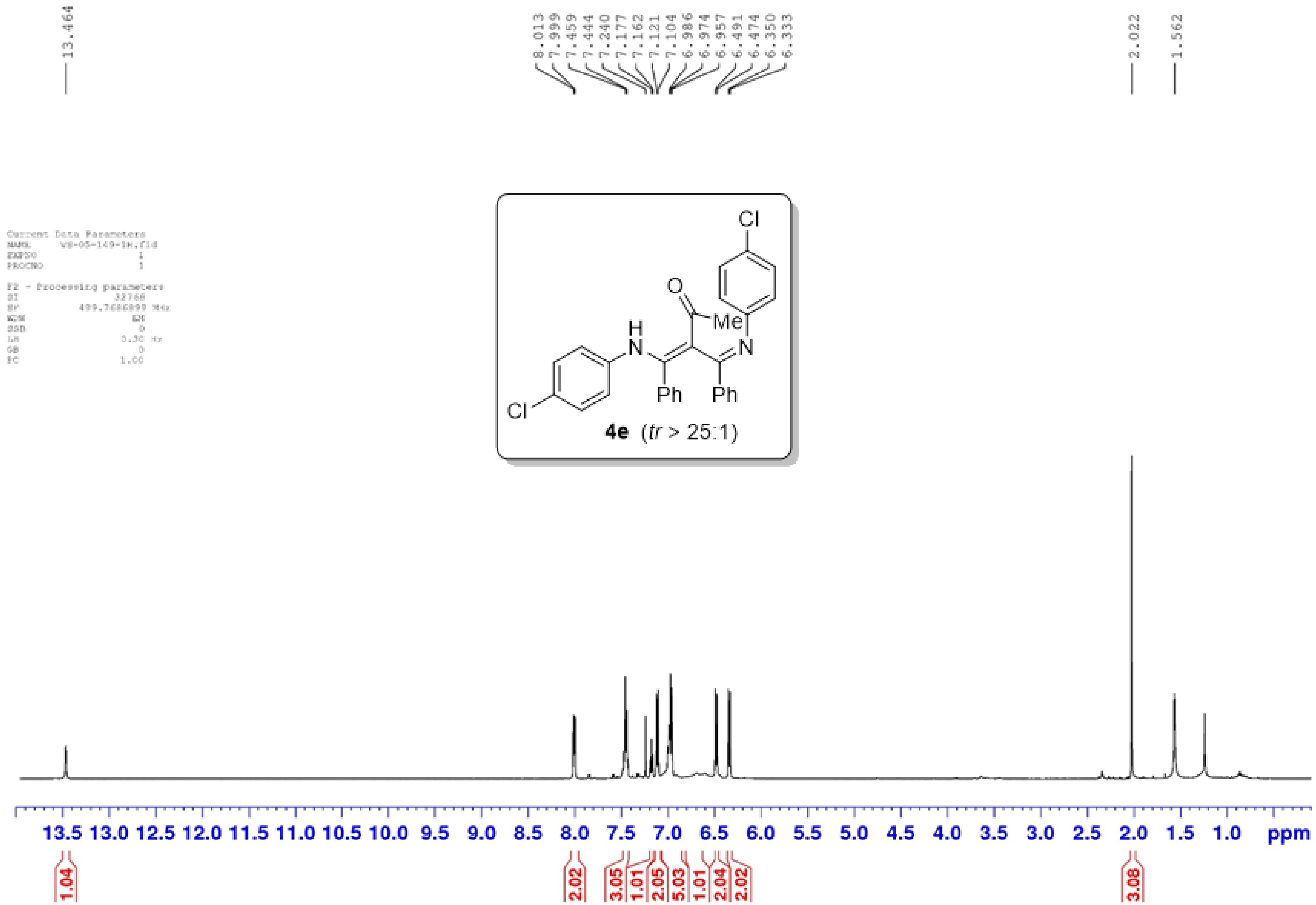

$^{13}C\{1H\}$ and DEPT NMR (100 MHz, $CDCl_3$)

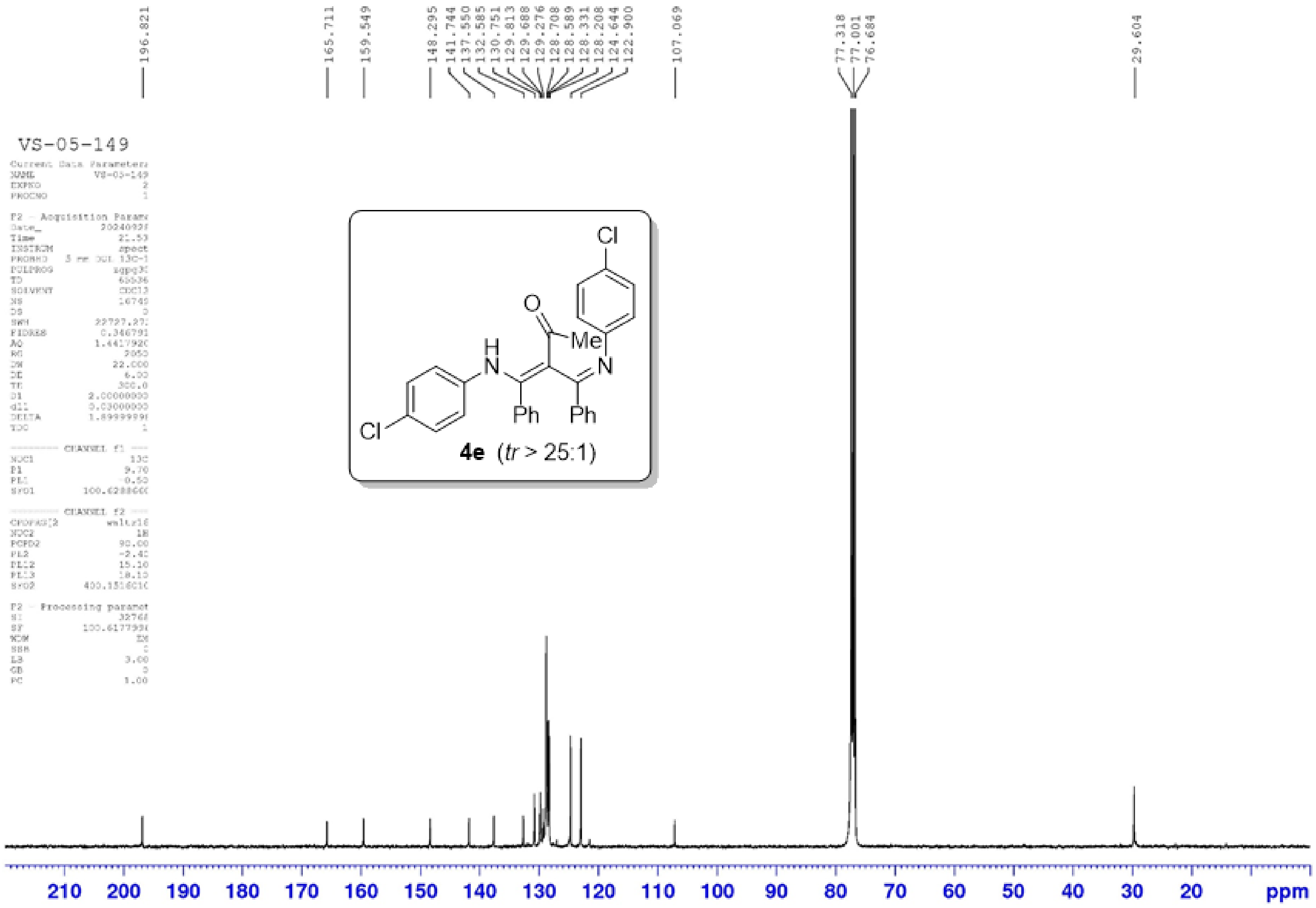

^{1}H-NMR (500 MHz, $CDCl_3$)

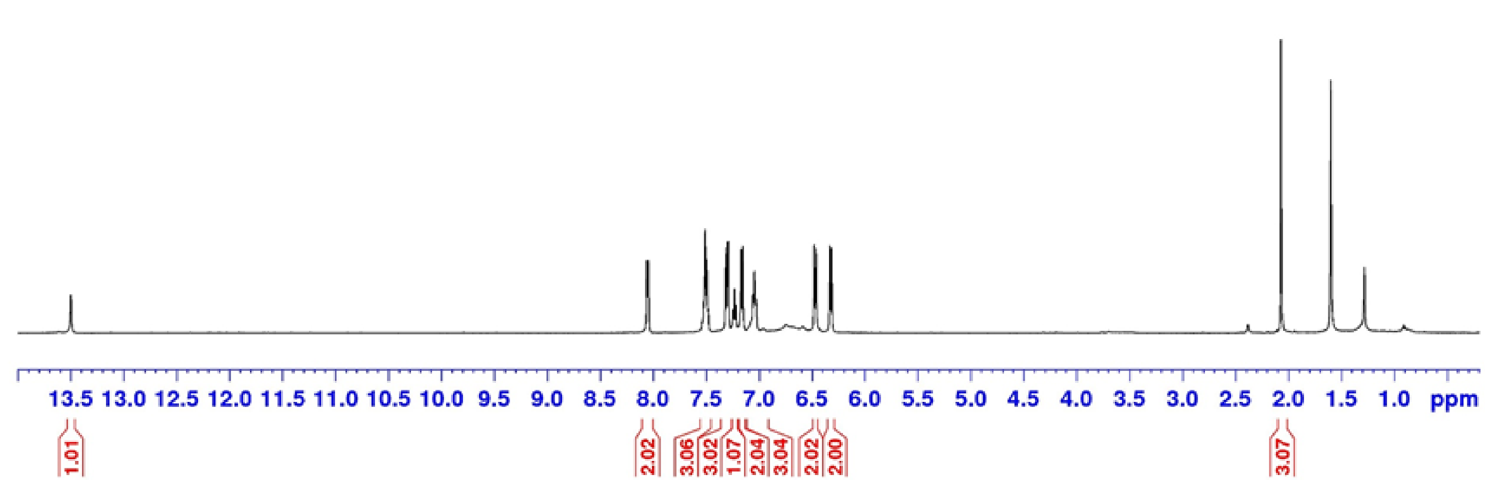

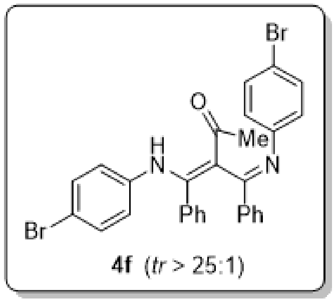

$^{13}C\{1H\}$ and DEPT NMR (125 MHz, $CDCl_3$)

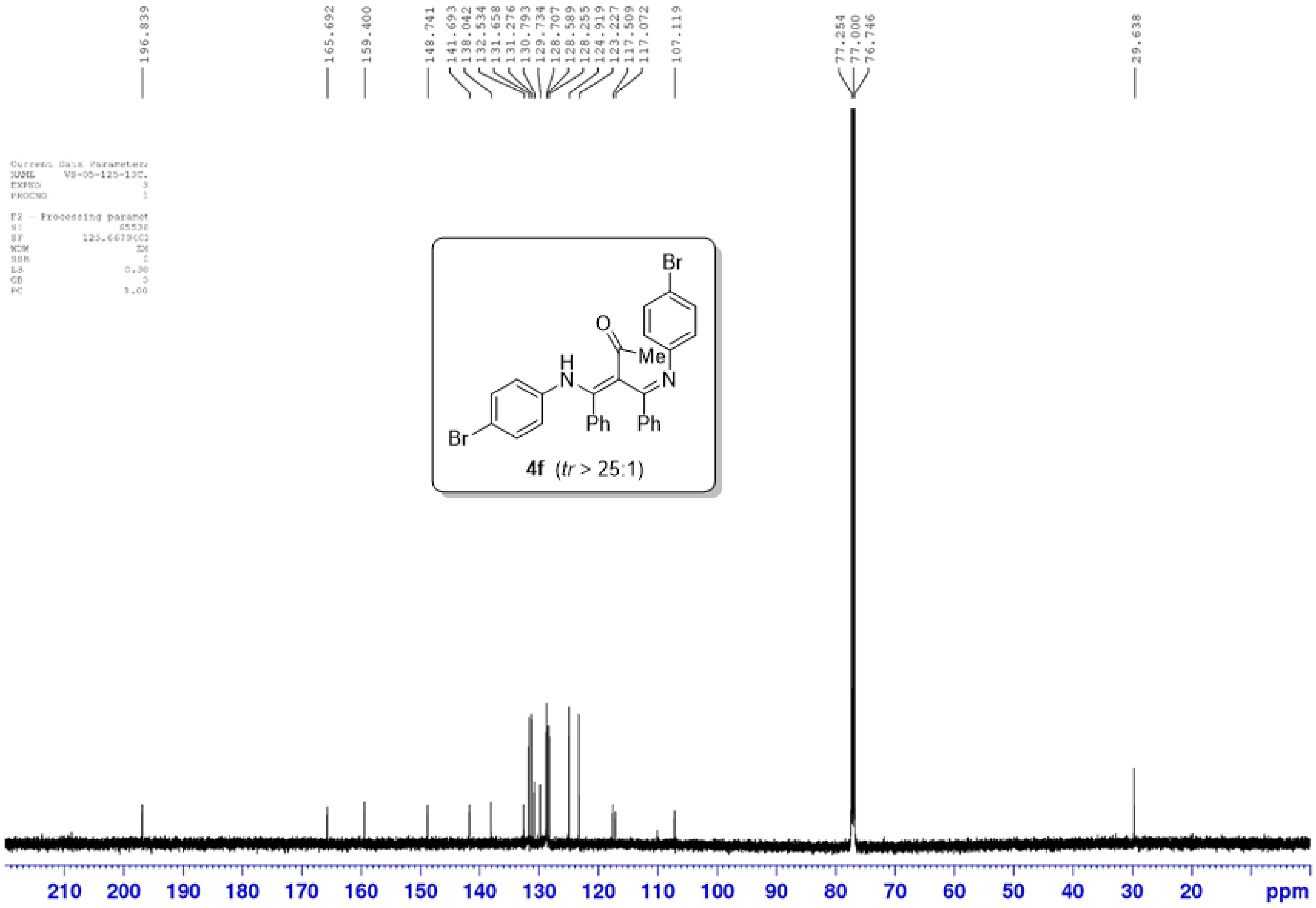

^{1}H-NMR (500 MHz, $CDCl_3$)

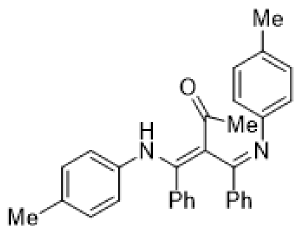

4g (*tr* > 25:1)

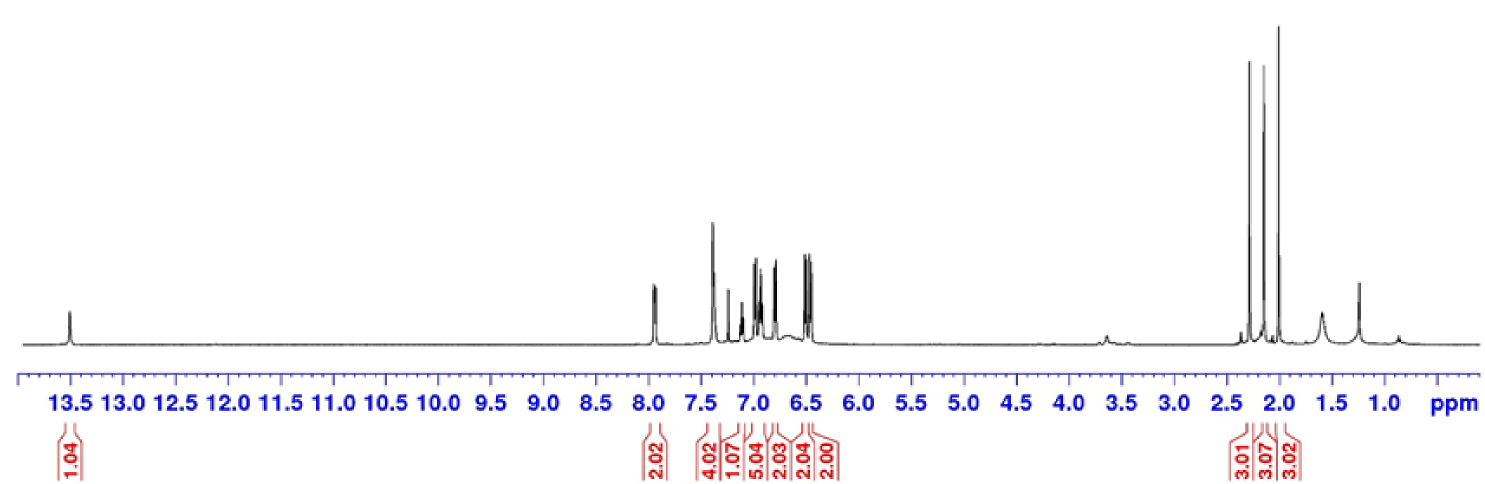

$^{13}C\{1H\}$ and DEPT NMR (100 MHz, $CDCl_3$)

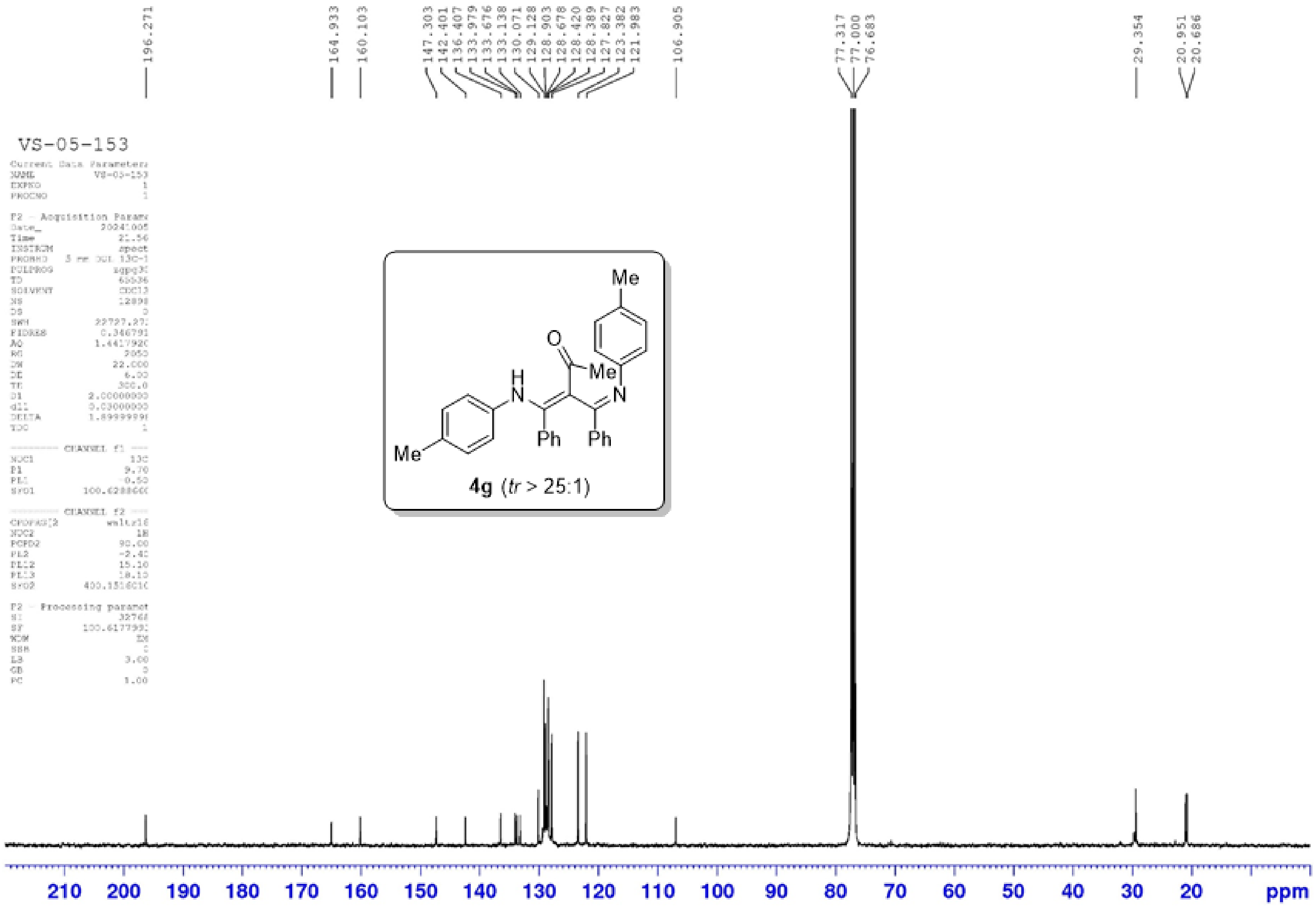

^{1}H-NMR (500 MHz, $CDCl_3$)

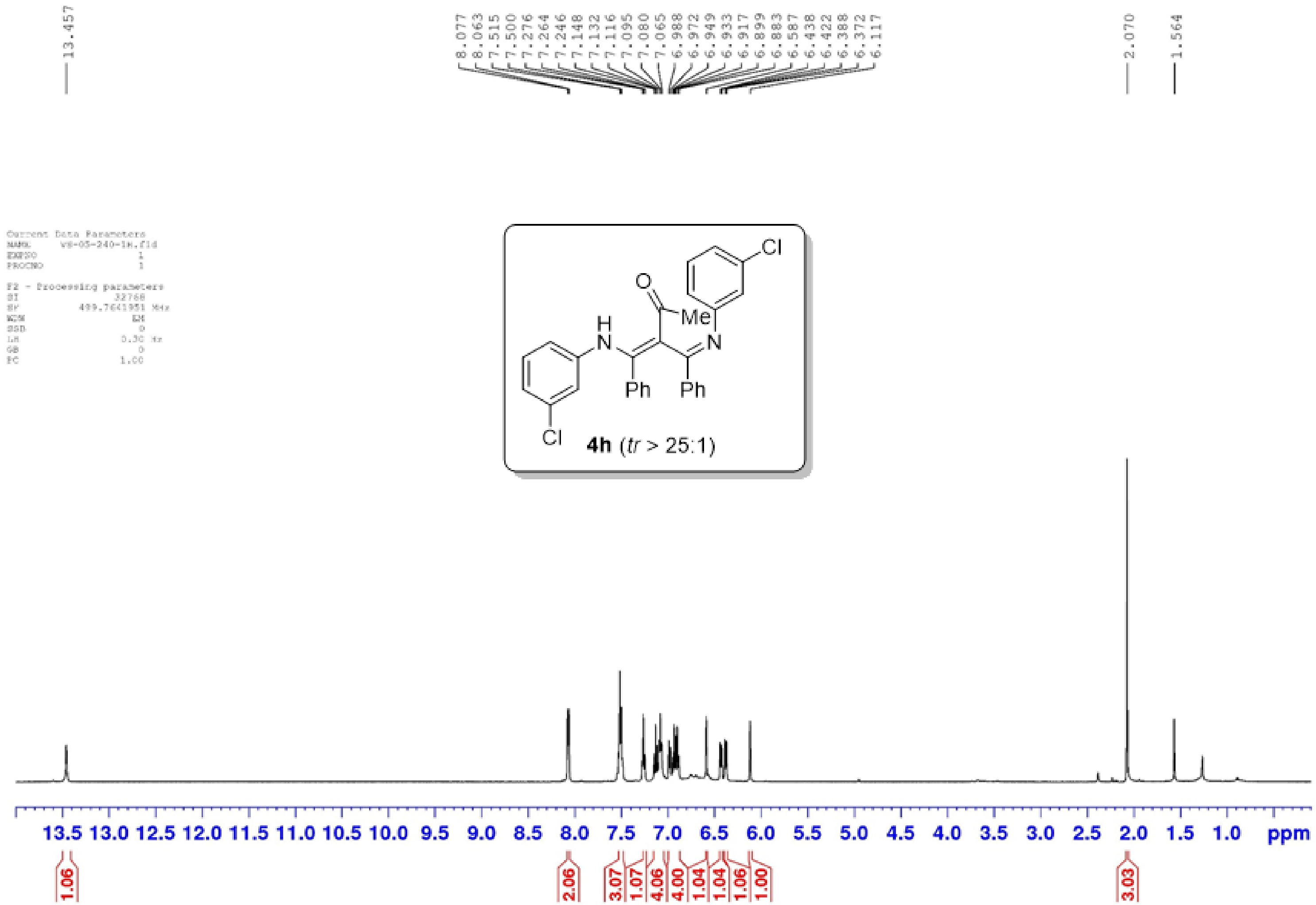

$^{13}C\{1H\}$ **and DEPT NMR (125 MHz, $CDCl_3$)**

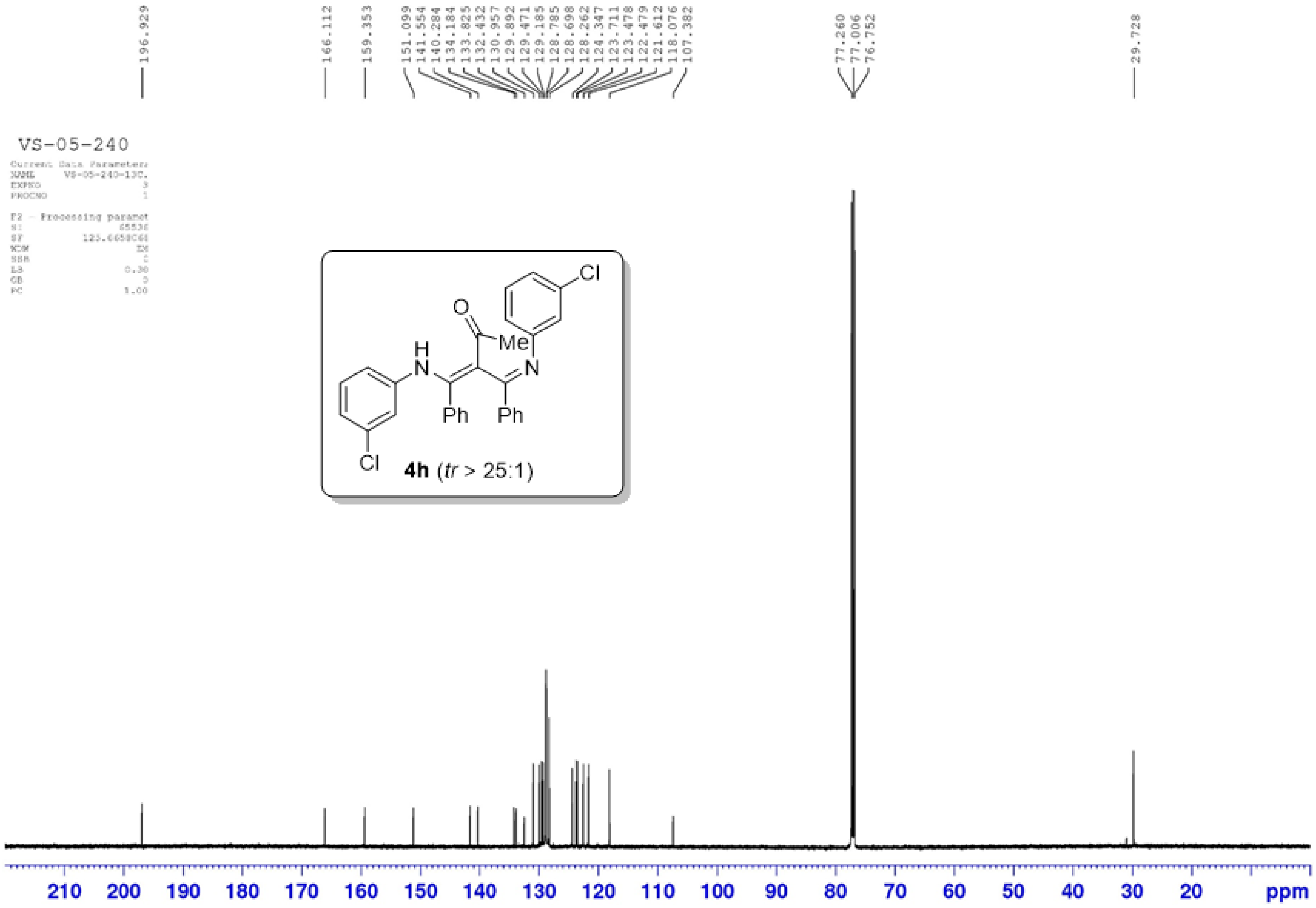

^{1}H-NMR (400 MHz, CDCl$_3$)

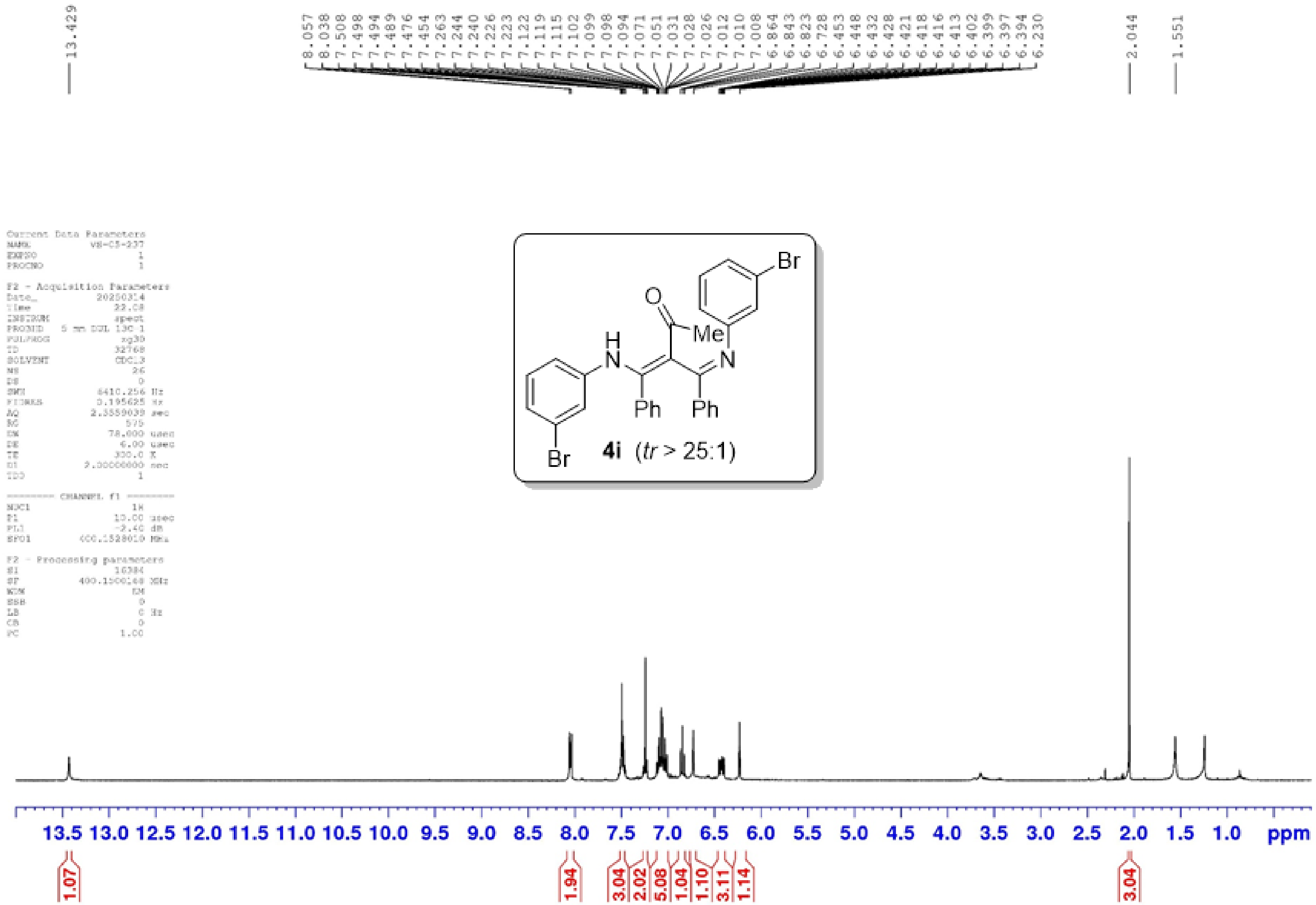

$^{13}C\{1H\}$ and DEPT NMR (100 MHz, $CDCl_3$)

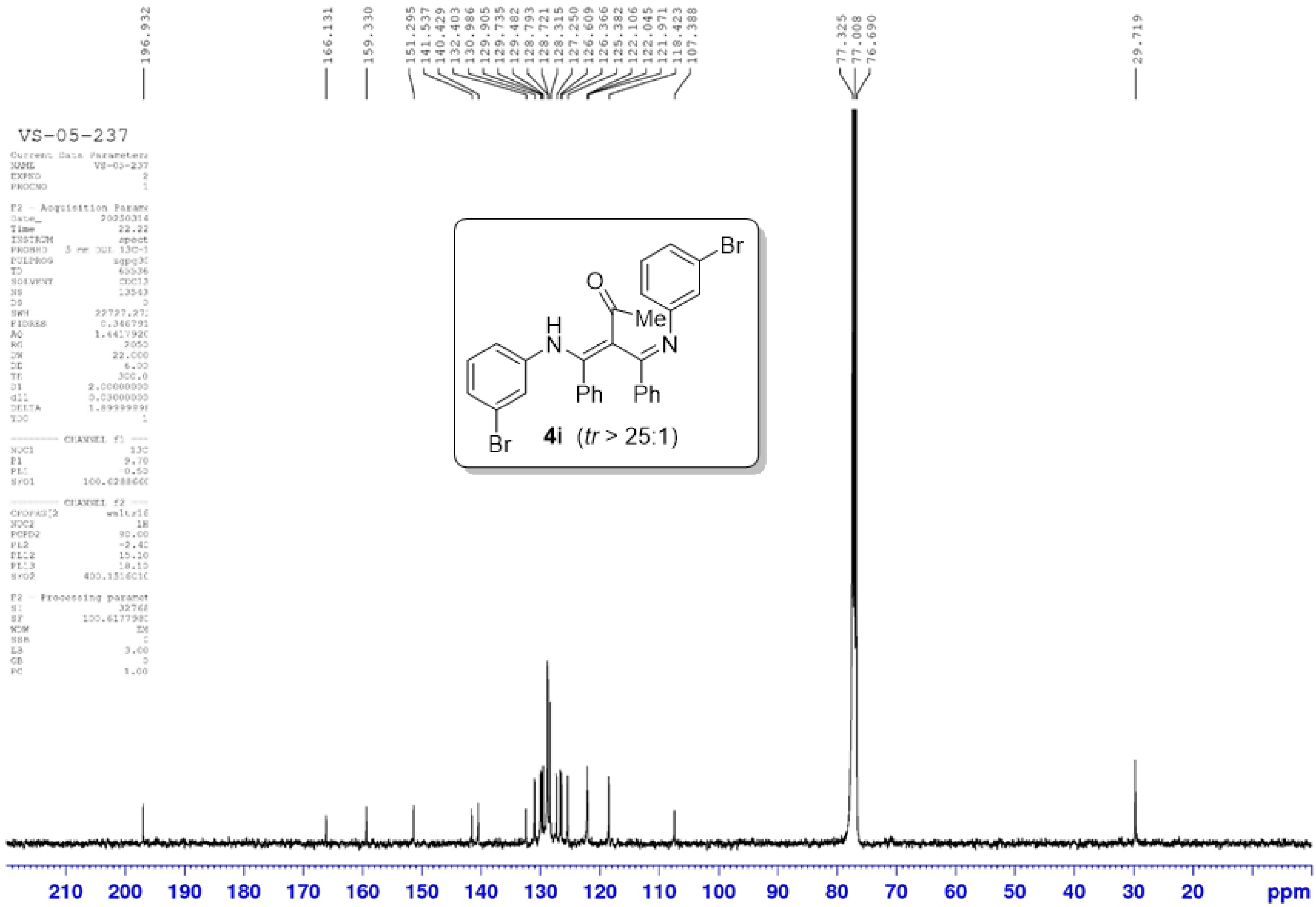

^{1}H-NMR (500 MHz, $CDCl_3$)

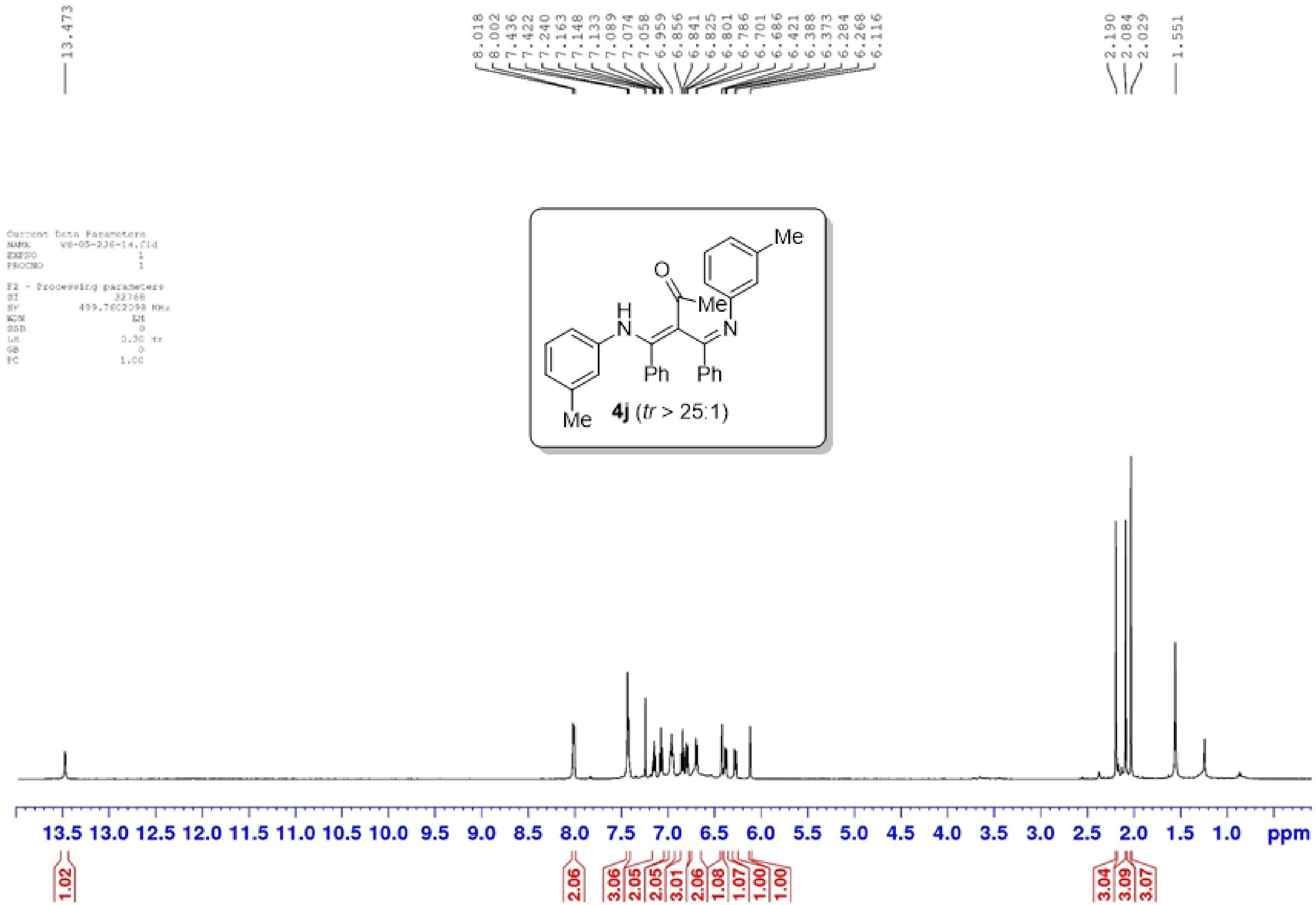

$^{13}C\{1H\}$ and DEPT NMR (125 MHz, $CDCl_3$)

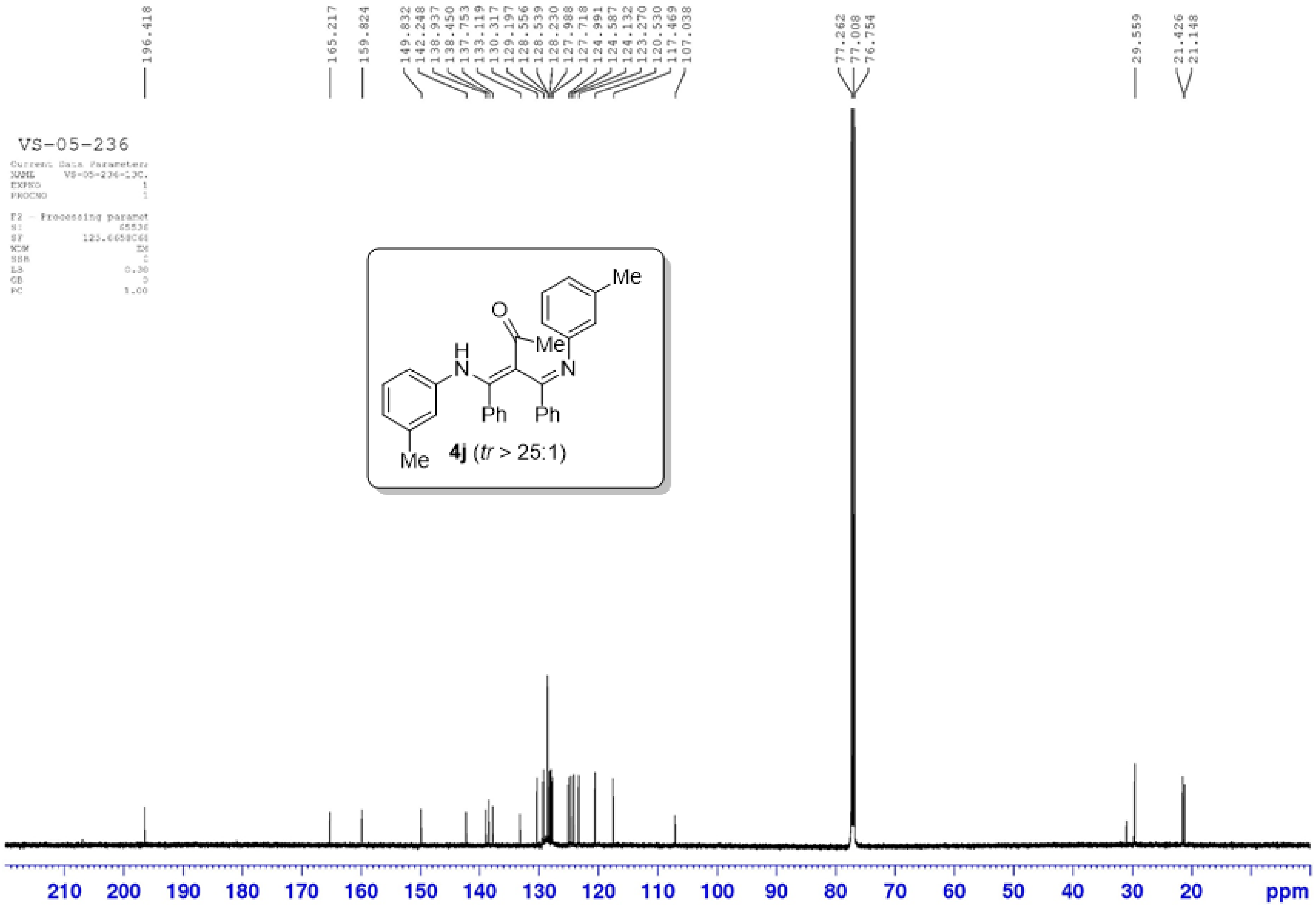

^{1}H-NMR (500 MHz, $CDCl_3$)

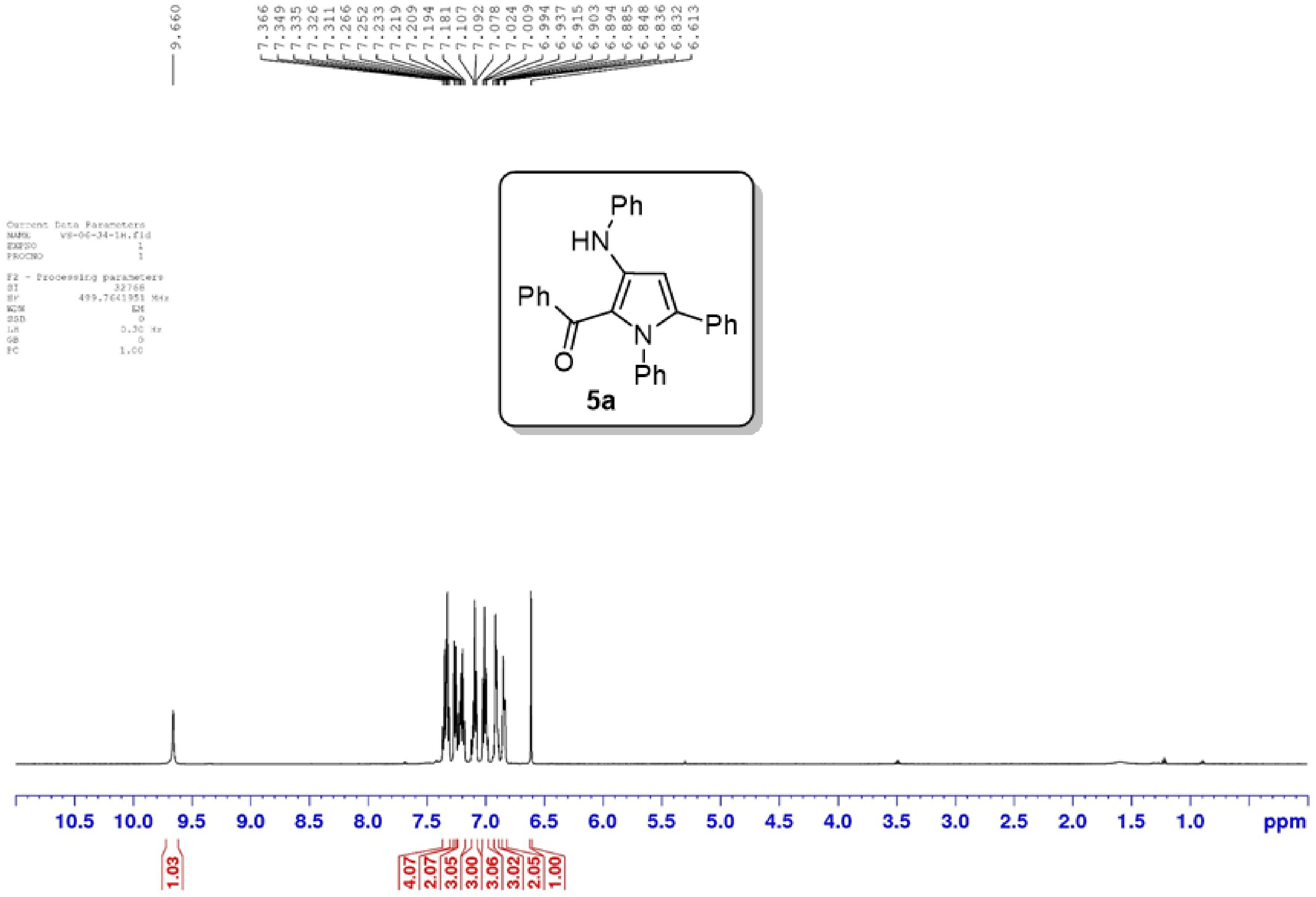

$^{13}C\{1H\}$ and DEPT NMR (175 MHz, $CDCl_3$)

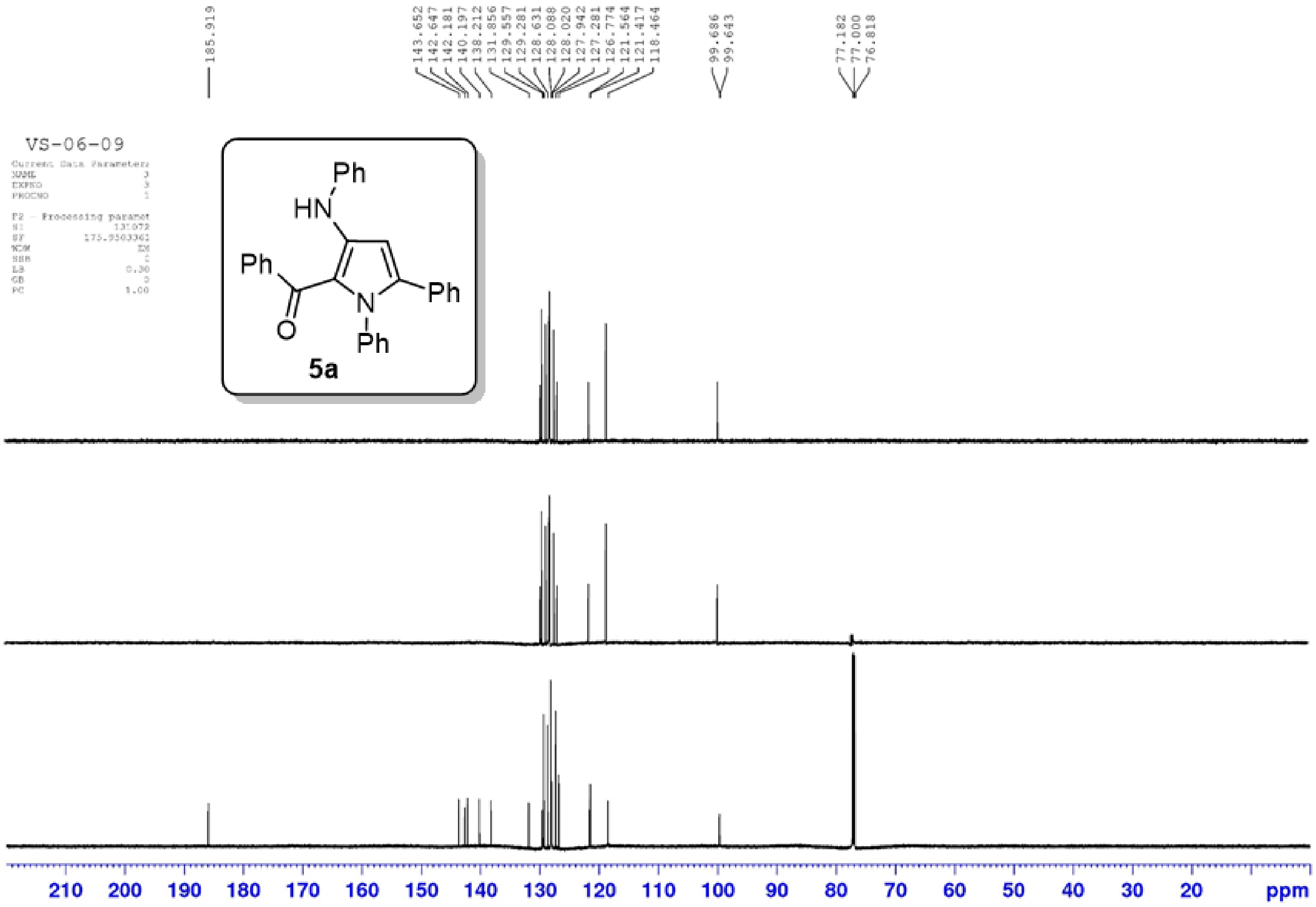

^{1}H-NMR (700 MHz, $CDCl_3$)

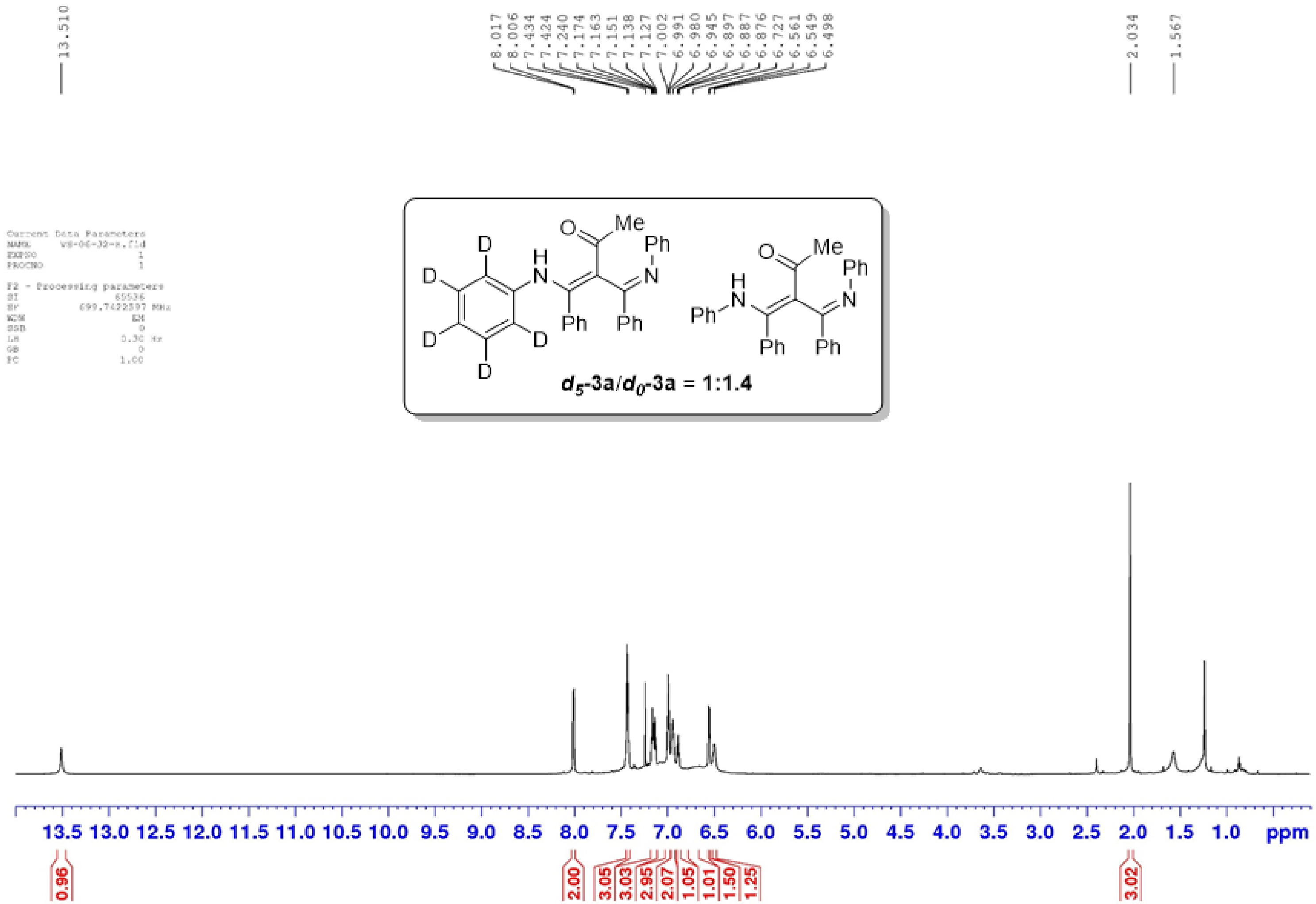

$^{13}C\{1H\}$ and DEPT NMR (100 MHz, $CDCl_3$)

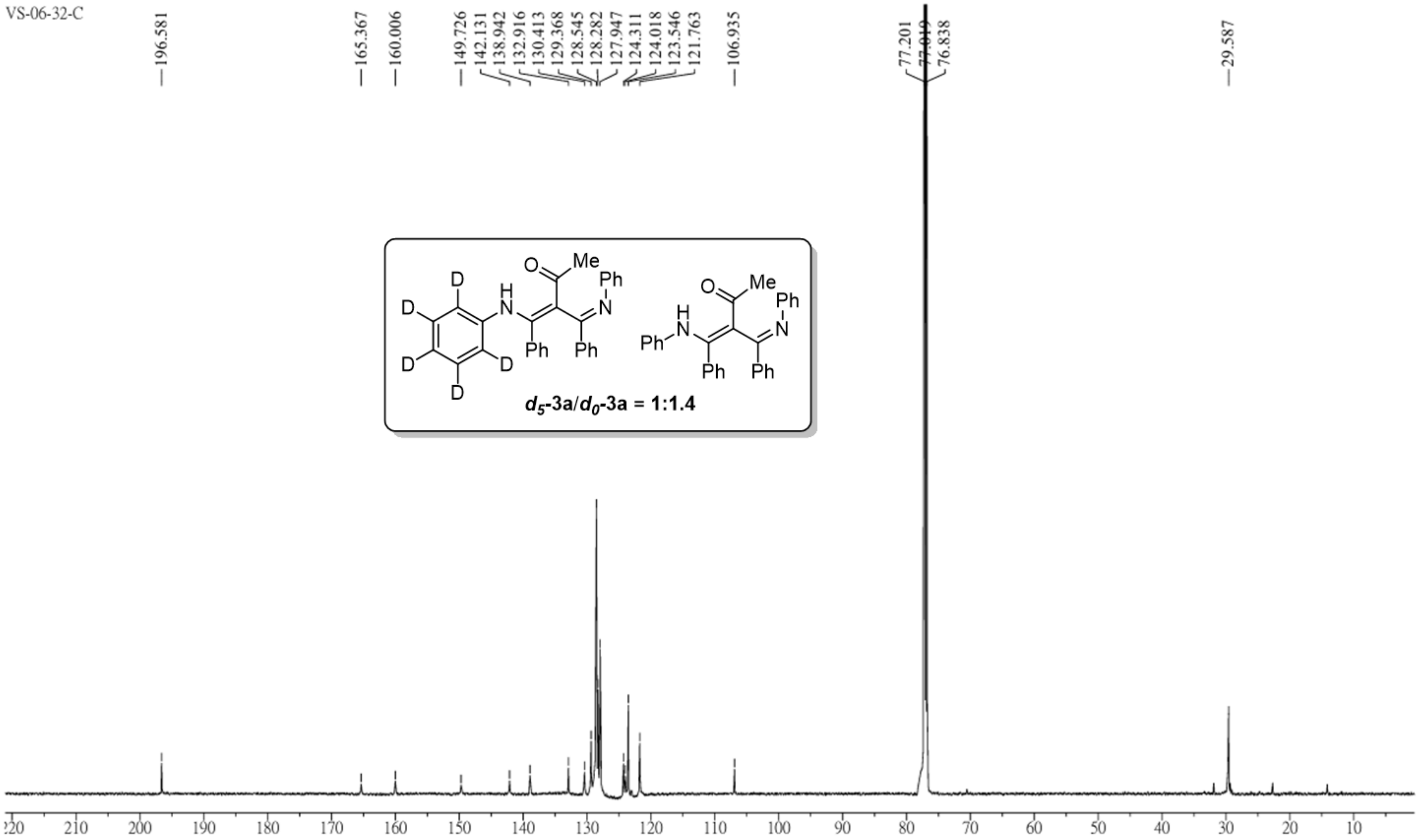

^{1}H-NMR (400 MHz, CDCl$_3$)

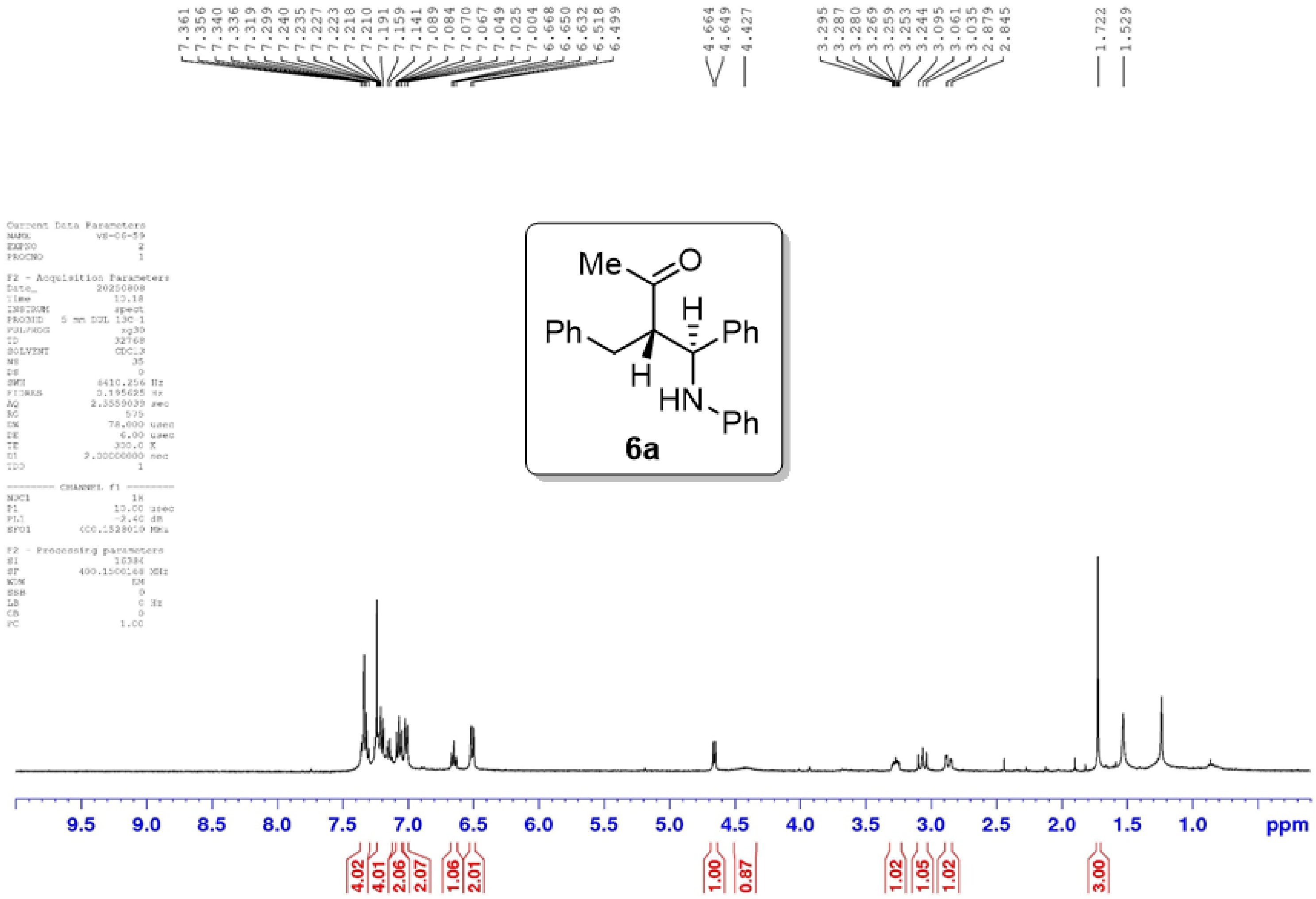
6a

$^{13}C\{1H\}$ and DEPT NMR (100 MHz, $CDCl_3$)

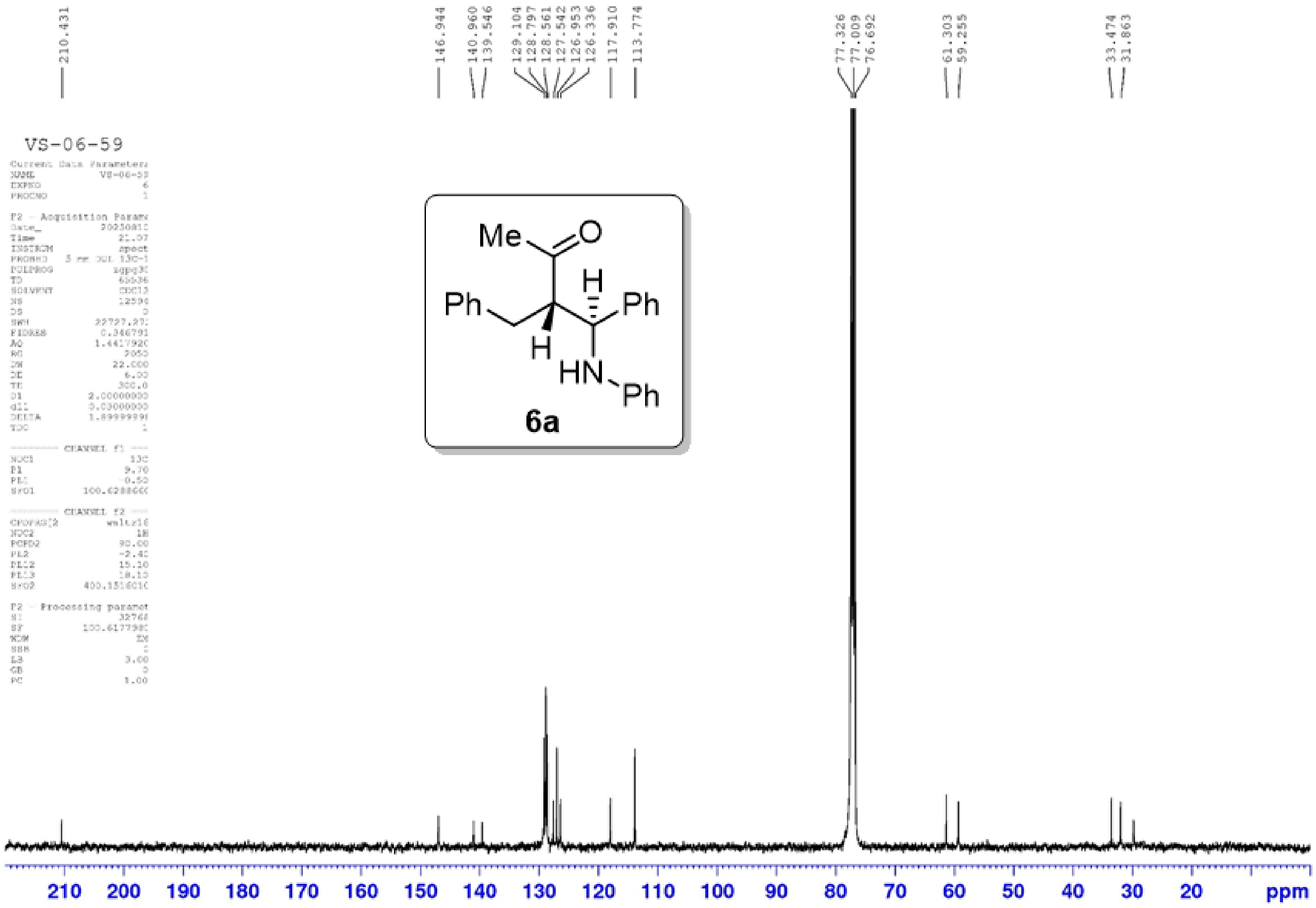

^{1}H-NMR (500 MHz, $CDCl_3$)

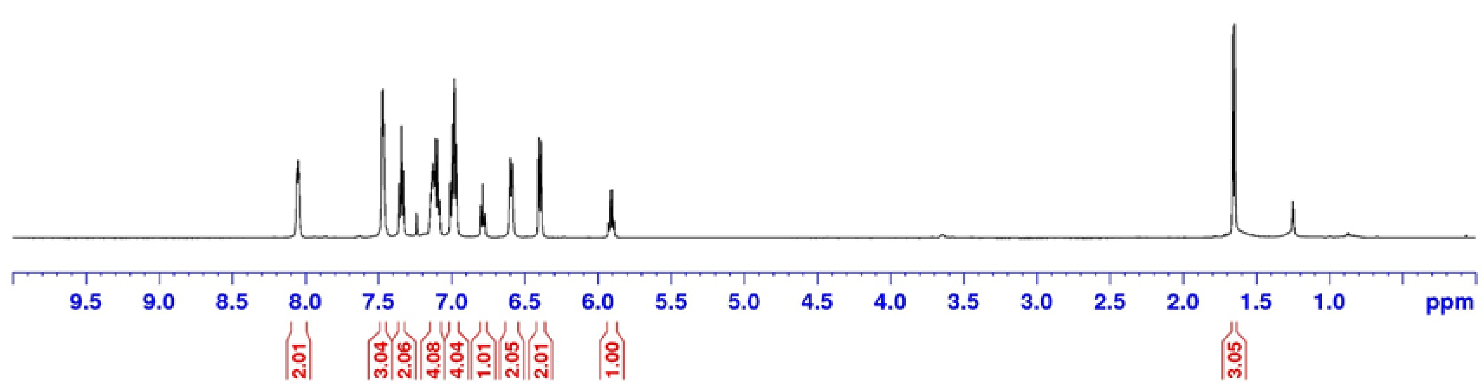

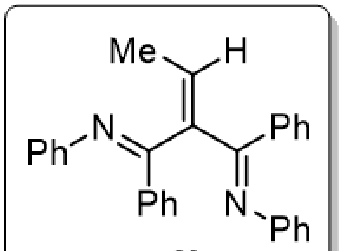

6b

$^{13}C\{1H\}$ and DEPT NMR (175 MHz, $CDCl_3$)

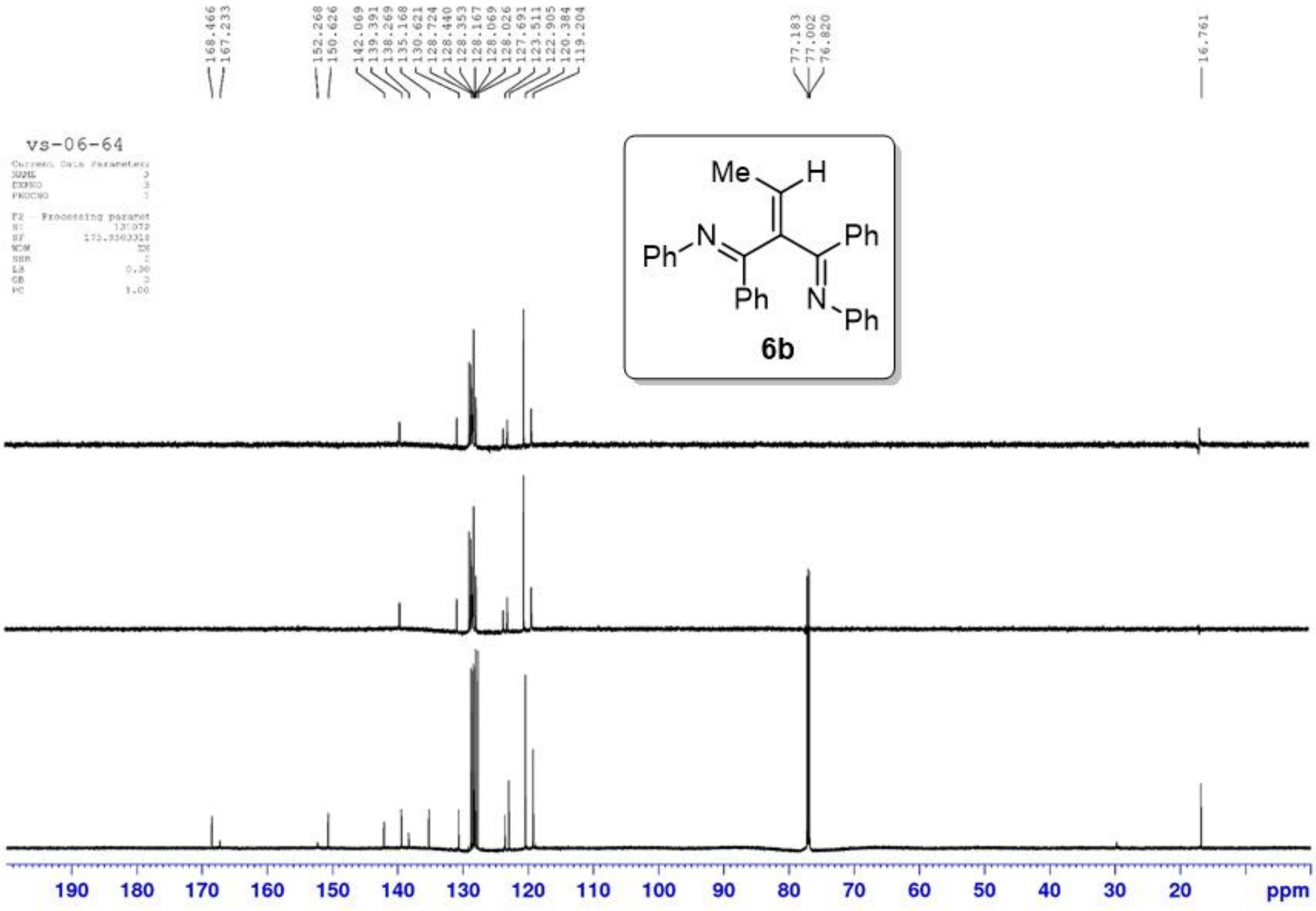

^{1}H-NMR (500 MHz, CDCl$_3$)

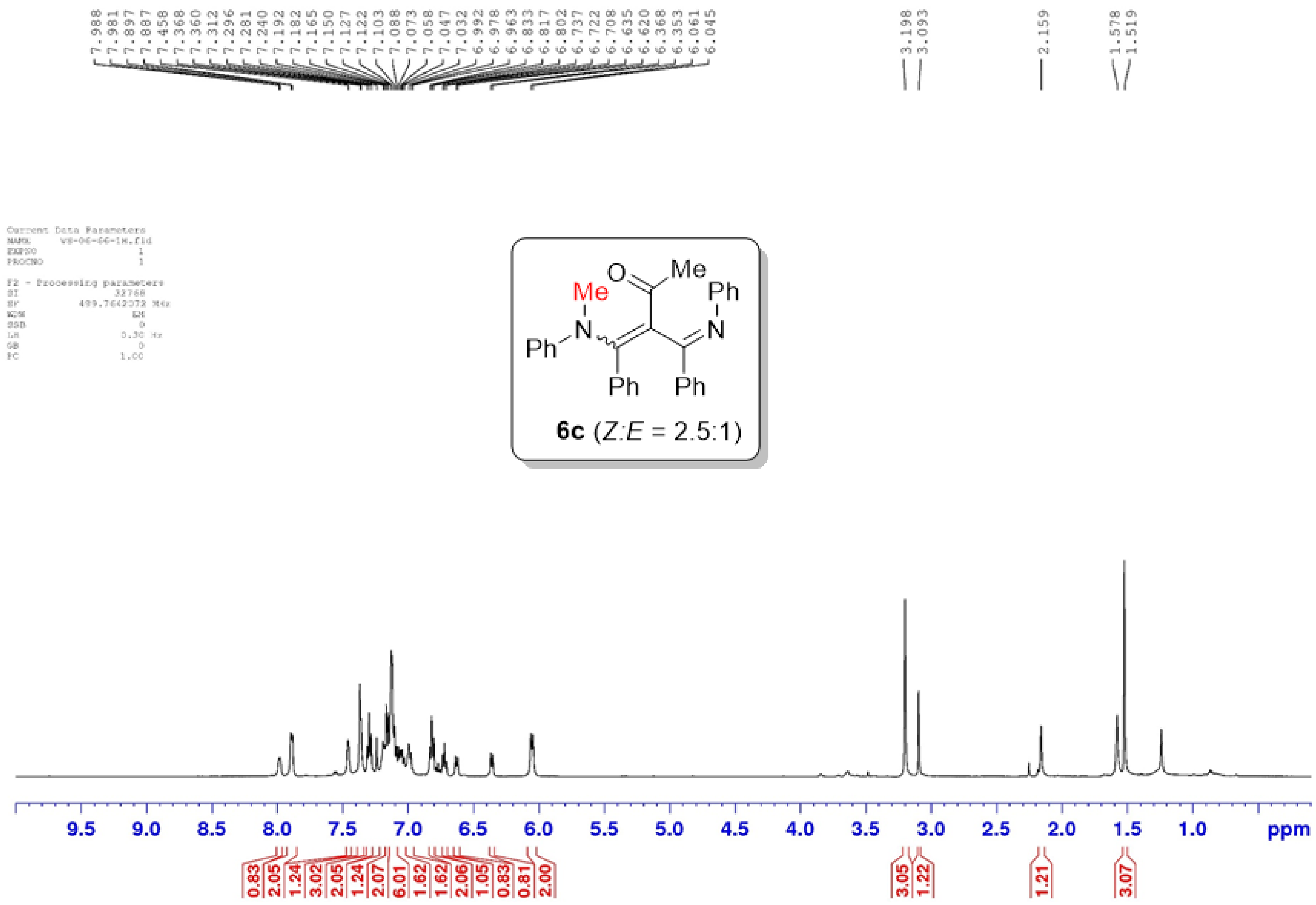

$^{13}C\{1H\}$ and DEPT NMR (125 MHz, $CDCl_3$)

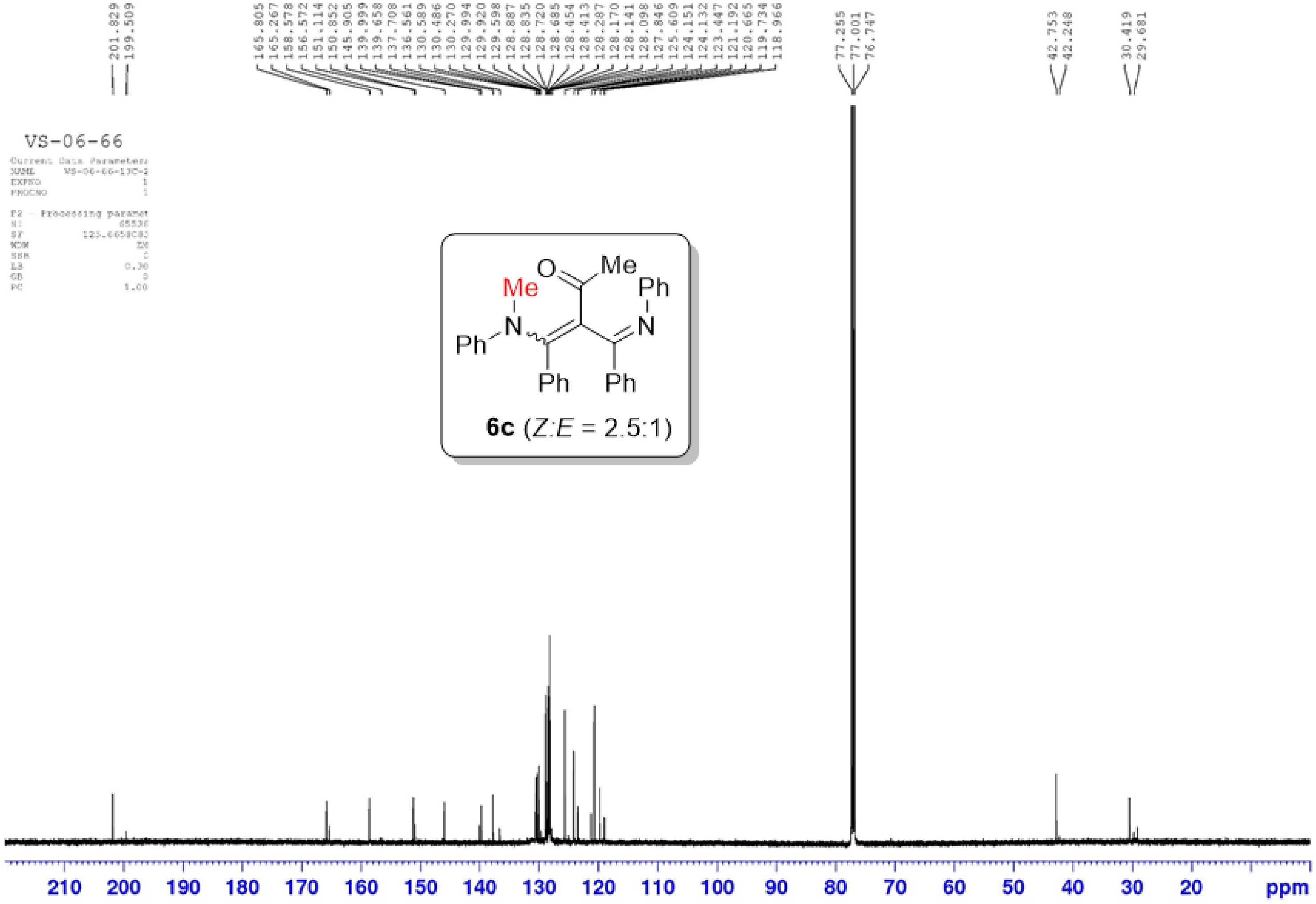

Supplement: Supplementary file 1 [file jo5c02769_si_001.pdf]
